# RPA exhaustion activates SLFN11 to eliminate cells with heightened replication stress

**DOI:** 10.1038/s41556-025-01852-1

**Published:** 2026-01-09

**Authors:** Tyler H. Stanage, Shudong Li, Sandra Segura-Bayona, Aurora I. Idilli, Rhona Millar, Graeme Hewitt, Simon J. Boulton

**Affiliations:** 1 DSB Repair Laboratory, The Francis Crick Institute, London, UK; 2 Cancer Research UK Radnet City of London Centre, UCL Cancer Institute, London, UK; 3 School of Cancer and Pharmaceutical Sciences, Comprehensive Cancer Centre, Kings’s College London, London, UK

**Keywords:** Apoptosis, Stalled forks

## Abstract

SLFN11 is epigenetically silenced and confers chemoresistance in half of all cancers. In response to replication stress, SLFN11 triggers translation shutdown and p53-independent apoptosis, but how DNA damage activates SLFN11 remains unclear. Here through CRISPR-based screens we implicate SLFN11 as the critical determinant of cisplatin sensitivity in cells lacking primase–polymerase (PrimPol)-mediated repriming. SLFN11 and the downstream integrated stress response uniquely promote cisplatin-driven apoptosis in PrimPol-deficient cells. We demonstrate that replication protein A (RPA) exhaustion and single-stranded DNA exposure trigger SLFN11 activation and cell death when PrimPol is inactivated. We further identify the USP1–WDR48 deubiquitinase complex as a positive modulator of SLFN11 activation in PrimPol-deficient cells, revealing an addiction to the Fanconi anaemia pathway to resolve cisplatin lesions. Finally, we demonstrate that rapid RPA exhaustion on chemical inhibition of DNA polymerase α activates SLFN11-dependent cell death. Together, our results implicate RPA exhaustion as a general mechanism to activate SLFN11 in response to heightened replication stress.

## Abstract (web summary)

Stanage et al. identify a role for transfer RNA nuclease SLFN11 in replication-stress-induced cell death in cisplatin-treated cells lacking PrimPol. SLFN11 is activated upon single-stranded DNA accumulation at stalled forks followed by replication protein A exhaustion and cell death.

## Main

DNA replication is constantly challenged by numerous endogenous and exogenous sources of DNA damage yet is an inherently accurate and efficient process^1–4^. Replication stress invoked by DNA lesions is sensed by the ataxia telangiectasia and Rad3-related (ATR)-dependent intra-S-phase checkpoint, which is activated by the accumulation of RPA-bound single-stranded (ss)DNA at stalled forks and triggers a cascade of events that regulate origin firing, replication fork stabilization and lesion repair or bypass^5–8^. Indeed, multiple interdependent DNA damage, repair and tolerance pathways recognize, respond to and repair impediments at stalled replication forks. Recent work also uncovered the phenomenon of RPA exhaustion at stalled forks during heightened replication stress, which leads to exposure of ssDNA, fork breakage and irreversible replication catastrophe^9,10^. How cells respond to RPA exhaustion independently of ATR signalling is currently unknown.

Owing to their inherent plasticity, stalled replication forks can be remodelled or components of the replisome modified to differentially recruit factors required to cope with specific types of fork blockages^2,11^. Stalled replication forks can also be directly restarted by DNA primer synthesis downstream of fork blockages via the primase–polymerase PrimPol^12–17^. In vitro, PrimPol-mediated repriming can occur in as little as 14 nt downstream of a replication-blocking lesion^15^, which could limit ssDNA accumulation at stalled forks caused by helicase–polymerase uncoupling^18,19^. PrimPol reprimes downstream of diverse sources of replication fork blockage ranging from endogenous G-quadruplexes and R-loops to chain-terminating nucleotides, bulky base adducts induced by ultraviolet light, benzo[*a*]pyrene-diol-epoxide (BPDE) and interstrand and intrastrand crosslinks induced by mitomycin C (MMC) and cisplatin^14,15,18,20–27^.

Cisplatin is a commonly used chemotherapeutic that targets rapidly dividing cancer cells by creating intrastrand and interstrand DNA crosslinks that stall active replication forks^28^. The major pathway responsible for repairing cisplatin-induced interstrand crosslinks is the Fanconi anaemia (FA) pathway, which comprises over 20 gene products, many of which are shared with homologous recombination (HR) and translesion DNA synthesis (TLS)^29^. Initiation of the FA pathway depends on recruitment of the FA core complex comprising 14 proteins, followed by ATR-dependent phosphorylation and FANCL-dependent ubiquitination of the FANCD2–FANCI complex^30,31^. FANCD2–FANCI ubiquitination (and FA pathway activation) is tightly regulated by the deubiquitinase (DUB) complex USP1–WDR48 (also known as USP1–UAF1) (ref. ^32^). Intriguingly, a recent study proposed a potential role for PrimPol repriming in an interstrand crosslink tolerance pathway^25^. Paradoxically, despite evidence that PrimPol can reprime synthesis downstream of cisplatin DNA lesions in cells, loss of PrimPol alone does not broadly confer sensitivity to cisplatin^14,20,22,33^. A satisfactory explanation for this discrepancy is currently lacking and highlights the current challenge in understanding how PrimPol activity cooperates with other pathways at stalled forks.

Innate and acquired resistance to cisplatin occurs frequently in patients through various mechanisms, including increased drug efflux, increased DNA repair capacity and loss of proapoptotic pathways^34,35^. Indeed, previous work demonstrated a role for increased expression of PrimPol in the adaptive response to cisplatin treatment in *BRCA*-deficient tumours^20^. One of the strongest prognostic markers of cisplatin efficacy is the expression of Schlafen 11 (SLFN11), a transfer (t)RNA nuclease that induces p53-independent apoptosis in response to DNA damage^36–41^. Intriguingly, SLFN11 is epigenetically silenced in half of all treatment-naive cancer cell lines, leading to chemoresistance^42–44^. SLFN11 induces irreversible replication fork arrest through a range of proposed mechanisms, including prevention of origin firing via CDT1 degradation, increasing local chromatin accessibility and inhibition of HR or checkpoint responses^37,45–47^. After fork arrest, SLFN11 cleaves type II tRNAs, leading to ribosome stalling at rare UUA leucine codons^38,48,49^. Ribosome stalling results in activation of the general control non-derepressible 2 (GCN2)-dependent integrated stress response (ISR) and JNK-mediated ribotoxic stress response, triggering p53-independent apoptosis^38,39^. Importantly, the ssDNA-binding and tRNA nuclease activities of SLFN11 are absolutely required for activation of p53-independent apoptosis^38^. However, despite understanding how SLFN11 activation triggers apoptosis, the DNA lesion that activates SLFN11 remains unknown.

Here we sought to identify drivers of cisplatin cytotoxicity using the chronic myelogenous leukaemia eHAP cell line as a model system. Surprisingly, our targeted CRISPR–Cas9 screens identified loss of PrimPol as conferring cisplatin sensitivity. In contrast to FA, HR and TLS deficiencies, cisplatin sensitivity of PrimPol knockout (KO) cells uniquely depends on SLFN11. We show that cells deficient in PrimPol-mediated repriming or drugs that induce ssDNA accumulation at replication forks potently activate an SLFN11-dependent and GCN2-dependent cell death. Using PrimPol deficiency as a model system to study SLFN11 activation, we identified RPA exhaustion as the primary mechanism of SLFN11 activation in response to DNA damage. Furthermore, we implicated USP1-dependent downregulation of the FA pathway as the mechanism that induces RPA exhaustion and SLFN11-dependent cell death, specifically when PrimPol is inactivated. Finally, we identified DNA polymerase α inhibition as a potent inducer of RPA exhaustion and SLFN11-dependent cell death. We proposed that, through its activation by RPA exhaustion, SLFN11 maintains genome stability by triggering the elimination of cells experiencing heightened replication stress, a hallmark of early cancer cells.

## Results

### Loss of PrimPol-mediated repriming confers cytotoxicity to bulky base adducts

**Fig. 1:**
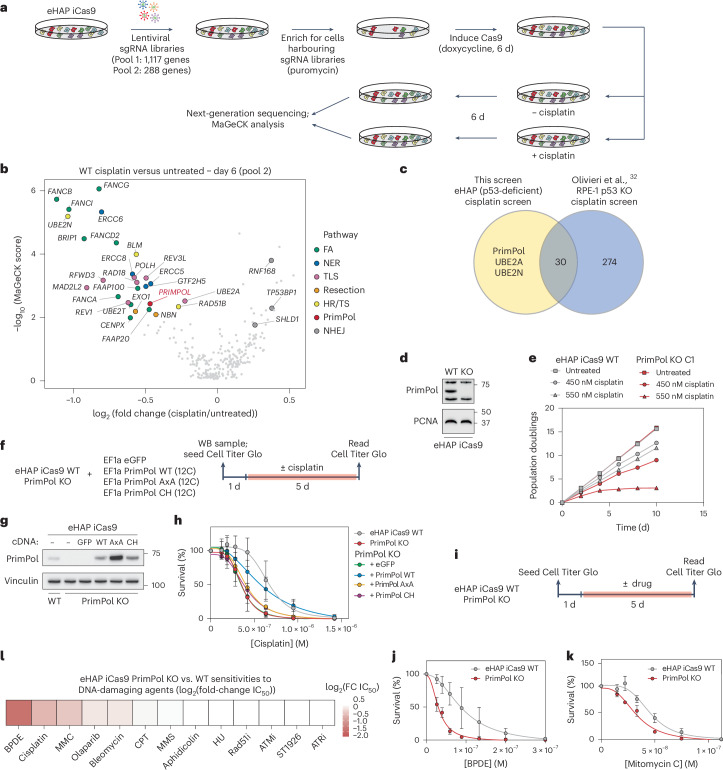
Loss of the PrimPol confers cytotoxicity to bulky base adducts. **a**, An experimental scheme depicting how targeted CRISPR–Cas9 screens performed. Two separate screens were performed in biological triplicates utilizing pool 1 sgRNA library (1,117 genes targeted) or pool 2 sgRNA library (288 genes targeted). **b**, A volcano plot depicting a measure of statistical significance (−log_10_(MaGeCK score)) plotted against log_2_(fold-change) in abundance of sgRNAs targeting indicated genes in cisplatin-treated versus untreated conditions in eHAP iCas9 WT cells infected with the pool 2 sgRNA library. Labelled genes are coloured based on the DDR pathway in which they operate. **c**, A Venn diagram comparing genes scoring as significantly depleted in cisplatin-treated arms of the screen depicted in **b** performed in eHAP cells (yellow) and those in a genome-wide CRISPR–Cas9 dropout screen performed in RPE-1 p53 KO cells (blue) in ref. ^33^. **d**, A western blot depicting loss of PrimPol protein in an isogenic eHAP iCas9 PrimPol KO clone. **e**, Population doublings of eHAP iCas9 WT (grey) and PrimPol KO (red) cells plotted against time to demonstrate cell growth in the absence (squares) or presence of 450 nM (circles) or 550 nM (triangles) cisplatin. Growth curve experiments were performed in *n* = 2 biological replicates. Data are presented as means. **f**, Experimental scheme for a Cell Titer Glo viability assay to determine the cisplatin sensitivity of eHAP iCas9 WT or PrimPol KO cells complemented with WT PrimPol, PrimPol AxA (catalytic mutant Asp114Ala, Glu116Ala) or PrimPol CH (primase mutant Cys419Gly, His426Tyr). **g**, A western blot depicting PrimPol protein levels in eHAP iCas9 WT cells or PrimPol KO cells complemented with WT PrimPol, PrimPol AxA or PrimPol CH mutants. Exogenous protein levels were downregulated using the misFIT expression tuner system. **h**, Cell viability measured using the Cell Titer Glo assay on treatment of the indicated doses of cisplatin over 5 d. These experiments were performed in *n* = 4 biological replicates. Data are presented as mean ± s.d. **i**, Experimental scheme for a Cell Titer Glo assay to determine cell viability of eHAP iCas9 WT or PrimPol KO cells challenged with the indicated drugs. **j**, Cell viability measured after challenging eHAP iCas9 WT or PrimPol KO cells with the indicated doses of BPDE. Experiments were performed in *n* = 3 biological replicates. Data are presented as mean ± s.d. **k**, Cell viability measured after challenging eHAP iCas9 WT or PrimPol KO cells with the indicated doses of MMC. Experiments were performed in *n* = 3 biological replicates. Data are presented as mean ± s.d. **l**, A heatmap depicting sensitivity of eHAP iCas9 PrimPol KO versus WT cells to various DNA-damaging agents and inhibitors to DDR proteins. Sensitivities were determined by computing the log_2_(fold-change) between half-maximal inhibitory concentration (IC_50_) values of PrimPol KO and WT cells. Source numerical data and unprocessed blots are available in the source data. ATMi, ataxia-telangiectasia mutated (ATM) inhibitor; ATRi, ATR inhibitor; FC, fold-change; HU, hydroxyurea; MMS, methyl methane sulfonate; Rad51i, Rad51 inhibitor; TS, template switching. Source data

To investigate the pathways required for cisplatin lesion metabolism at stalled replication forks, we performed a targeted CRISPR–Cas9 dropout screen in the diploid inducible Cas9 (iCas9) eHAP cell line (Fig. 1a). Two single guide (sg)RNA libraries (Supplementary Table 1) were utilized to interrogate genes associated with chromatin and telomere maintenance (pool 1) and the DNA damage response (DDR; pool 2). To determine the efficacy of our screens, we first compared sgRNA counts in wild-type (WT) cells infected with pool 2 treated with cisplatin to an untreated condition using the MaGeCK algorithm^50^ (Fig. 1b). As observed previously^33^, sgRNAs targeting genes belonging to the FA, nucleotide excision repair, TLS, DNA end-resection and HR pathways resulted in marked sensitivity to cisplatin (Fig. 1b). Surprisingly, we also identified loss of PrimPol, the primase–polymerase that catalyses repriming downstream of leading strand fork blockages, as also conferring sensitivity to cisplatin. This contrasts with previously published reports suggesting that loss of PrimPol alone does not confer sensitivity to cisplatin, despite its well-established role in DNA damage tolerance^14,20,22,33^.

**Extended Data Fig. 1:**
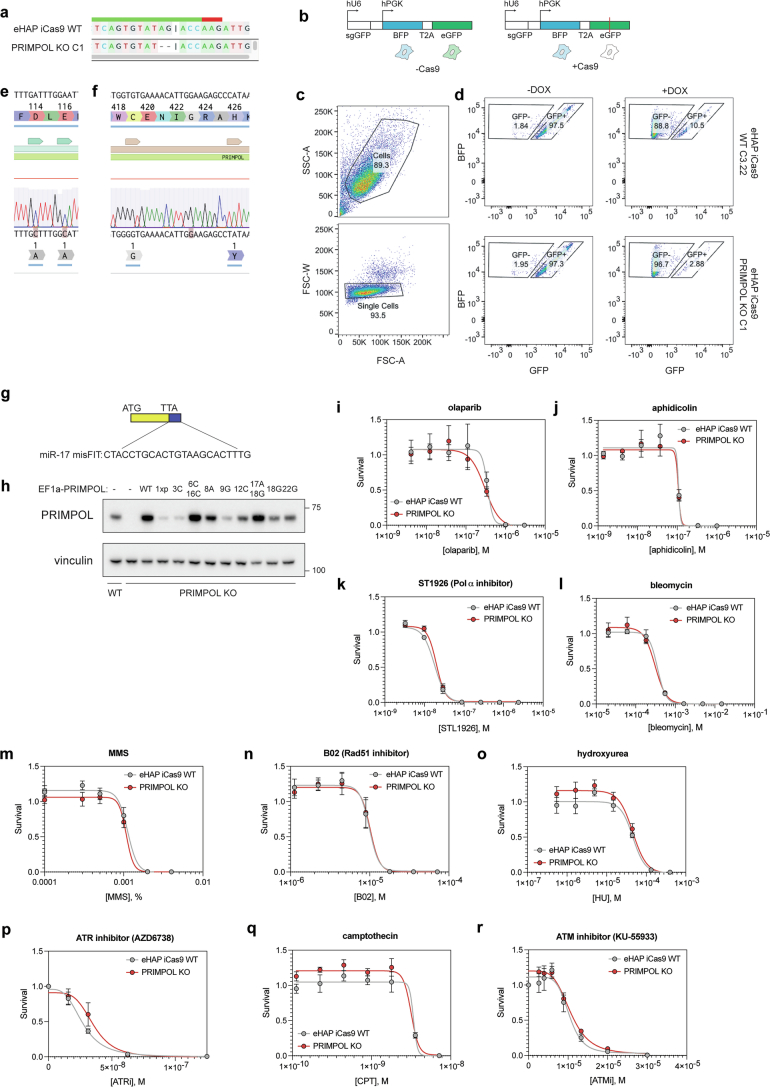
Validation of eHAP iCas9 PRIMPOL KO cell lines and drug dose-response curves. (**a**) DECODR analysis of Sanger sequencing of the PRIMPOL PR sgRNA cut site in eHAP iCas9 PRIMPOL KO C1 cells showing the cells habour a -2 bp deletion. (**b**) Experimental scheme for assessing inducible Cas9 activity using a lentiviral BFP/GFP flow cytometry reporter assay. (**c**) Representative gating strategy for all flow cytometry experiments conducted in this study. (**d**) Flow cytometry of eHAP iCas9 WT or PRIMPOL KO C1 cells infected with the BFP/GFP reporter shown in (B) in the presence or absence of doxycycline. (**e**) Sanger sequencing of the PRIMPOL AxA mutant (D114A/E116A) cDNA. (**f**) Sanger sequencing of the PRIMPOL CH mutant (C419H/H426Y) cDNA. (**g**) Scheme for introducing microRNA response elements (MREs) into the 3’ UTRs of PRIMPOL expression constructs to fine-tune protein expression levels. (**h**) A Western blot showing PRIMPOL expression levels in eHAP iCas9 WT (lane 1) or PRIMPOL KO cells transiently transfected with PRIMPOL expression constructs containing indicated MREs. (**i**) Dose response curves for eHAP iCas9 WT or PRIMPOL KO cells challenged with indicated concentrations of the PARP inhibitor Olaparib. (**j**) Dose response curves for eHAP iCas9 WT or PRIMPOL KO cells challenged with indicated concentrations of the DNA polymerase inhibitor aphidicolin. (**k**) Dose response curves for eHAP iCas9 WT or PRIMPOL KO cells challenged with indicated concentrations of the DNA polymerase alpha inhibitor ST1926. (**l**) Dose response curves for eHAP iCas9 WT or PRIMPOL KO cells challenged with indicated concentrations of bleomycin. (**m**) Dose response curves for eHAP iCas9 WT or PRIMPOL KO cells challenged with indicated concentrations of the methyl methane sulfonate (MMS). (**n**) Dose response curves for eHAP iCas9 WT or PRIMPOL KO cells challenged with indicated concentrations of the RAD51 inhibitor B02. (**o**) Dose response curves for eHAP iCas9 WT or PRIMPOL KO cells challenged with indicated concentrations of the ribonucleotide reductase inhibitor hydroxyurea (HU). (**p**) Dose response curves for eHAP iCas9 WT or PRIMPOL KO cells challenged with indicated concentrations of the ATR inhibitor AZD6738. (**q**) Dose response curves for eHAP iCas9 WT or PRIMPOL KO cells challenged with indicated concentrations of the topoisomerase I inhibitor camptothecin (CPT). (**r**) Dose response curves for eHAP iCas9 WT or PRIMPOL KO cells challenged with indicated concentrations of ATM inhibitor KU-5593. All experiments were performed in N = 3 biological replicates. Data are represented as means ± SD. Source numerical data and unprocessed blots are available in the source data. Source data

Comparing the dropout hits in our screen with those from previous screens in the RPE-1 p53 KO cell line^33^, we obtained three unique hits: (1) PrimPol, (2) the E2 ubiquitin ligase UBE2A and (3) the E2 ubiquitin ligase UBE2N (Fig. 1c). As UBE2A and UBE2N have well-established roles in protecting against cisplatin cytotoxicity^51,52^, we focused on understanding why loss of PrimPol confers cisplatin sensitivity specifically in eHAP cells. To this end, we generated an isogenic PrimPol KO clone in the eHAP iCas9 cell line, which retained doxycycline-induced Cas9 cutting as measured by a flow cytometry-based assay (Fig. 1d and Extended Data Fig. 1a–d). We validated our screen results by challenging eHAP WT and PrimPol KO cells with cisplatin in a growth curve experiment. PrimPol KO cells exhibited a marked loss in cell fitness when challenged with cisplatin compared with WT cells (Fig. 1e).

To ensure that this phenotype was specific to loss of PrimPol and not due to off-target effects from gene editing or clonal isolation, we complemented PrimPol KO cells with complementary DNAs expressing eGFP, WT PrimPol, catalytically dead PrimPol (AxA: Asp114Ala, Glu116Ala) or primase-dead PrimPol (CH: Cys419Gly, His426Tyr) (Extended Data Fig. 1e,f). To avoid artifacts associated with protein overexpression, we utilized the microRNA silencing-mediated fine-tuners (misFIT) system^53^ to decrease cellular expression of all cDNA constructs (Extended Data Fig. 1g,h). We selected a misFIT sequence that lowered WT PrimPol expression levels to near-endogenous levels (Extended Data Fig. 1h; 12C mutant). These constructs were stably expressed in the eHAP iCas9 PrimPol KO cell line and subsequently challenged with cisplatin (Fig. 1f,g). Only the WT PrimPol construct was able to complement cisplatin sensitivity observed in the PrimPol KO cell line, indicating that this phenotype is specific to loss of PrimPol (Fig. 1h). These data suggest that the repriming activity of PrimPol is required to tolerate cisplatin-induced DNA damage.

Next, we sought to understand whether PrimPol activity protects against other types of DNA damage in human cells. Previous reports have suggested that loss of PrimPol confers sensitivity to the bulky base adduct BPDE and interstrand crosslinking agent MMC^21,25,33^. We challenged both eHAP iCas9 WT and PrimPol KO cells with BPDE, MMC and ten other genotoxins or inhibitors of DDR proteins (Fig. 1i and Extended Data Fig. 1i–r). As previously reported, loss of PrimPol conferred cellular toxicity to both BPDE and MMC (Fig. 1j,k). However, no robust phenotype was observed when PrimPol KO cells were challenged with other types of DNA damage or inhibitors of DDR proteins (Fig. 1l and Extended Data Fig. 1i–r). Thus, loss of PrimPol-mediated repriming specifically confers cellular sensitivity to bulky base adducts induced by cisplatin, BPDE and MMC.

### Targeted CRISPR–Cas9 screens identify *SLFN11* and *USP1* as drivers of cisplatin sensitivity in PrimPol KO cells

**Fig. 2:**
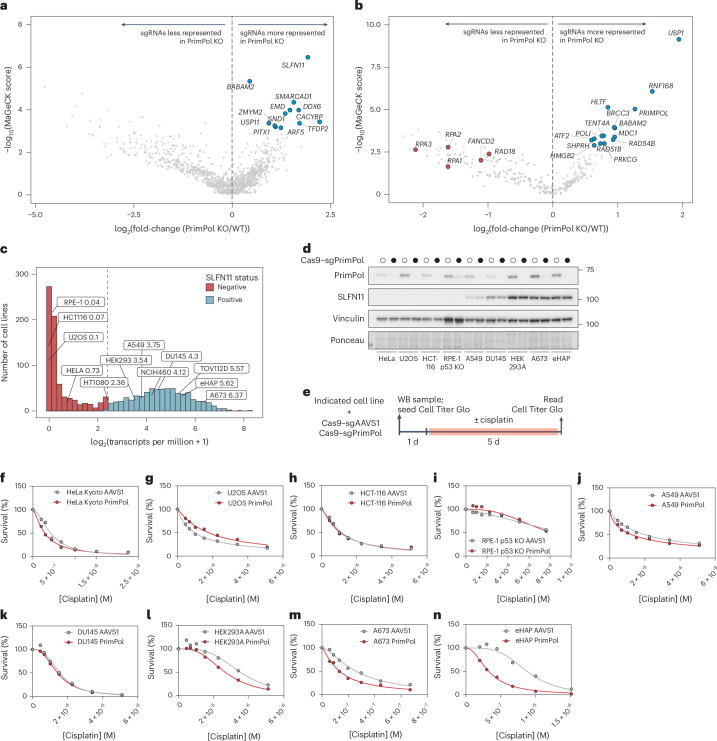
Targeted CRISPR–Cas9 screens identify SLFN11 and USP1 as candidate drivers of cisplatin sensitivity in PrimPol KO cells. **a**, A volcano plot measuring statistical significance against log_2_(fold-change) in sgRNA counts in eHAP iCas9 PrimPol KO cells versus WT cells in the cisplatin-treated arm of the pool 1 sgRNA screen on day 6. Labelled genes exhibited increased sgRNA counts in PrimPol KO cells versus WT cells. **b**, A volcano plot measuring statistical significance against log_2_(fold-change) in sgRNA counts in eHAP iCas9 PrimPol KO cells versus WT cells in the cisplatin-treated arm of the pool 2 sgRNA screen on day 6. Genes labelled in blue exhibited increased sgRNA counts whereas genes labelled in red exhibited decreased sgRNA counts in PrimPol KO cells versus WT cells. **c**, A histogram of curated messenger RNA sequencing data depicting the expression of SLFN11 in human cancer cell lines curated from the DepMap repository. **d**, A western blot (WB) showing SLFN11 and PrimPol expression levels in HeLa, U2OS, HCT116, A549, HEK293A, DU145, eHAP and A673 cells infected with lentiviruses harbouring either Cas9–AAVS1 or Cas9–PrimPol. **e**, Experimental scheme showing how HeLa, U2OS, HCT116, A549, HEK293A, DU145, eHAP and A673 cells infected with Cas9–sgAAVS1 or Cas9–sgPrimPol were challenged with cisplatin and cell viability measured using the Cell Titer Glo assay. **f**–**n**, Cisplatin dose–response curves for HeLa Kyoto (**f**), U2OS (**g**), HCT116 (**h**), RPE-1 p53 KO (**i**), A549 (**j**), DU145 (**k**), HEK293A (**l**), A673 (**m**) and eHAP (**n**) cell lines harbouring Cas9–AAVS1 or Cas9–PrimPol. All experiments were performed in *n* = 2 biological replicates. Data are presented as means. Source numerical data and unprocessed blots are available in the source data. Source data

To identify drivers of cisplatin sensitivity in cells deficient in PrimPol-mediated repriming, we performed two targeted CRISPR–Cas9 dropout screens as previously described (Fig. 1a). We compared sgRNA counts between PrimPol KO and WT eHAP iCas9 cells in cisplatin-treated conditions, which identified loss of the tRNase SLFN11 as the top hit conferring cisplatin resistance in PrimPol KO cells in the pool 1 screen (Fig. 2a). In addition, we identified loss of the deubiquitinase USP1 as the top hit conferring cisplatin resistance in PrimPol KO cells in the pool 2 screen (Fig. 2b). Our screens also identified loss of FANCD2, RAD18 and the RPA trimeric complex as synthetic lethal with loss of PrimPol in cisplatin-treated cells (Fig. 2b).

We first sought to characterize the relationship between SLFN11 and PrimPol-mediated repriming in response to cisplatin treatment. Previous work identified SLFN11 activity as a potent sensitizer to a wide range of chemotherapies, including cisplatin^36,42^. Importantly, over half of cell lines catalogued in the DepMap repository do not express SLFN11^44^, including widely used non-cancerous RPE-1 and cancerous HeLa, U2OS and HCT116 (Fig. 2c). To explore whether loss of SLFN11 expression masks cisplatin sensitivity in PrimPol KO cells, we introduced a Cas9–sgRNA cassette targeting the *AAVS1* safe harbour site or *PRIMPOL* in nine cell lines with varied tissue origin and differing PrimPol and SLFN11 expression levels (Fig. 2d).

**Extended Data Fig. 2:**
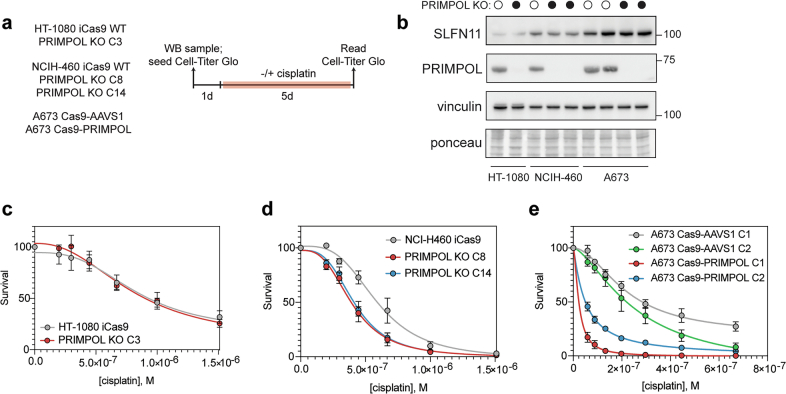
Loss of PRIMPOL confers cisplatin sensitivity differentially in cell lines expressing different amounts of SLFN11. (**a**) An experimental scheme demonstrating how viability assays were performed in indicated PRIMPOL KO cell lines. (**b**) A Western blot depicting PRIMPOL and SLFN11 protein levels in indicated cell lines and PRIMPOL KO clones. (**c**) A cisplatin dose response curve for HT-1080 ICas9 WT or PRIMPOL KO cells. Experiments were performed in N = 2 biological replicates. Data are represented as means ± SD. (**d**) Cisplatin dose response curves for NCIH-460 iCas9 WT or PRIMPOL KO clones. Experiments were performed in N = 3 biological replicates. Data are represented as means ± SD. (**e**) A cisplatin dose response curve for A673 Cas9-AAVS1 or A673 Cas9-PRIMPOL KO clones. These experiments were performed in N = 3 biological replicates. Data are represented as means ± SD. Source numerical data and unprocessed blots are available in the source data. Source data

Loss of PrimPol in cell lines expressing no or low levels of SLFN11 did not confer cisplatin sensitivity (Fig. 2e–k). Conversely, loss of PrimPol in the three cell lines expressing high levels of SLFN11—HEK293A, eHAP and A673—conferred mild-to-severe cisplatin sensitivity (Fig. 2l–n). Importantly, we also generated PrimPol KO clones in cell lines expressing low (HT-1080), medium (NCIH-460) or high (A673) levels of SLFN11. Loss of PrimPol did not confer cisplatin sensitivity in the HT-1080 cell line (Extended Data Fig. 2a–c) but did confer moderate-to-severe cisplatin sensitivity in NCIH-460 and A673 cell lines (Extended Data Fig. 2d,e). Together, these data suggest a correlation between SLFN11 expression levels and cisplatin sensitivity when PrimPol-mediated repriming is inactivated.

### The SLFN11–GCN2 axis drives apoptotic cell death through recognition of cisplatin-induced ssDNA in PrimPol KO cells

**Fig. 3:**
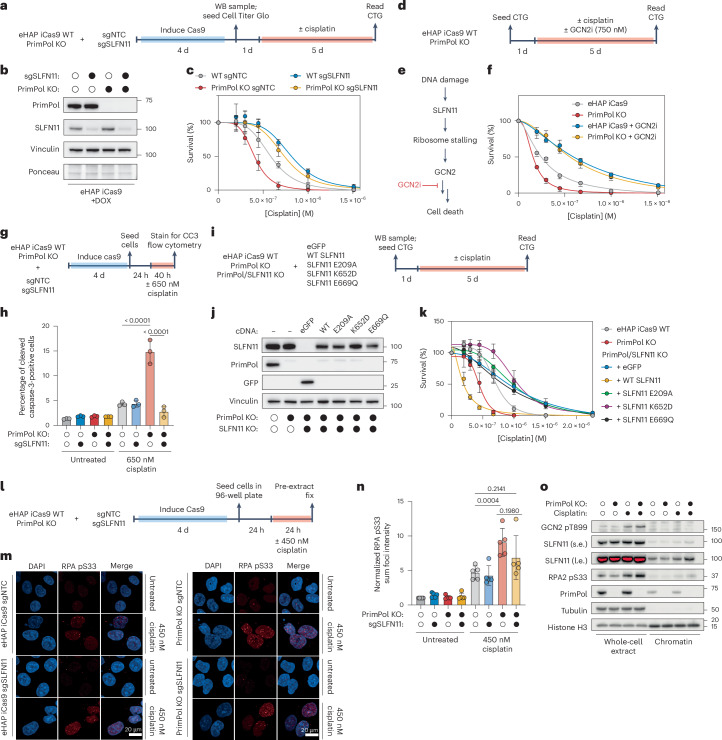
The SLFN11–GCN2 axis drives apoptotic cell death through recognition of cisplatin-induced ssDNA in PrimPol KO cells. **a**, A schematic depicting how viability assays were performed. CTG, Cell Titer Glo. **b**, A western blot demonstrating transient knockout of SLFN11 in eHAP iCas9 WT or PrimPol KO cells. **c**, A dose–response curve of eHAP iCas9 WT or PrimPol KO cells in response to the indicated doses of cisplatin on loss of SLFN11. These experiments were performed in *n* = 3 biological replicates. Data are presented as mean ± s.d. These experiments were performed using the same WT sgNTC and PrimPol KO sgNTC samples depicted in Fig. 5d. **d**, An experimental scheme for challenging eHAP iCas9 WT or PrimPol KO cells with cisplatin in the presence or absence of A-92, a chemical inhibitor of GCN2. **e**, A schematic depicting the signalling cascade connecting DNA damage to the ISR factor GCN2 and subsequent ribosome stalling and cell death. Inhibition of GCN2 prevents ribosome stalling and downstream cell death. **f**, Cisplatin dose–response curves for eHAP iCas9 WT or PrimPol KO cells in the presence or absence of 750 nM GCN2 inhibitor A-92. Experiments were performed in *n* = 3 biological replicates. Data are presented as mean ± s.d. **g**, An experimental scheme for measuring apoptosis via cleaved caspase-3 using flow cytometry. **h**, A bar plot depicting the percentage of cleaved caspase-3 positive cells determined using flow cytometry as shown in **g**. Experiments were performed in *n* = 3 biological replicates. Data are presented as mean ± s.d. A two-way ANOVA was performed to determine biological significance. *P* values: <0.0001 (WT versus PrimPol KO sgNTC + cisplatin); <0.0001 (PrimPol KO sgNTC versus PrimPol KO sgSLFN11 + cisplatin). **i**, An experimental scheme for measuring cell viability in response to cisplatin. **j**, A western blot showing SLFN11, PrimPol and GFP protein expression in SLFN11 WT, SLFN11 E209A (tRNA nuclease mutant), SLFN11 Lys652Asp (ssDNA-binding mutant) or SLFN11 Glu669Gln (helicase mutant) re-expression cell lines. **k**, Dose–response curves for SLFN11 re-expression cell lines challenged with cisplatin. These experiments were performed in *n* = 3 biological replicates. Data are presented as mean ± s.d. **l**, An experimental setup depicting how chromatin-fractionation immunofluorescence experiments were performed. **m**, The eHAP iCas9 WT or PrimPol KO cells transiently depleted of SLFN11 mock treated or treated with 450 nM cisplatin for 24 h. Representative micrographs of chromatin-bound immunofluorescence of phospho-RPA32 Ser33, DAPI or merged are shown. **n**, Quantification of sum focal intensity of phospho-RPA32 Ser33 in the indicated cell lines. Data were normalized in each biological replicate to untreated WT samples. These experiments were performed in *n* = 5 biological replicates. Data are presented as mean ± s.d. A two-way analysis of variance (ANOVA) was performed to assess biological significance. *P* values: 0.0004 (WT versus PrimPol KO sgNTC + cisplatin); 0.2141 (WT sgNTC versus PrimPol KO sgSLFN11 + cisplatin); 0.1980 (PrimPol KO sgNTC versus PrimPol KO sgSLFN11 + cisplatin). The pRPA S33 staining was performed as a co-stain with γH2AX phospho-Ser139 depicted in Extended Data Fig. 4f–h. **o**, A representative western blot depicting levels of RPA phospho-Ser33, SLFN11 and GCN2 phospho-Thr899 in whole-cell extracts and chromatin fractions after treatment with 1 μM cisplatin for 24 h. This blot was performed in *n* = 2 biological replicates. Source numerical data and unprocessed blots are available in the source data. s.e., short exposure; l.e., long exposure. Source data

**Extended Data Fig. 3:**
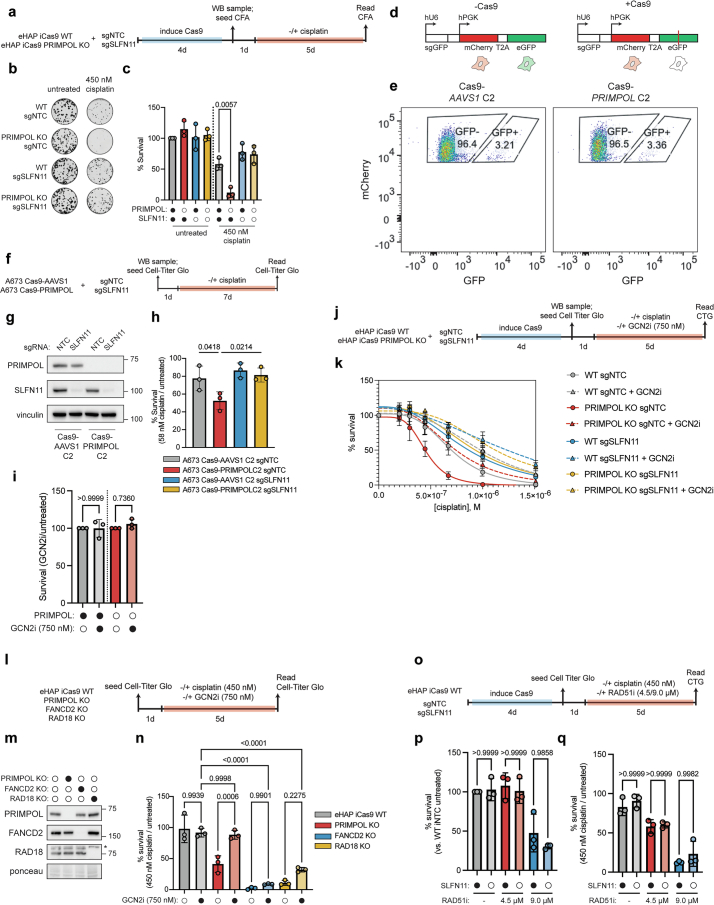
Cisplatin treatment of PRIMPOL KO cells leads to a SLFN11- and GCN2-dependent cell death. (**a**) A schematic depicting how viability assays were performed. (**b**) Representative images of colony formation assays used to assess viability of eHAP iCas9 WT or PRIMPOL KO cells in response to cisplatin upon loss of SLFN11. (**c**) Quantitation of colony formation assays as depicted in (b). These experiments were performed in N = 3 biological replicates. Data are represented as means ± SD. A two-way ANOVA was used to calculate biological significance. *P*-values: 0.0057 (WT sgNTC vs. PRIMPOL KO sgNTC + cisplatin). (**d**) Experimental scheme for a lentiviral-based mCherry/GFP Cas9 flow cytometry reporter. (**e**) Flow cytometry of A673 Cas9-AAVS1 C2 or A673 Cas9-PRIMPOL C2 cells showing Cas9 activity in these cells. (**f**) Experimental scheme for challenging A673 Cas9-AAVS1 C2 or A673 Cas9-PRIMPOL C2 cells transiently depleted of SLFN11 protein with cisplatin. (**g**) A Western blot showing PRIMPOL and SLFN11 protein levels in A673 Cas9-AAVS1 C2 and A673 Cas9-PRIMPOL cells transiently depleted of SLFN11 protein. (**h**) Bar blots showing cell survival of A673 Cas9-AAVS1-C2 or A673 Cas9-PRIMPOL C2 cells transiently depleted of SLFN11 protein in the presence of 58 nM cisplatin. Experiments were performed in N = 3 biological replicates. Data are represented as means ± SD. A one-way ANOVA was performed to assess biological significance. *P*-values: 0.0418 (A673 Cas9-AAVS1 sgNTC vs. A673 Cas9-PRIMPOL sgNTC); 0.0214 (A673 Cas9-PRIMPOL sgNTC vs. A673-Cas9 PRIMPOL sgSLFN11). (**i**) Bar plots showing cell survival of eHAP iCas9 WT or PRIMPOL KO cells in response to treatment with 750 nM GCN2 inhibitor in the absence of cisplatin. Experiments were performed in N = 3 biological replicates. Data are represented as means ± SD. A two-way ANOVA was performed to assess biological significance. *P*-values: >0.9999 (WT vs. WT + GCN2i); 0.7360 (PRIMPOL KO vs. PRIMPOL + GCN2i). (**j**) Experimental scheme for challenging eHAP iCas9 WT or PRIMPOL KO cells with cisplatin in the presence or absence of GCN2 inhibitor following transient knockout of SLFN11. (**k**) A dose response curve depicting cells with indicated genotypes challenged with cisplatin in the absence (solid lines, circles) or presence (dashed lines, triangled) of 750 nM GCN2 inhibitor. Experiments were performed in N = 3 biological replicates. Data are represented as means ± SD. (**l**) An experimental scheme for challenging eHAP iCas9 WT, PRIMPOL KO, FANCD2 KO, or RAD18 KO cells with cisplatin in the presence or absence of 750 nM GCN2 inhibitor. (**m**) A Western blot showing PRIMPOL, FANCD2, and RAD18 protein levels in eHAP iCas9 WT, PRIMPOL KO, FANCD2 KO, and RAD18 KO cells. (**n**) A bar plot depicting cell survival of eHAP iCas9 WT, PRIMPOL KO, FANCD2 KO, and RAD18 KO cells at 450 nM cisplatin compared to an untreated control in each cell line in the absence (darker bars) or presence (lighter bars) of GCN2 inhibitor. These experiments were performed in N = 3 biological replicates. Data are represented as means ± SD. A two-way ANOVA was performed to assess statistical significance. *P*-values: 0.9939 (WT vs. WT + GCN2i); 0.0006 (PRIMPOL KO vs. PRIMPOL KO + GCN2i); 0.9901 (FANCD2 KO vs. FANCD2 KO + GCN2i); 0.2275 (RAD18 KO vs. RAD18 KO + GCN2i); 0.9998 (WT + GCN2i vs. PRIMPOL KO + GCN2i); <0.0001 (WT + GCN2i vs. FANCD2 KO + GCN2i); <0.0001 (WT + GCN2i vs. RAD18 KO + GCN2i). (**o**) An experimental scheme for challenging eHAP iCas9 WT cells with cisplatin in the presence or absence of RAD51 inhibitor (B02) following transient knockout of SLFN11. (**p**) A bar plot depicting cell survival in untreated conditions in the absence or presence of RAD51 inhibitor following transient knockout of SLFN11. These experiments were performed in N = 3 biological replicates. Data are represented as means ± SD. A two-way ANOVA was performed to assess statistical significance. *P*-values: >0.9999 (WT sgNTC vs. WT sgSLFN11); >0.9999 (WT sgNTC + 4.5 µM RAD51i vs. WT sgSLFN11 + 4.5 µM RAD51i); 0.9858 (WT sgNTC 9.0 µM RAD51i vs. WT sgSLFN11 + 9.0 µM RAD51i). (**q**) A bar plot depicting cell survival in cells treated with 450 nM cisplatin in the absence or presence of RAD51 inhibitor following transient knockout of SLFN11. These experiments were performed in N = 3 biological replicates. Data are represented as means ± SD. A two-way ANOVA was performed to assess statistical significance. *P*-values: >0.9999 (WT sgNTC vs. WT sgSLFN11); >0.9999 (WT sgNTC + 4.5 µM RAD51i vs. WT sgSLFN11 + 4.5 µM RAD51i); 0.9982 (WT sgNTC 9.0 µM RAD51i vs. WT sgSLFN11 + 9.0 µM RAD51i). Source numerical data and unprocessed blots are available in the source data. Source data

We sought to determine whether SLFN11 directly promotes cisplatin sensitivity on PrimPol inactivation (Fig. 3a). Inducible loss of SLFN11 conferred a growth benefit in both WT and PrimPol KO cells when challenged with cisplatin (Fig. 3b,c). Strikingly, loss of SLFN11 completely rescued cisplatin sensitivity observed in PrimPol KO cells to levels indistinguishable from WT cells (Fig. 3c). These results were recapitulated in an orthogonal colony formation assay (Extended Data Fig. 3a–c) and in the SLFN11-expressing A673 cell line (Extended Data Fig. 3f–h).

A recent landmark study implicated SLFN11 ssDNA-dependent tRNase activity as a trigger for downstream ribosome stalling and GCN2 (gene name *EIF2AK4*) activation^38^. To determine whether SLFN11-dependent activation of GCN2 confers cisplatin cytotoxicity in PrimPol KO cells, we challenged eHAP iCas9 WT or PrimPol KO cells with cisplatin in the presence of a fixed dose of A-92, a specific inhibitor of GCN2 (GCN2i)^54^ (Fig. 3d,e). Inhibition of GCN2 completely rescued cisplatin cytotoxicity in PrimPol KO cells to levels indistinguishable from WT cells (Fig. 3f). Importantly, this fixed dose of GCN2 inhibitor did not affect cell growth in untreated conditions, ruling out any effect on cell-cycle progression (Extended Data Fig. 3i). In addition, we confirmed that SLFN11 and GCN2 act epistatically to confer cisplatin sensitivity in PrimPol KO cells because concurrent loss of SLFN11 and GCN2 inhibition did not confer additive resistance to cisplatin in any genotype (Extended Data Fig. 3j,k).

Next, we examined whether SLFN11–GCN2-dependent cell death selectively confers cisplatin sensitivity in PrimPol KO cells or whether this is a generalized mechanism of cell death when other DDR pathways are compromised. Thus, we generated FANCD2 and RAD18 KO clones in eHAP iCas9 cells, then challenged these cell lines with cisplatin in the presence or absence of a fixed dose of GCN2i (Extended Data Fig. 3l,m). Inhibition of GCN2 completely rescued cisplatin sensitivity in PrimPol KO cells but did not provide any significant growth benefit in FANCD2 or RAD18 KO cells (Extended Data Fig. 3n). Similarly, loss of SLFN11 did not rescue cisplatin sensitivity when HR was inhibited using B02, a RAD51 inhibitor^55^ (Extended Data Fig. 3o–q). Together, these data suggest that the SLFN11–GCN2 axis selectively confers cisplatin sensitivity when repriming is inactivated.

**Extended Data Fig. 4:**
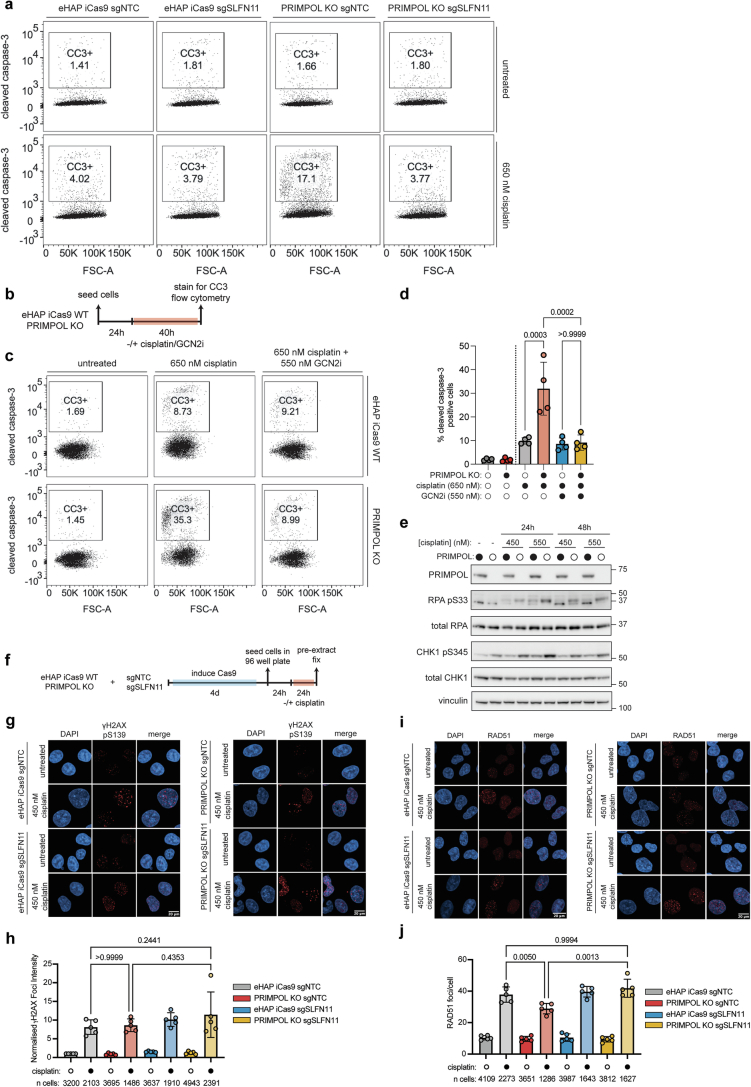
Cisplatin induces ssDNA accumulation, SLFN11 chromatin loading, induction of the ISR, and apoptosis in the absence of PRIMPOL-mediated repriming. (**a**) Representative flow cytometry experiments depicting cleaved caspase-3 signal plotted against forward scatter area. Cleaved caspase-3 positive cells were gated and quantitated as depicted. (**b**) A scheme depicting how apoptosis was measured in eHAP iCas9 WT or PRIMPOL KO cells using cleaved caspase-3 positive cells in flow cyomtetry experiments. (**c**) Representative dot plots depicting how the percentage of cleaved caspase-3 positive cells were determined using flow cytometry after challenge with cisplatin in the absence or presence of 650 nM GCN2 inhibitor. (**d**) A bar plot depicting the percentage of cleaved caspase-3 positive cells in each indicated genotype following challenge with cisplatin in the absence or presence of GCN2 inhibitor determined using flow cytometry in (b). These experiments were performed in N = 4 biological replicates. Data represented as means ± SD. A two-way ANOVA was performed to assess biological significance *P*-values: 0.0003 (WT sgNTC vs. PRIMPOL KO sgNTC + cisplatin); >0.9999 (WT sgSLFN11 vs. PRIMPOL KO sgSLFN11 + cisplatin); 0.0002 (PRIMPOL KO sgNTC vs. PRIMPOL KO sgSLFN11 + cisplatin). (**e**) Representative Western blots demonstrating induction of DNA damage response markers in eHAP iCas9 WT or PRIMPOL KO cells in response to indicated doses of cisplatin for 24 or 48 h. (**f**) Experimental setup of measuring chromatin-bound phospho-gamma H2AX serine 139 using immunofluorescence in eHAP iCas9 WT or PRIMPOL KO cells depleted of SLFN11 protein (top). (**g**) eHAP iCas9 WT or PRIMPOL KO Cells transiently depleted of SLFN11 were mock treated or treated with 450 nM cisplatin for 24 h. Representative micrographs of chromatin-bound immunofluorescence of γH2AX phospho serine 139, DAPI, or merged are shown. (**h**) Bar plots showing phospho-gamma H2AX serine 139 foci signal normalised to WT untreated cells in eHAP iCas9 WT or PRIMPOL KO cells depleted of SLFN11 protein. Experiments were performed in N = 5 biological replicates. Data are represented as means ± SD. A two-way ANOVA was used to assess biological significance. *P*-values: >0.9999 (WT sgNTC vs. PRIMPOL KO sgNTC + cisplatin); 0.4353 (PRIMPOL KO sgNTC vs. PRIMPOL KO sgSLFN11 + cisplatin); 0.2441 (WT sgNTC vs. PRIMPOL KO sgSLFN11). γH2AX phospho serine 139 staining was performed as a co-stain with pRPA S33 depicted in Fig. 4j-l. (**i**) eHAP iCas9 WT or PRIMPOL KO Cells transiently depleted of SLFN11 were mock treated or treated with 450 nM cisplatin for 24 h. Representative micrographs of chromatin-bound immunofluorescence of RAD51, DAPI, or merged are shown (**j**) Quantification of RAD51 foci in indicated cell lines. These experiments were performed in N = 5 biological replicates. Data are represented as means ± SD. A two-way ANOVA was performed to assess biological significance. *P*-values: 0.0050 (WT sgNTC vs. PRIMPOL KO sgNTC + cisplatin); 0.0013 (PRIMPOL KO sgNTC vs. PRIMPOL KO sgSLFN11 + cisplatin); 0.9994 (WT sgNTC vs. PRIMPOL KO sgSLFN11 + cisplatin). Source numerical data and unprocessed blots are available in the source data. Source data

Previous studies have determined that SLFN11 triggers p53-independent apoptosis in response to DNA damage, either through activation of the ribotoxic stress response or downregulation of the anti-apoptotic factor MCL1^38,39^. We sought to determine whether the SLFN11–GCN2-dependent cell death that we observed was due to apoptosis (Fig. 3g). Cisplatin treatment modestly induced apoptosis in WT cells in an SLFN11-independent manner (Fig. 3h and Extended Data Fig. 4a). In the absence of PrimPol, cisplatin robustly induced apoptosis that was completely dependent on SLFN11 and GCN2 (Fig. 3h and Extended Data Fig. 4b–d). Together, these data demonstrate that cisplatin induces SLFN11-dependent and GCN2-dependent apoptotic cell death when PrimPol-mediated repriming is inactivated.

The ssDNA-binding, tRNA nuclease and helicase activities of SLFN11 have all been implicated as sensitizing cells to genotoxic stress^37–39^. To determine which functions of SLFN11 were required to confer cisplatin sensitivity in PrimPol KO cells, we generated an eHAP iCas9 PrimPol–SLFN11 double KO clone and re-expressed eGFP, WT SLFN11, SLFN11 Glu209Ala (tRNA nuclease mutant), SLFN11 Lys652Asp (ssDNA-binding mutant) or SLFN11 Glu669Gln (Walker B–helicase mutant) proteins to near-endogenous levels (Fig. 3i,j). All three activities of SLFN11 were required to confer cisplatin sensitivity in PrimPol KO cells (Fig. 3k).

As ssDNA binding was required to induce SLFN11-dependent cell death, we hypothesized that ssDNA accumulation at cisplatin-stalled replication forks could trigger SLFN11 activation. Levels of replication checkpoint activation and fork-associated ssDNA accumulation were determined by staining for ATR-dependent phosphorylation of RPA2 at Ser33 (RPA pSer33) in response to cisplatin (Fig. 3l). We observed a twofold increase in the mean focal intensity of RPA pSer33 in PrimPol KO cells treated with cisplatin compared with WT cells (Fig. 3m,n). This robust activation of the replication checkpoint was not completely suppressed on deletion of SLFN11 (Fig. 3m,n). We observed a similar induction of RPA pSer33 using western blotting techniques (Extended Data Fig. 4e). Neither γH2AX–pSer139 nor RAD51 levels increased after low doses of cisplatin treatment in PrimPol KO cells when compared with WT cells (Extended Data Fig. 4g–j). Finally, we observed a robust accumulation of SLFN11 on chromatin and activation of the ISR (as measured by phosphorylation of GCN2 at Thr899) after cisplatin treatment, particularly in PrimPol KO cells (Fig. 3o). Together, these data suggest that ssDNA accumulates at cisplatin-stalled replication forks in the absence of repriming, which could serve as a lesion for SLFN11 and GCN2 activation.

### RPA exhaustion activates SLFN11–GCN2-dependent cell death

Having established a correlation between cisplatin-induced ssDNA accumulation at stalled forks and the induction of SLFN11-dependent cell death in PrimPol KO cells (Fig. 3k,l and Extended Data Fig. 4e), we sought to understand how SLFN11 is activated in response to these lesions. Our screens revealed that cells lacking repriming are particularly sensitive to loss of the RPA heterotrimeric complex when challenged with cisplatin (Fig. 2b). Under heightened replication stress, cells can undergo RPA exhaustion, where the available levels of RPA in the nucleus are not sufficient for the ssDNA generated in the cell^9^. We hypothesized that RPA exhaustion could result in uncoated ssDNA, which could serve as a template for SLFN11 binding to stalled forks, activating its tRNA nuclease activity and downstream apoptosis.

**Fig. 4:**
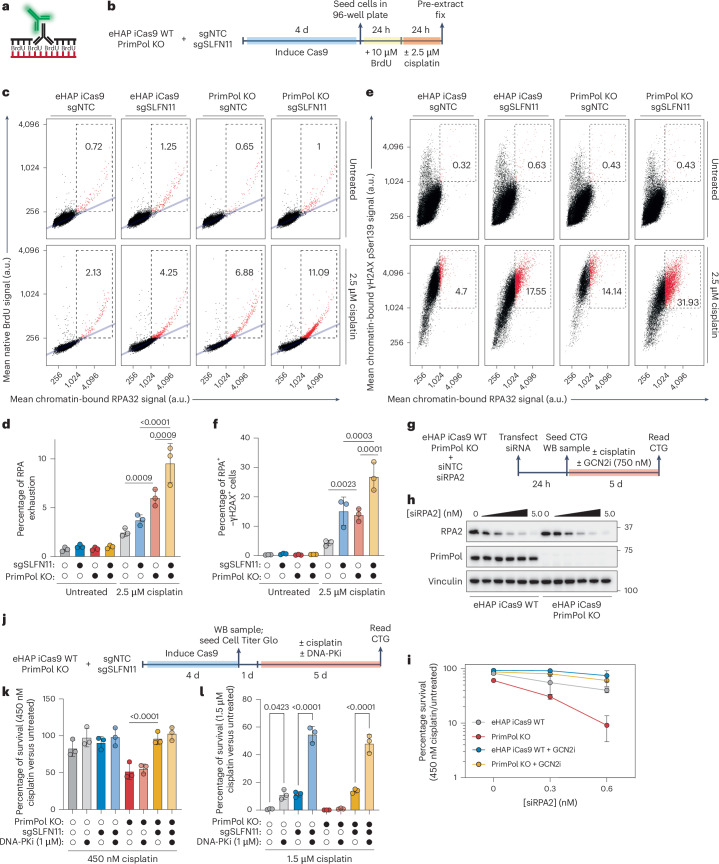
RPA exhaustion activates SLFN11–GCN2-dependent cell death. **a**, A diagram depicting how ssDNA was measured by detecting incorporated BrdU under non-denaturing conditions. **b**, An experimental scheme for measuring RPA exhaustion in eHAP iCas9 WT or PrimPol KO cells in which SLFN11 has been transiently knocked out using QIBC. **c**, Representative scatter plots depicting mean BrdU signal intensity per cell (*y* axis) plotted against mean chromatin-bound RPA32 (*x* axis). The linear relationship between BrdU and RPA32 signals is depicted as a light-blue line. Cells undergoing RPA exhaustion are depicted within the dashed box and coloured red. The percentage of cells undergoing RPA exhaustion is indicated in each panel. **d**, A bar plot depicting the percentage of cells undergoing RPA exhaustion in the experiments shown in **a** and **b**. These experiments were performed in *n* = 3 biological replicates. Data are presented as mean ± s.d. A two-way ANOVA was performed to assess biological significance. *P* values: 0.0009 (WT sgNTC versus PrimPol KO sgNTC + cisplatin); <0.0001 (WT sgSLFN11 versus PrimPol KO sgSLFN11 + cisplatin); and 0.0009 (PrimPol KO sgNTC versus PrimPol KO sgSLFN11 + cisplatin). **e**, Representative scatter plots depicting mean γH2AX–pSer139 signal intensity per cell (*y* axis) plotted against mean chromatin-bound RPA32 (*x* axis). Cells with high RPA32 and γH2AX–pSer139 signals are coloured in red. The percentage of cells with high RPA32 and γH2AX–pSer139 signals is indicated in each panel. **f**, A bar plot depicting the percentage of cells exhibiting high RPA32–γH2AX–pSer139 signals in the experiments shown in **a** and **e**. These experiments were performed in *n* = 3 biological replicates. Data are presented as mean ± s.d. A two-way ANOVA was performed to assess biological significance. *P* values: 0.0023 (WT sgNTC versus PrimPol KO sgNTC + cisplatin); 0.0003 (WT sgSLFN11 versus PrimPol KO sgSLFN11 + cisplatin); and 0.0001 (PrimPol KO sgNTC versus PrimPol KO sgSLFN11 + cisplatin). **g**, Experimental scheme for challenging eHAP iCas9 WT or PrimPol KO cells with cisplatin in the presence or absence of GCN2i after siRNA knockdown of RPA2. **h**, A western blot depicting RPA2, PrimPol, vinculin and total protein levels in eHAP iCas9 WT or PrimPol KO cells after transient knockdown of RPA2. **i**, A dose–response curve depicting cell survival in eHAP iCas9 WT or PrimPol KO cells (normalized to untransfected cells) after siRNA knockdown of RPA at indicated concentrations in the presence or absence of cisplatin and GCN2i. This experiment was performed in *n* = 3 biological replicates. Data are presented mean ± s.d. **j**, An experimental scheme for challenging eHAP iCas9 WT or PrimPol KO cells with two fixed doses of cisplatin in the presence or absence of DNA-PK inhibitor (DNA-PKi) after transient knockout of SLFN11. **k**, A bar plot depicting cell survival in the presence of 450 nM cisplatin with or without treatment with DNA-PKi. The experiments were performed in *n* = 3 biological replicates. Data are presented as mean ± s.d. A two-way ANOVA was performed to assess statistical significance. *P* value: <0.0001 (PrimPol KO sgNTC versus PrimPol KO sgSLFN11 + cisplatin). **l**, A bar plot depicting cell survival in the presence of 1.5 μM cisplatin with or without treatment with DNA-PKi. The experiments shown were performed in *n* = 3 biological replicates. Data are presented as mean ± s.d. A two-way ANOVA was performed to assess statistical significance. *P* values: 0.0423 (WT sgNTC − DNA-PKi versus WT sgNTC + DNA-PKi); <0.0001 (WT sgSLFN11 − DNA-PKi versus WT sgSLFN11 + DNA-PKi); <0.0001 (PrimPol KO sgSLFN11 − DNA-PKi versus PrimPol sgSLFN11 + DNA-PKi). Source data

**Extended Data Fig. 5:**
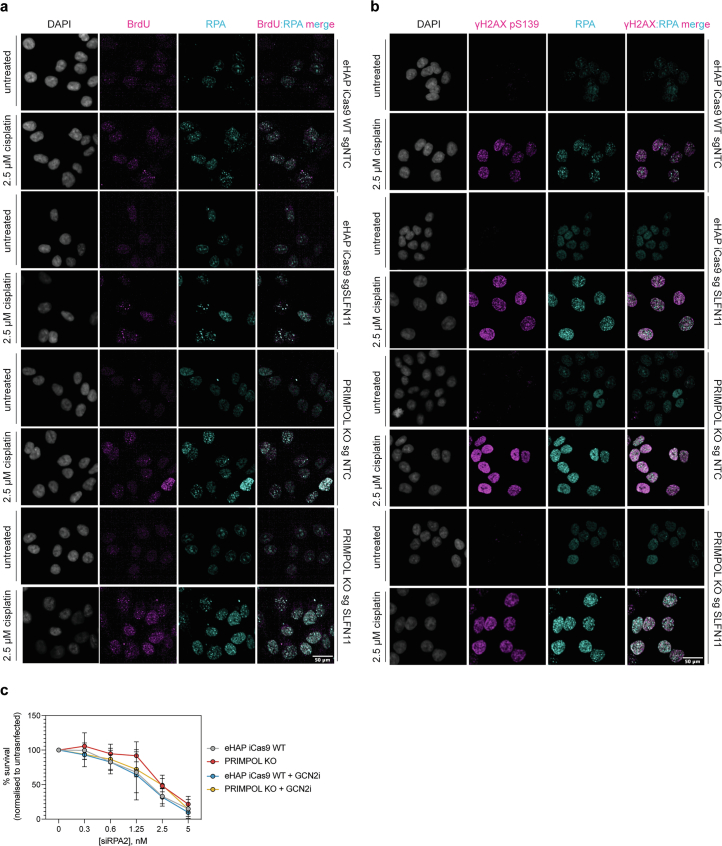
RPA exhaustion induces SLFN11-GCN2-dependent cell death when repriming is inactivated. (**a**) Representative micrographs depicting BrdU, RPA, and DAPI signals generated in QIBC experiments shown in Fig. 4c. (**b**) Representative micrographs depicting γH2AX pS139, RPA, and DAPI signals generated in QIBC experiments shown in Fig. 4e. (**c**) A dose response curve depicting cell viability in eHAP iCas9 WT or PRIMPOL KO cells treated with indicated doses of siRNA targeting the RPA2 gene in the presence or absence of 750 nM GCN2 inhibitor. These experiments were performed in N = 3 biological replicates. Data are represented as means ± SD. Source numerical data are available in the source data. Source data

To determine whether cisplatin induces RPA exhaustion, we utilized quantitative image-based cytometry (QIBC) to simultaneously measure the levels of ssDNA (native 5-bromo-2ʹ-deoxyuridine (BrdU)) and chromatin-bound RPA at a cellular level, as previously described^9^ (Fig. 4a,b and Extended Data Fig. 5a). Cells undergoing RPA exhaustion were quantified by determining those cells where the ratio of ssDNA to chromatin-bound RPA signal deviated from linearity (Fig. 4c). In untreated conditions, <1.5% of cells were undergoing RPA exhaustion, with no significant differences in each genotype tested (Fig. 4c,d). On challenge with 2.5 μM cisplatin, the percentage of cells undergoing RPA exhaustion increased twofold in WT cells. Strikingly, the percentage of cells undergoing RPA exhaustion increased more than tenfold in cells deficient in PrimPol when compared with untreated conditions (Fig. 4c,d). In addition, knockout of SLFN11 resulted in a twofold increase in cells undergoing RPA exhaustion in both WT and PrimPol KO cells (Fig. 4c,d). Together, these data suggest that cells deficient in repriming are more prone to RPA exhaustion in response to cisplatin.

One consequence of RPA exhaustion is the generation of double-stranded breaks (DSBs) that manifest as high γH2AX–pSer139 signal in cells with a high burden of chromatin-bound RPA^9^. We repeated our QIBC experiments and stained for γH2AX–pSer139 and chromatin-bound RPA (Extended Data Fig. 5b). In untreated conditions, <1% of cells contained high γH2AX–pSer139 and chromatin-bound RPA signals (Fig. 4e,f). Although cisplatin treatment modestly increased the percentage of WT cells with high γH2AX–RPA signal, loss of SLFN11 resulted in a robust accumulation of cells with high γH2AX–RPA signal in both WT and PrimPol KO cells (Fig. 4e,f). Although SLFN11-proficient, PrimPol-deficient cells also exhibited a higher percentage of γH2AX–RPA double-positive cells, this is probably due to apoptosis^56^, because these cells are the most sensitive to cisplatin treatment (Fig. 4e). These data suggest that, although RPA exhaustion can occur in both SLFN11-proficient and SLFN11-deficient cells, the latter undergo replication catastrophe and DSB formation at higher rates when compared with the former.

Having established that cisplatin-induced RPA exhaustion occurs more readily when PrimPol is inactivated, we sought to understand whether RPA exhaustion could directly activate SLFN11–GCN2-dependent cell death. To test this possibility, we reduced RPA pools in cells by performing a titration of small interfering (si)RNA targeting the *RPA2* gene in eHAP iCas9 WT or PrimPol KO cells (Fig. 4g,h). In the absence of cisplatin, siRNA-mediated depletion of RPA2 resulted in a dose-dependent loss in cell viability that was independent of SLFN11–GCN2 activity (Extended Data Fig. 5c). Strikingly, PrimPol KO cells exhibited an siRNA dose-dependent sensitization to cisplatin, which was completely rescued on inhibition of GCN2 (Fig. 4i). These results also explain why higher levels of RPA exhaustion are observed in SLFN11-negative cells, probably due to these cells no longer undergoing RPA exhaustion-induced apoptosis (Fig. 4c,d). Together, these data suggest that induced RPA exhaustion confers a SLFN11–GCN2-dependent cisplatin sensitivity when repriming is inactivated.

Having established that SLFN11-deficient cells undergo replication catastrophe and DSB formation at a higher rate after RPA exhaustion than SLFN11-proficient cells, we hypothesized that these DSBs could be suitable substrates for toxic repair via the non-homologous end-joining (NHEJ) repair pathway. To test this hypothesis, we transiently knocked out SLFN11 in eHAP iCas9 WT or PrimPol KO cells and subsequently challenged these cells with cisplatin in the presence or absence of a chemical inhibitor of the catalytic subunit of DNA-dependent protein kinase (DNA-PK), NU7441^57^ (Fig. 4j). At a low dose of cisplatin, PrimPol KO cells exhibited an SLFN11-dependent loss in cell viability that was independent of DNA-PK activity (Fig. 4k). At a high dose of cisplatin, SLFN11-proficient WT or PrimPol KO cells exhibited a SLFN11-independent and DNA-PK-independent sensitization to cisplatin (Fig. 4l). However, in SLFN11-deficient cells, inhibition of DNA-PK resulted in a substantial rescue of cell viability in response to cisplatin, independent of PrimPol status (Fig. 4l). Together, these results suggest that DSBs that form after RPA exhaustion and replication catastrophe in SLFN11-deficient cells are suitable substrates for toxic DNA-PK activity.

### USP1 downregulates the FA pathway to induce RPA exhaustion and activate SLFN11 in cisplatin-treated PrimPol KO cells

Having established SLFN11 as a key determinant of cisplatin sensitivity in PrimPol KO cells, we sought to leverage this phenotype to identify modulators of SLFN11 activation in the absence of repriming. Our targeted CRISPR–Cas9 screens identified loss of the deubiquitinase USP1 as conferring resistance to cisplatin treatment in PrimPol KO cells (Fig. 2b).

**Fig. 5:**
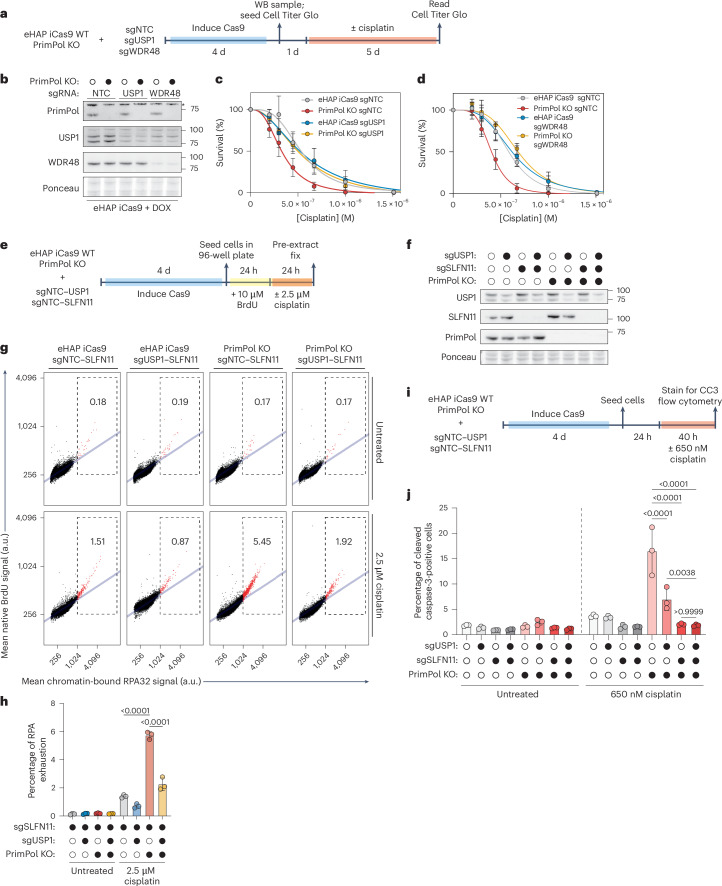
USP1 downregulates the FA pathway to induce RPA exhaustion and activate SLFN11 in cisplatin-treated PrimPol KO cells. **a**, A schematic depicting how cell viability assays were performed in eHAP iCas9 WT or PrimPol KO cells challenged with cisplatin after transient knockout of USP1 or WDR48. **b**, A representative western blot depicting PrimPol, USP1 and WDR48 protein levels on loss of USP1 or WDR48. This western blot was performed in *n* = 2 biological replicates. **c**, A cisplatin dose–response curve for eHAP iCas9 WT or PrimPol cells on loss of USP1. These experiments were performed in *n* = 3 biological replicates. Data are presented as mean ± s.d. **d**, A cisplatin dose–response curve for eHAP iCas9 WT or PrimPol cells on loss of WDR48. These experiments were performed in *n* = 3 biological replicates. Data are presented as mean ± s.d. These experiments were performed using the same WT sgNTC and PrimPol KO sgNTC samples depicted in Fig. 3c. **e**, An experimental scheme for measuring RPA exhaustion in cell pools after transient knockout of USP1 and SLFN11 using QIBC. **f**, A representative western blot showing USP1 and SLFN11 protein levels after transient knockout of each protein. This western blot was performed in *n* = 2 biological replicates. **g**, Representative scatter plots depicting mean ssDNA (BrdU) intensity (*y* axis) plotted against mean chromatin-bound RPA32 intensity (*x* axis) for each genotype and treatment depicted. The dashed boxes indicate RPA-exhausted cells where the ratio of mean ssDNA signal against mean chromatin-bound RPA32 signal has deviated from linearity (blue line). The percentage of cells undergoing RPA exhaustion in each genotype and treatment is shown. **h**, A bar plot depicting the percentage of cells undergoing RPA exhaustion in each genotype and treatment tested. These experiments were performed in *n* = 3 biological replicates. Data are presented as mean ± s.d. A two-way ANOVA was performed to assess biological significance. *P* values: <0.0001 (WT sgSLFN11 versus PrimPol KO sgSLFN11 + cisplatin); and <0.0001 (PrimPol KO sgSLFN11 versus PrimPol KO sgSLFN11–USP1 + cisplatin). **i**, An experimental scheme depicting how apoptosis (cleaved caspase-3 signal) was measured using flow cytometry in eHAP iCas9 cells after transient knockout of USP1 or SLFN11. **j**, A bar plot depicting the percentage of cells undergoing apoptosis, as assessed by measuring the cleaved caspase-3 signal using flow cytometry. These experiments were performed in *n* = 3 biological replicates. Data are presented as mean ± s.d. A two-way ANOVA was performed to assess biological significance. *P* values: <0.0001 (PrimPol KO sgNTC versus PrimPol KO sgUSP1 + cisplatin); <0.0001 (PrimPol KO sgNTC versus PrimPol KO sgSLFN11 + cisplatin); <0.0001 (PrimPol KO sgNTC versus PrimPol KO sgUSP1–SLFN11 + cisplatin); 0.0038 (PrimPol KO sgUSP1 versus PrimPol KO sgSLFN11 + cisplatin); and >0.9999 (PrimPol KO sgSLFN11 versus PrimPol KO sgSLFN11–USP1 + cisplatin). Source numerical data and unprocessed blots are available in the source data. DOX, doxycycline. Source data

**Extended Data Fig. 6:**
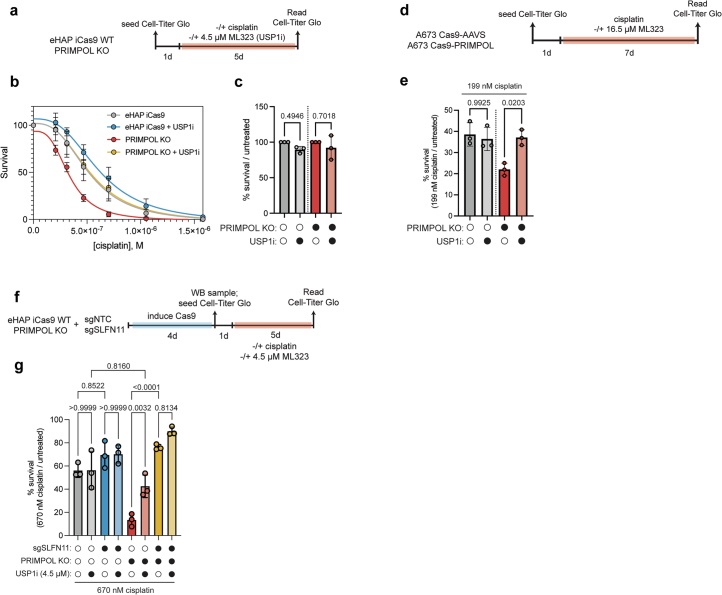
Inhibition of the USP1:WDR48 DUB complex rescues cisplatin-induced SLFN11-dependent cell death in PRIMPOL KO cells. (**a**) An experimental schematic describing how cell viability assays were performed in the presence of cisplatin and ML323 (USP1 inhibitor). (**b**) A cisplatin dose response curve for eHAP iCas9 WT or PRIMPOL KO cells with or without 4.5 μM ML323. These experiments were performed in N = 3 biological replicates. Data are represented as means ± SD. (**c**) A bar plot showing how eHAP iCas9 WT (gray bars) or PRIMPOL KO cells (red bars) respond to 4.5 μM ML323. Survival is normalised to untreated conditions within each genotype. These experiments were performed in N = 3 biological replicates. Data are represented as means ± SD. A two-way ANOVA was performed to assess statistical significance. *P*-values: 0.4946 (WT vs. WT + USP1i); 0.7018 (PRIMPOL KO vs. PRIMPOL KO + USP1i). (**d**) A schematic showing how cell viability experiments were performed in A673 Cas9-AAVS1 or A673 Cas9-PRIMPOL cells in the presence of cisplatin and ML323 (16.5 μM). (**e**) A bar blot depicting cell survival for A673 Cas9-AAVS1 or A673 Cas9-PRIMPOL cells in the presence of 200 nM cisplatin -/+ 16.5 μM ML323. These experiments were performed in N = 3 biological replicates. Data are represented as means ± SD. A two-way ANOVA was performed to assess statistical significance. *P*-values: 0.9925 (WT vs. WT + USP1i); 0.0203 (PRIMPOL KO vs. PRIMPOL KO + USP1i). (**f**) An experimental scheme depicting how cell viability was measured after challenging cells with cisplatin and USP1 inhibitor (ML323) in eHAP iCas9 cells with indicated genotypes. (**g**) A bar plot depicting cell survival of eHAP iCas9 WT or PRIMPOL KO cells following challenge with 670 nM cisplatin in the absence (darker bars) or presence (light bars) of 4.5 μM USP1 inhibitor following transient knockout of SLFN11. These experiments were performed in N = 3 biological replicates. Data are represented as means ± SD. A two-way ANOVA was performed to assess statistical significance. *P*-values: >0.9999 (WT sgNTC vs. WT sgNTC + USP1i); >0.9999 (WT sgSLFN11 vs. WT sgSLFN11 + USP1i); 0.0032 (PRIMPOL KO sgNTC vs. PRIMPOL KO sgNTC + USP1i); 0.8134 (PRIMPOL KO sgSLFN11 vs. PRIMPOL KO sgSLFN11 + USP1i); 0.8522 (WT sgNTC vs. WT sgSLFN11); <0.0001 (PRIMPOL KO sgNTC vs. PRIMPOL KO sgSLFN11); 0.8160 (WT sgNTC + USP1i vs. PRIMPOL KO sgNTC + USP1i). Source numerical data are available in the source data. Source data

Loss of USP1 or WDR48 rescued cisplatin sensitivity observed in PrimPol KO cells but had no effect in WT cells, as predicted by our screens (Fig. 5a–d). These results were recapitulated using a fixed dose of ML323, a specific inhibitor of the USP1–WDR48 complex^58^ (Extended Data Fig. 6a,b). These results were also observed in a second SLFN11-expressing cell line, A673 (Extended Data Fig. 6d,e). Importantly, WT or PrimPol KO cells treated with a fixed dose of USP1 inhibitor did not impact cell growth or viability in unchallenged conditions (Extended Data Fig. 6c). Together, these data suggest that loss of USP1–WDR48 complex activity confers resistance to cisplatin specifically in cells deficient in repriming.

As loss of USP1–WDR48 complex activity rescued cisplatin sensitivity in PrimPol KO cells and this phenotype is entirely dependent on SLFN11-mediated and GCN2-mediated apoptosis, we hypothesized that USP1 promotes SLFN11 activation and induction of apoptosis by inducing RPA exhaustion. To test this hypothesis, we performed QIBC experiments to determine the levels of RPA exhaustion in eHAP iCas9 WT or PrimPol KO cells that have had USP1 or SLFN11 transiently knocked out (Fig. 5e,f). To avoid any effects of SLFN11-dependent apoptosis on masking RPA exhaustion levels, we performed all experiments in SLFN11-deficient cells. The level of cisplatin-induced RPA exhaustion in PrimPol KO cells was threefold to fourfold higher than in WT cells (Fig. 5g,h). Strikingly, loss of USP1 in PrimPol KO cells reduced the level of cisplatin-induced RPA exhaustion by twofold to threefold when compared with USP1-proficient PrimPol KO cells (Fig. 5g,h). Together, our data indicate that loss of USP1 significantly reduces the levels of cisplatin-induced RPA exhaustion in cells lacking repriming.

**Extended Data Fig. 7:**
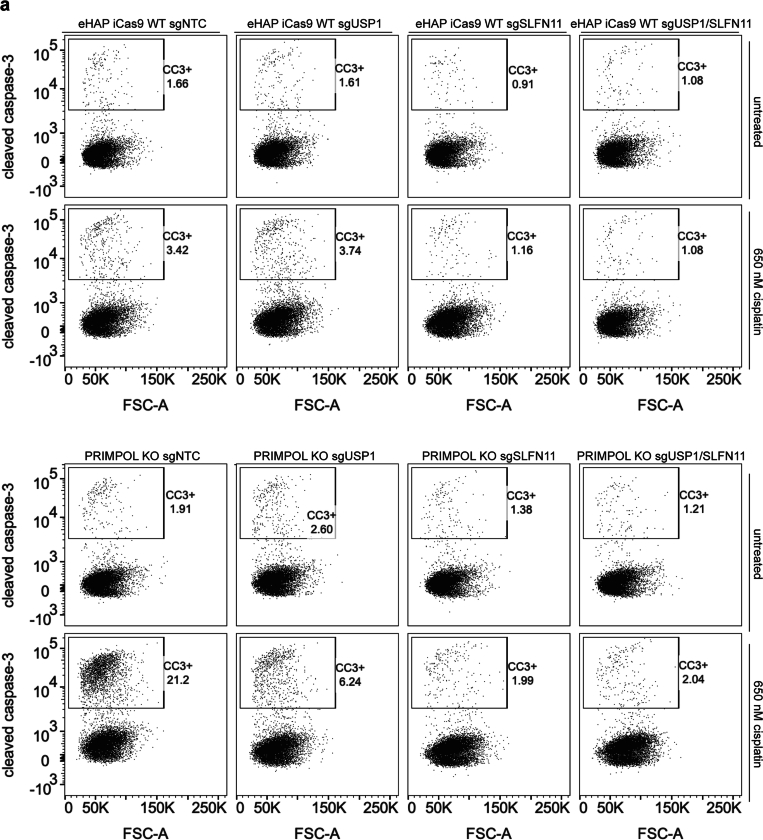
Loss of USP1 restricts SLFN11-dependent apoptosis is response to cisplatin when PRIMPOL is inactivated. (**a**) A series of representative dot plots depicting how apoptosis was measured in cells with indicated genotypes by quantifying the percentage of cells that stained positively for cleaved caspase-3. These dot plots are related to the quantification of apoptosis shown in Fig. 5j.

We hypothesized that the rescue of RPA exhaustion observed on loss of USP1 would lead to a reduction in SLFN11-dependent apoptosis and cell death. Loss of USP1 significantly rescued SLFN11-dependent apoptosis in PrimPol KO cells challenged with cisplatin and provided no benefit in SLFN11-deficient cells (Fig. 5i,j and Extended Data Fig. 7). As observed with RPA exhaustion and apoptosis, USP1 inhibition significantly rescued cell viability of PrimPol KO cells challenged with cisplatin in an SLFN11-dependent manner (Extended Data Fig. 6f,g). Together, these data suggest that loss of USP1 restricts activation of SLFN11 and subsequent apoptosis.

USP1 targets two major proteins in the DDR for deubiquitination: FANCD2–FANCI and PCNA^59,60^. Deubiquitination of FANCD2–FANCI and PCNA leads to downregulation of the FA and TLS pathways, respectively. As USP1 also drove cisplatin sensitivity in an SLFN11-dependent manner in PrimPol KO cells, we sought to determine whether downregulation of the FA or TLS pathways was responsible for driving RPA exhaustion and SLFN11-dependent cell death.

**Extended Data Fig. 8:**
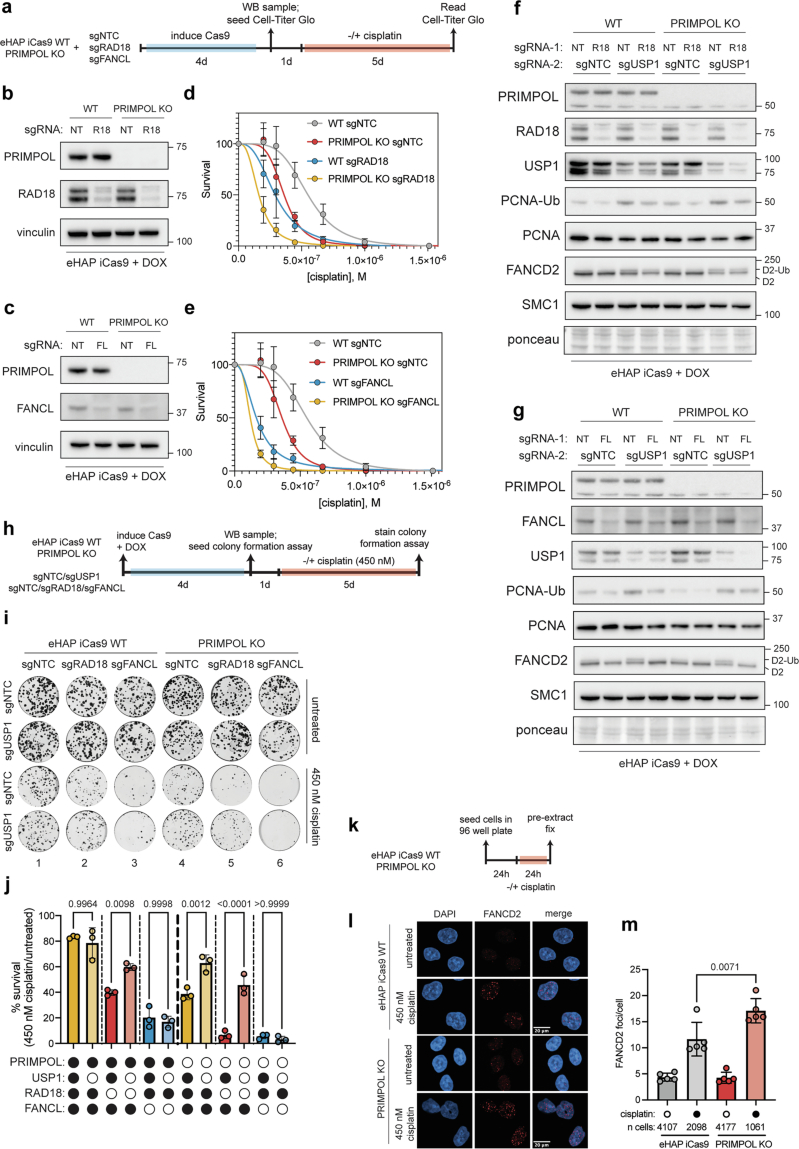
Upregulation of the Fanconi Anaemia pathway compensates for loss of PRIMPOL/RAD18 in response to cisplatin lesions. (**a**) A schematic showing how RAD18 or FANCL was knocked out in eHAP iCas9 WT or PRIMPOL KO cells and subsequently challenged with cisplatin. (**b**) A Western blot showing PRIMPOL and RAD18 protein levels in eHAP iCas9 WT or PRIMPOL KO cells upon loss of RAD18 (R18). (**c**) A cisplatin dose response curve for eHAP iCas9 WT or PRIMPOL cells upon loss of RAD18. These experiments were performed in N = 3 biological replicates. Data are represented as means ± SD. These experiments were performed using the same WT sgNTC and PRIMPOL KO sgNTC samples depicted in (m). (**d**) A Western blot showing PRIMPOL and FANCL protein levels in eHAP iCas9 WT or PRIMPOL KO cells upon loss of FANCL (FL). (**e**) A cisplatin dose response curve for eHAP iCas9 WT or PRIMPOL KO cells upon loss of FANCL. eHAP iCas9 WT and PRIMPOL KO curves are the same as those shown in (d), as these experiments were performed at the same time. These experiments were performed in N = 3 biological replicates. Data are represented as means ± SD. (**f**) A series of Western blots showing indicated protein levels in eHAP iCas9 WT or PRIMPOL cells upon loss of USP1 and/or RAD18 (R18). (**g**) A series of Western blots showing indicated protein levels in eHAP iCas9 WT or PRIMPOL KO cells upon loss of USP1 and/or FANCL (FL). (**h**) An experimental scheme for how USP1/RAD18/FANCL proteins were knocked out in eHAP iCas9 WT or PRIMPOL KO cells and subsequently challenged with cisplatin in a colony formation assay. (**i**) Representative images of colony formation assays in indicated cell backgrounds in untreated conditions or upon treatment with 450 nM cisplatin. (**j**) Quantification of colony survival in the colony formation assays represented in (d) normalised to untreated samples within each genotype. These experiments were performed in N = 3 biological replicates. Data are represented as means ± SD. A two-way ANOVA was performed to test statistical significance. *P*-values: 0.9964 (WT sgNTC vs. WT sgUSP1); 0.0098 (WT sgRAD18/NTC vs. WT sgRAD18/sgUSP1); 0.9998 (WT sgFANCL/sgNTC vs. WT sgFANCL/USP1); 0.0012 (PRIMPOL KO sgNTC vs. PRIMPOL KO sgUSP1); <0.0001 (PRIMPOL KO sgRAD18/NTC vs. PRIMPOL KO sgRAD18/USP1); >0.9999 (PRIMPOL KO sgFANCL/NTC vs. PRIMPOL KO sgFANCL/USP1). (**k**) An experimental schematic describing how chromatin-bound immunofluorescence was performed. (**l**) Representative micrographs depicting DNA staining (DAPI), FANCD2 staining, and a merged image of DAPI/FANCD2 staining. (**m**) A bar plot showing chromatin-bound FANCD2 foci numbers in eHAP iCas9 WT or PRIMPOL KO cells treated with 450 nM cisplatin. These experiments were performed in N = 5 biological replicates. Data are represented as means ± SD. A two-way ANOVA was performed to assess statistical significance. P-values: 0.0071 (WT + cisplatin vs. PRIMPOL KO + cisplatin). Source numerical data and unprocessed blots are available in the source data. Source data

First, we wanted to determine whether loss of the FA or TLS pathway was synthetic lethal with loss of repriming in response to cisplatin as predicted in our targeted CRISPR–Cas9 screens (Fig. 2b). Indeed, loss of either RAD18 or FANCL led to an additive cisplatin sensitivity in cells lacking repriming (Extended Data Fig. 8a–e). Second, we wanted to determine whether upregulation of the FA or TLS pathway was responsible for rescuing cisplatin sensitivity in cells lacking repriming after USP1 depletion (Extended Data Fig. 8f–h). Loss of RAD18 did not reverse the cisplatin resistance observed on knockout of USP1 in PrimPol KO cells, whereas loss of FANCL completely reversed this phenotype (Extended Data Fig. 8i,j). Strikingly, loss of USP1 partially rescued the exquisite cisplatin sensitivity observed in double RAD18–PrimPol KO cells, underlining the importance of the FA pathway in repairing cisplatin lesions, particularly when TLS and repriming are inactivated (Extended Data Fig. 8i,j). Indeed, we observed an increase in the number of FANCD2 foci present in PrimPol KO cells challenged with cisplatin, suggesting a compensatory upregulation of the FA pathway when repriming is inactivated (Extended Data Fig. 8k–m). Together, our data implicate USP1-driven downregulation of the FA pathway as the mechanism behind the increased cisplatin-induced RPA exhaustion and SLFN11-dependent apoptosis in cells lacking repriming.

### Inhibition of DNA polymerase α potently induces RPA exhaustion and SLFN11-dependent cell death

**Fig. 6:**
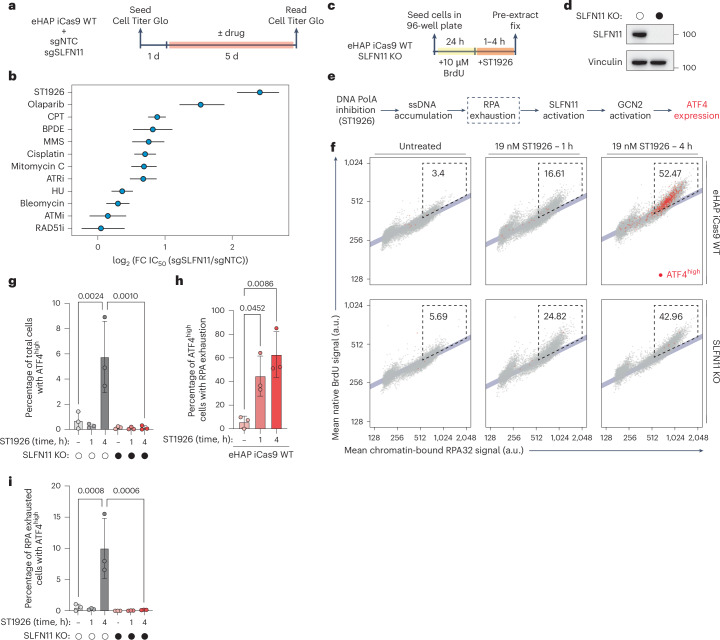
Inhibition of DNA polymerase α potently induces RPA exhaustion and SLFN11-dependent cell death. **a**, An experimental scheme depicting how cell survival was measured in cells with indicated genotypes after challenge with 12 different drugs targeting the DDR. **b**, A Forest plot depicting the log_2_(fold-change) of IC_50_ values derived from dose–response curves of the indicated drugs when SLFN11 was transiently knocked out versus a non-targeting control. Coloured dots represent calculated mean IC_50_ values, whereas black lines indicate 95% confidence intervals of each calculated mean IC_50_ value. **c**, An experimental scheme depicting how RPA exhaustion was assessed using QIBC after treatment with DNA polymerase α inhibitor (ST1926). **d**, A western blot depicting SLFN11 protein levels in eHAP iCas9 WT and an isogenic SLFN11 KO clone. **e**, A scheme of how SLFN11 activation was measured by staining cells for chromatin-bound ATF4 after ST1926-induced RPA exhaustion. **f**, Representative scatter plots measuring mean ssDNA or BrdU (*y* axis) versus mean chromatin-bound RPA32 signals (*x* axis). Linearity between the ssDNA and chromatin-bound RPA32 signals is depicted as a light-blue line. Cells undergoing RPA exhaustion are highlighted within the dashed box, with representative percentages of cells undergoing RPA exhaustion indicated. Cells with high levels of chromatin-bound ATF4 (ATF4^high^) are coloured red, whereas ATF4^−^ cells are shown in grey. **g**, A bar plot depicting the percentage of cells with indicated genotype and treatment with ATF4^high^ (high ATF4 activation). These experiments were performed in *n* = 3 biological replicates. Data are presented as mean ± s.d. A one-way ANOVA was performed to assess biological significance. *P* values: 0.0024 (WT untreated versus WT 4 h ST1926); and 0.0010 (WT 4-h ST1926 versus *SLFN11* KO 4-h ST1926). **h**, A bar plot depicting the percentage of eHAP iCas9 WT cells with high ATF4 activation that are undergoing RPA exhaustion. These experiments were performed in *n* = 3 biological replicates. A one-way ANOVA was performed to assess biological significance. *P* values: 0.0452 (untreated versus 1-h ST1926); and 0.0086 (untreated versus 4-h ST1926). **i**, A bar plot depicting the percentage of cells undergoing RPA exhaustion that exhibit ATF4 activation (ATF4^high^). These experiments were performed in *n* = 3 biological replicates. A one-way ANOVA was performed to assess biological significance. *P* values: 0.0008 (WT untreated versus WT 4-h ST1926); and 0.0006 (WT 4-h ST1926 versus SLFN11 KO 4-h ST1926). Source numerical data and unprocessed blots are available in the source data. Pol A, polymerase A. Source data

**Extended Data Fig. 9:**
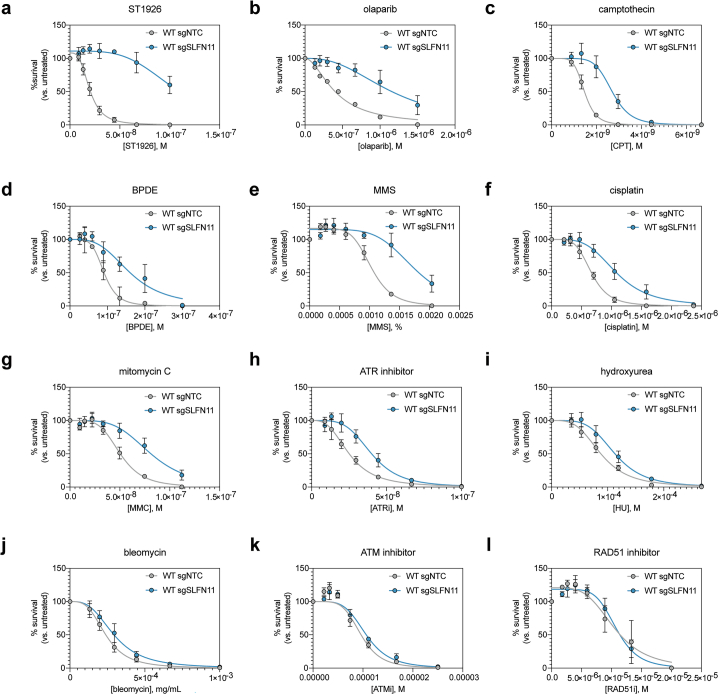
Drugs differentially activate the SLFN11 cell death response. (**a**) A dose response curve measuring cell viability in the presence of indicated concentrations of ST1926, a DNA polymerase alpha inhibitor. (**b**) A dose response curve measuring cell viability in the presence of indicated concentrations of olaparib, a PARP inhibitor. (**c**) A dose response curve measuring cell viability in the presence of indicated concentrations of camptothecin (CPT), a topoisomerase I inhibitor. (**d**) A dose response curve measuring cell viability in the presence of indicated concentrations of benzo(a)pyrene-7,8-dihydrodiol-9,10-epoxide (BPDE). (**e**) A dose response curve measuring cell viability in the presence of indicated concentrations of methyl methanesulfonate (MMS). (**f**) A dose response curve measuring cell viability in the presence of indicated concentrations cisplatin. (**g**) A dose response curve measuring cell viability in the presence of indicated concentrations of mitomycin C (MMC). (**h**) A dose response curve measuring cell viability in the presence of indicated concentrations of AZD6738, an ATR inhibitor. (**i**) A dose response curve measuring cell viability in the presence of indicated concentrations of hydroxyurea (HU), an inhibitor of ribonucleotide reductase. (**j**) A dose response curve measuring cell viability in the presence of indicated concentrations of bleomycin. (**k**) A dose response curve measuring cell viability in the presence of indicated concentrations of KU-5593, an ATM inhibitor. All experiments were performed in N = 3 biological replicates. Data are represented as means ± SD. (**l**) A dose response curve measuring cell viability in the presence of indicated concentrations of B02, a RAD51 inhibitor. Source numerical data are available in the source data. Source data

Having established that cells lacking repriming undergo RPA exhaustion and SLFN11-dependent cell death when challenged with cisplatin, we wanted to determine whether this mechanism is broadly applicable to other types of DNA damage, independent of PrimPol status. To address this, we transiently knocked out SLFN11 in WT eHAP iCas9 cells and challenged them with 12 different genotoxic agents or inhibitors of DDR pathways (Fig. 6a and Extended Data Fig. 9a–l). Strikingly, cells treated with ST1926, a selective chemical inhibitor of DNA polymerase α^61^, underwent a potent SLFN11-dependent death response (Fig. 6b and Extended Data Fig. 9a). Other drugs, such as the poly(ADP ribose polymerase) inhibitor, olaparib, or the topoisomerase I inhibitor, camptothecin, also induced a strong SLFN11-dependent death response (Fig. 6b and Extended Data Fig. 9b,c). Conversely, drugs targeting DSB repair pathways (RAD51 or ataxia-telangiectasia mutated (ATM) inhibitor) or inducing DSBs (bleomycin) did not elicit a strong SLFN11-dependent cell death (Fig. 6b). Surprisingly, hydroxyurea, a potent inducer of ssDNA at replication forks, failed to robustly activate SLFN11-dependent cell death (Fig. 6b). We hypothesized that this could be due to ST1926 and hydroxyurea inducing RPA exhaustion at different levels, as previously described^9,10^.

**Extended Data Fig. 10:**
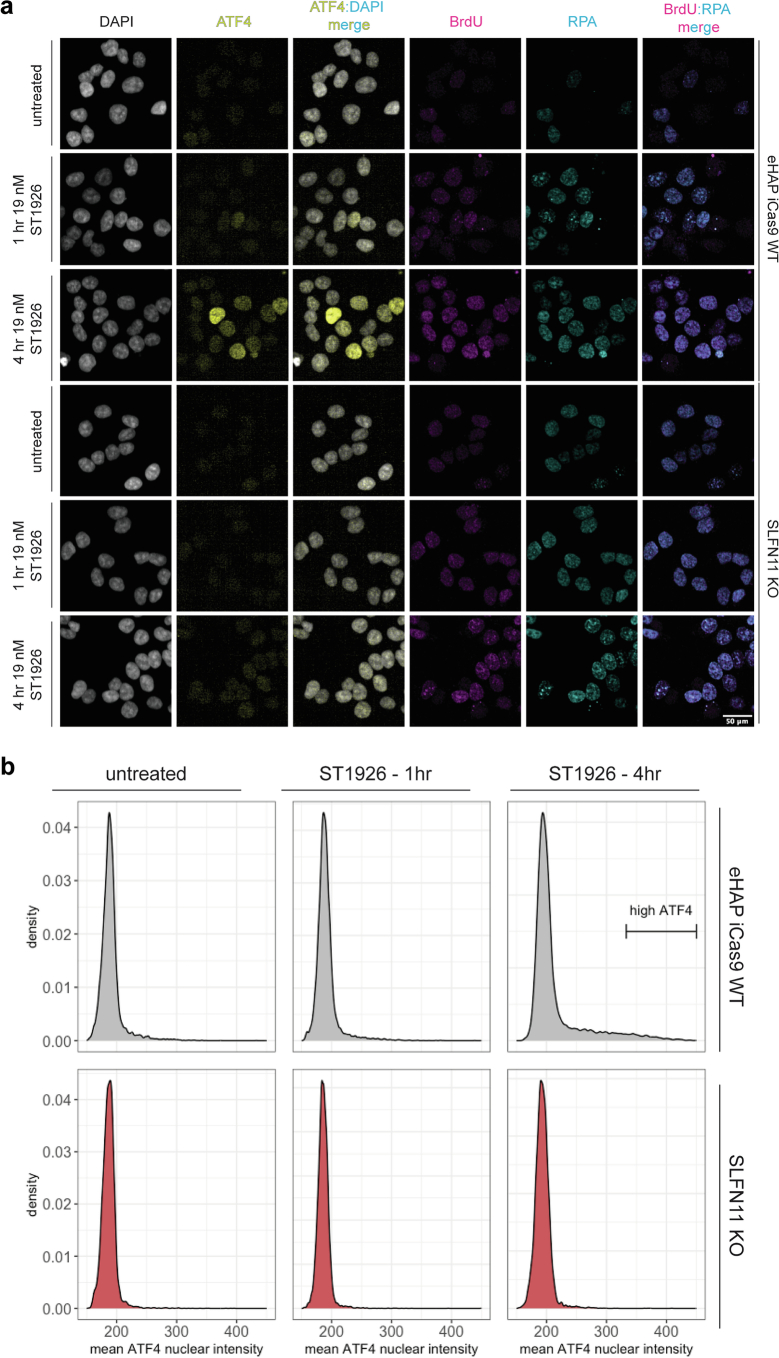
DNA polymerase alpha inhibition induces RPA exhaustion and ATF4 activation in a SLFN11-dependent manner. (**a**) Representative micrographs related to QIBC experiments shown in Fig. 4f. (**b**) Representative histograms depicting mean chromatin-bound ATF4 signal in indicated conditions.

As ST1926 potently induces RPA exhaustion and SLFN11-dependent cell death, we wanted to determine whether ST1926-induced RPA exhaustion could activate SLFN11. We utilized QIBC to simultaneously determine the levels of RPA exhaustion and SLFN11 activation in eHAP iCas9 WT cells or an isogenic SLFN11 KO clone (Fig. 6c,d). We were unable to detect SLFN11 chromatin binding under the rigorous pre-extraction conditions required for native BrdU or chromatin-bound RPA QIBC experiments. Thus, we stained for chromatin-bound ATF4, the master transcription factor activated by the GCN2-dependent ISR^62^, as a proxy for SLFN11 activation (Fig. 6e). Indeed, eHAP cells treated with ST1926 potently activated ATF4 in an SLFN11-dependent manner, providing confidence in our approach (Extended Data Fig. 10a,b). We utilized a conservative threshold for ATF4 activation by only scoring the top 25% of ATF4^+^ cells as ATF4^high^ (Extended Data Fig. 10b). Given that only high or chronic levels of ISR activation are proapoptotic^62,63^, we wanted to ensure that both replicate reproducibility and accurate scoring of only the cells with the highest levels of ATF4. Low doses of ST1926 rapidly induced RPA exhaustion after 1 h of treatment in both WT and SLFN11 KO cells (Fig. 6f). After 4 h of ST1926 treatment, RPA exhaustion increased in both WT and SLFN11 KO cells; however, ATF4^high^ cells were observed only in WT cells (Fig. 6e,f). Consistent with our model that RPA exhaustion activates SLFN11, >60% of WT cells with ATF4^high^ were undergoing RPA exhaustion while 10% of WT cells undergoing RPA exhaustion exhibited high levels of ATF4 (Fig. 6f,h,i). Together, these data suggest that inhibition of DNA polymerase α results in robust RPA exhaustion that can activate the ISR in an SLFN11-dependent manner.

**Fig. 7:**
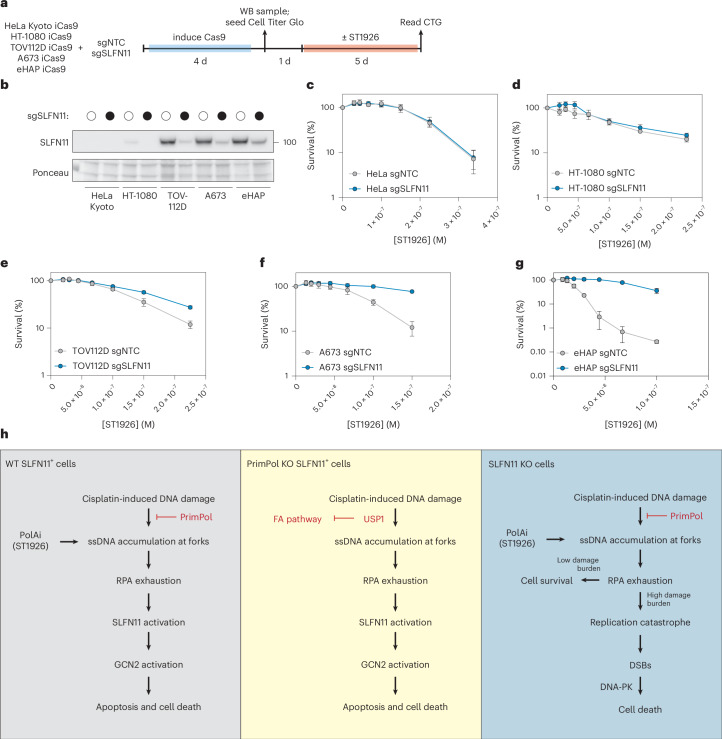
Inhibition of DNA polymerase α induces SLFN11-dependent cell death in multiple cell lines. **a**, An experimental scheme depicting how cell viability was measured in indicated cell lines after transient knockout of SLFN11 and challenge with DNA polymerase α inhibitor (ST1926). **b**, A western blot depicting SLFN11 protein levels after transient knockout of SLFN11 in the indicated cell lines. **c**–**g**, Dose–response curves of HeLa Kyoto iCas9 (**c**), HT-1080 iCas9 (**d**), TOV112D iCas9 (**e**), A673 iCas9 (**f**) and eHAP iCas9 (**g**) cells challenged with the indicated doses of DNA polymerase α inhibitor (ST1926). Experiments were performed in *n* = 3 biological replicates. Data are presented as mean ± s.d. **h**, In WT cells expressing SLFN11 (blue panel), PrimPol repriming activity restricts accumulation of ssDNA at cisplatin-stalled replication forks caused by uncoupling of the helicase and DNA polymerase, thereby preventing SLFN11 activation and cell death. Similarly, inhibition of DNA polymerase α uncouples DNA replication on each strand to drive ssDNA accumulation at forks in a PrimPol-independent manner. In PrimPol-deficient cells (green panel), cisplatin-induced DNA damage drives accumulation of ssDNA at replication forks in a USP1-dependent manner, leading to RPA exhaustion and SLFN11 activation. USP1-dependent downregulation of the FA pathway allows for full SLFN11 activation and subsequent ribosome stalling, ISR activation and cell death, as previously described^38^. In SLFN11-deficient cells (red panel), cisplatin-induced DNA damage or inhibition of DNA polymerase α causes ssDNA accumulation at forks and RPA exhaustion. Loss of SLFN11 prevents signal transduction through ribosome stalling and GCN2, preventing SLFN11-dependent cell death. Instead, prolonged RPA exhaustion leads to fork breakage and replication catastrophe as previously described^9^, leading to DSBs that are repaired by the NHEJ pathway, leading to cell death. Source numerical data and unprocessed blots are available in the source data. PolAi, Pol A inhibitor. Source data

Having established ST1926 as a potent inducer of both RPA exhaustion and SLFN11-dependent cell death, we investigated whether this was broadly applicable to other SLFN11-expressing cell lines. We generated five cancer cell lines harbouring an inducible Cas9 that express different levels of SLFN11: HeLa Kyoto, HT-1080, TOV112D, A673 and eHAP. These cells were challenged with ST1926 treatment after transient knockout of SLFN11 (Fig. 7a,b). SLFN11-dependent cell death was observed only in cell lines that express high amounts of SLFN11 (Fig. 7c–g). Together, these data suggest that inhibition of DNA polymerase α induces SLFN11-dependent cell death through a mechanism that is conserved across different cancer cell types.

## Discussion

Our findings demonstrate that RPA exhaustion and exposure of ssDNA are the trigger for SLFN11 activation in response to replication-stalling lesions. Our study also reconciles a long-standing discrepancy between mechanistic observations that PrimPol reprimes downstream of DNA lesions while also being dispensable for cell viability in response to those same DNA lesions, which we attribute to the expression status of SLFN11 in cells.

Our serendipitous finding that chronic myelogenous leukaemia eHAP cancer cells lacking PrimPol-mediated repriming are sensitive to cisplatin (Fig. 1a–i) was initially surprising, because previous studies showed that other cell lines such as RPE-1, UWB1.289, HEK293T and U2OS do not exhibit this phenotype^14,20,22,33^. Our targeted CRISPR–Cas9 screens identified SLFN11 as a driver of cisplatin sensitivity, particularly in cells deficient in PrimPol-mediated repriming (Fig. 2a). Using a panel of 12 cancer and non-cancerous cell lines, we showed that SLFN11 expression level is broadly correlated with cisplatin sensitivity when PrimPol-mediated repriming is inactivated (Fig. 2d–n and Extended Data Fig. 2a–e). The lack of cisplatin sensitivity observed previously in other cell lines lacking repriming can be explained by the silencing of SLFN11 in these cell lines. Indeed, targeted knockout of SLFN11 or inhibition of the downstream ISR kinase GCN2 rescued both apoptosis and cisplatin sensitivity observed in eHAP PrimPol KO cells to levels indistinguishable from WT cells (Fig. 3a–h and Extended Data Fig. 4a–d). Importantly, our study implicates the GCN2-mediated ISR as being primarily responsible for SLFN11-dependent apoptosis, in contrast with previous work, which implicated the JNK-mediated ribotoxic stress response or impaired ribosome biogenesis^38,39^. This is probably due to a difference in dose and treatment time of genotoxic agents used in our work versus those used in other studies. Future work is needed to understand how different burdens of replication stress induce differential SLFN11-dependent responses.

Intriguingly, we found that the tRNA nuclease, ssDNA-binding and ATPase or helicase activities of SLFN11 were all required to confer cisplatin sensitivity in PrimPol KO cells (Fig. 3i–k), in agreement with previous work^39^. Although the requirements for the ssDNA-binding and tRNA nuclease activities are clear^38,64^, the purpose of the helicase activity of SLFN11 in promoting apoptosis has remained ambiguous^37,39,45,47^. We favour a model whereby the ATPase or helicase activity of SLFN11 is required to release SLFN11 from DNA to effectively cleave tRNAs. However, future studies focusing on further dissecting the distinct functions of SLFN11 and how each promotes p53-independent apoptosis are warranted.

SLFN11 preferentially binds ssDNA in vitro^64^ and this activity is required for translation shutdown, p53-independent apoptosis^38,39^, ssDNA-induced cell death^65^ and cisplatin sensitivity in PrimPol KO cells (Fig. 3i–k). However, the endogenous source of ssDNA that activates SLFN11 was previously unknown. To avoid spontaneous induction of apoptosis, the activation of SLFN11 must be tightly regulated. It is unlikely that every stalled replication fork would activate SLFN11-dependent cell death, because many drugs, such as hydroxyurea, induce fork stalling but do not robustly activate the SLFN11–GCN2 cell death pathway (Fig. 6b). We hypothesized that a potential source of ssDNA under heightened replication stress was RPA exhaustion, a rare yet catastrophic event that serves as a potent source of ssDNA in the cell^9^. Indeed, we observed more cells undergoing RPA exhaustion under conditions that induce SLFN11-dependent cell death (Figs. 4c,d, 6 and 7). Importantly, RPA exhaustion induced by siRNA targeting of RPA2 led to a SLFN11–GCN2-dependent sensitization to cisplatin when repriming was inactivated (Fig. 4h,i). RPA exhaustion triggering SLFN11-dependent apoptosis also explains why drugs like hydroxyurea do not potently activate an SLFN11-dependent response, whereas drugs like ST1926 do (Fig. 6b). Hydroxyurea alone does not induce RPA exhaustion even though it induces ssDNA accumulation at forks, probably through its potent induction of ATR and subsequent prevention of origin firing^10^. However, inhibition of DNA polymerase α induces rapid ssDNA accumulation, potent induction of RPA exhaustion^10^ and SLFN11 activation (Fig. 6e).

Another hallmark of RPA exhaustion is the generation of double-stranded DNA breaks after a replication fork catastrophe and fork breakage. Previous work connecting RPA exhaustion and replication fork catastrophe has been performed in U2OS, a SLFN11-negative cell line^9^. Strikingly, we observed RPA exhaustion-induced DSBs predominantly in SLFN11-negative cells that were preferentially acted on by DNA-PK, leading to cell death (Figs. 4e–f,k,l). Our interpretation of these data is that RPA exhaustion triggers SLFN11-dependent apoptosis before replication fork catastrophe (Fig. 7h). When SLFN11 is not expressed, cells undergoing RPA exhaustion do not undergo apoptosis but instead undergo canonical, replication fork catastrophe, DSB formation, potentially engaging the NHEJ pathway (Fig. 7h).

Our work also identified the USP1–WDR48 DUB complex as a modulator of SLFN11 activation at the replication fork. In cells lacking repriming, USP1 drives cisplatin-induced RPA exhaustion, resulting in induction of SLFN11-dependent apoptosis and cell death (Fig. 5). We identified upregulation of the FA pathway as responsible for preventing SLFN11-dependent apoptosis when USP1 is knocked out (Extended Data Fig. 7). Our interpretation of these data is that cells lacking PrimPol become addicted to the FA pathway to resolve cisplatin lesions. However, we cannot rule out that FA-mediated repair of cisplatin lesions is delayed in the absence of PrimPol^25^, potentially leading to a temporary increase in FANCD2 foci. However, as the FA pathway and PrimPol operate additively in response to cisplatin (Extended Data Fig. 7e) and upregulation of the FA pathway rescues cisplatin sensitivity in PrimPol KO cells (Extended Data Fig. 7j), we favour the model that PrimPol and the FA pathway act separately in response to cisplatin.

In conclusion, our findings describe a fundamental insight into the mechanism of SLFN11 activation at replication-stalling lesions, revealing a critical role in sensing and eradicating cells experiencing RPA exhaustion. We propose that the ATR-dependent, intra-S-phase checkpoint senses and signals replication stress by virtue of the targeting of ATR to RPA-coated ssDNA by ATR-interacting protein (ATRIP), which is required to stabilize the replisome, invoke repair and regulate origin firing. If this response is overwhelmed or RPA becomes limiting due to RPA exhaustion, this would create the dangerous scenario of unprotected ssDNA prone to nucleolytic attack and replication catastrophe. Our work reveals that SLFN11 has evolved to sense RPA exhaustion and, on activation, triggers a ‘pathway of last resort’ to eliminate cells with heightened replication stress. Given that elevated replication stress is a hallmark of cancers, RPA exhaustion would create a strong selective pressure to epigenetically silence SLFN11 to allow cancer cell survival, which would explain its frequent inactivation in >50% of treatment-naive tumours.

## Methods

### Cell lines

The eHAP-inducible Cas9 (iCas9) cells were generated previously^66^ and maintained in Isocove’s modified Dulbecco’s medium (Gibco, catalogue number 12440053) supplemented with 10% tetracycline-free fetal bovine serum (FBS; Pan Biotech, catalogue number P30-3602) and penicillin–streptomycin (Gibco, catalogue number 15140122). HeLa Kyoto, U2OS, DU145, A549, HEK293A, HEK293 FT, HCT116, TOV112D and A673 cells were maintained in Dulbecco’s modified Eagle’s medium (Gibco, catalogue number 41966029) supplemented with 10% tetracycline-free FBS and penicillin–streptomycin. RPE-1 p53 KO cells were a kind gift of K. Vousden and maintained in Dulbecco’s modified Eagle’s medium/F12 medium (Gibco, catalogue number 11320033) supplemented with 10% tetracycline-free FBS and penicillin–streptomycin. HT-1080 iCas9 cells were generated as described in ‘Inducible Cas9 parental cell line generation’ and maintained in Eagle’s minimum essential medium (LGC, catalogue number 30-2003) supplemented with 10% FBS and penicillin–streptomycin. NCI-H460 iCas9 cells were generated as described in ‘Inducible Cas9 parental cell line generation’ and maintained in Roswell Park Memorial Institute 1640 medium (Gibco, catalogue nnumber 11530586) supplemented with 10% FBS and penicillin–streptomycin. All cells utilized in this study were grown at 37 °C and 5% CO_2_ in humidified cell culture incubators. All cell lines in this study were obtained from the Francis Crick Institute Cell Science Scientific Technology Platform.

### Chemicals and DNA-damaging agents

Cisplatin (Sigma-Aldrich, catalogue number P4394) was dissolved in phosphate-buffered saline (PBS) to a stock concentration of 5 mM and stored at room temperature, protected from light, for 2–3 weeks before making a fresh stock. Hydroxyurea (Sigma-Aldrich, catalogue number H627) was dissolved fresh in sterile water to a stock concentration of 100 mM before each experiment. Other chemicals used in this study were purchased as solid powder and reconstituted in dimethyl sulfoxide, aliquoted and stored at −20 °C or −80 °C according to the manufacturer’s instructions before use: doxycycline (Sigma-Aldrich, catalogue number M0503), blasticidin (Thermo Fisher Scientific, catalogue number A1113903), puromycin (Thermo Fisher Scientific, catalogue number A1113803), BPDE (Santa Cruz, catalogue number sc-503767), MMC (Sigma-Aldrich, catalogue number M4287), olaparib (Selleckchem, catalogue number S1060), aphidicolin (Sigma-Aldrich, catalogue number A0781), ST1926 (Sigma-Aldric, catalogue number SML2061), bleomycin (Sigma-Aldrich, catalogue number B8416), methyl methane sulfonate (Thermo Fisher Scientific, catalogue number H55120.06), RAD51 inhibitor B02 (Selleckchem, catalogue number S8434), ATR inhibitor AZD6738 (Selleckchem, catalogue number S7693), camptothecin (Selleckchem, catalogue number S1288), ATM inhibitor KU-55933 (Selleckchem, catalogue number S1092), GCN2i A-92 (Axon Medchem, catalogue number 2720), USP1 inhibitor ML323 (Selleckchem, catalogue number S7529) and DNA-PKi NU7441 (Selleckchem, catalogue number S2638).

### SiRNA transfection and knockdown

ON-TARGETplus SMARTPool non-targeting siRNA (catalogue number D-001810-10-05) or siRNA targeting human RPA2 (catalogue number L-017058-01-0005) was purchased from Horizon Discovery and resuspended in siRNA dilution buffer (Dharmacon, catalogue number B-002000-UB-100) to a concentration of 20 μM. The eHAP iCas9 WT or PrimPol KO cells were transfected with the indicated concentrations of siRNA targeting the *RPA2* gene using lipofectamine RNAiMAX (Thermo Fisher Scientific, catalogue number 13778150) while keeping total siRNA concentration constant by buffering with non-targeting siRNA.

### Plasmids and cDNA

PrimPol cDNA was purchased from OriGene (catalogue number SC100629). PrimPol cDNA was amplified using Gateway primers PrimPol-gateway-F and PrimPol-gateway-R (Supplementary Table 2) and cloned into the pDONR221 plasmid (Thermo Fisher Scientific, catalogue number 12536017). PrimPol was subcloned into the pLEX_307 (pLenti EF1a) destination vector (Addgene, catalogue number 41392). PrimPol AxA (Asp114Ala, Glu116Ala) and CH (Cys419Gly, His426Tyr) mutants were introduced into this plasmid using the Q5 Site-Directed Mutagenesis kit (NEB), with primers shown in Supplementary Table 2.

SLFN11 cDNA was purchased from Dharmacon. SLFN11 cDNA was amplified and cloned into a pDONR221 plasmid as described above. SLFN11 Glu209Ala, Lys652Asp and Glu669Gln mutants were generated using the Q5 Site-Directed Mutagenesis kit with the primers shown in Supplementary Table 2. Finally, WT or mutant SLFN11 cDNAs were cloned into a Piggyback EF1a-driven Gateway plasmid. These plasmids were transfected into the indicated cells alongside the PiggyBac Transposase plasmid to generate stable cell lines expressing SLFN11.

### MisFIT cloning to tune PrimPol expression

PrimPol expression was downregulated by cloning misFIT sequences^53^ complementary to the mIR-17 microRNA with indicated mutations and spacer sequences into the 3ʹ-UTR downstream of WT PrimPol, PrimPol AxA or PrimPol CH mutants previously cloned into pLEX_307. A complete list of oligonucleotides used for misFIT cloning is shown in Supplementary Table 2.

### Cloning of sgRNAs into pLenti-sgRNA-puro or pLenti-sgRNA-hygro and pLentiCRISPRv2 plasmids

The sgRNAs targeting the indicated genes were cloned into pLenti-sgRNA-puro, pLenti-sgRNA-hygro or pLentiCRISPRv2 using available protocols from the Zhang Lab (Addgene, catalogue nos. 52963, 139462 and 52961). The sgRNA sequences were sourced from the Brunello sgRNA library^67^ or designed in house (Supplementary Table 1).

### Lentivirus preparation and infection

Lentiviruses were generated by transfecting HEK293 FT cells with third-generation packaging plasmids VSVG, pLP1 (gag or pol) and pLP2 (Rev) and a lentiviral vector of interest. The medium was changed on transfected cells 18 h post-transfection; cells were left for a further 48 h, after which viral supernatants were harvested, filtered with a 0.45-μm filter, aliquoted and frozen at −80 °C.

Cells were prepared for transduction by changing the medium to a low volume of complete medium with added polybrene (Sigma-Aldrich, catalogue number H9268) to a final concentration of 8 μg ml^−1^. Cells were then transduced by adding viral supernatants and either left overnight or immediately spinfected by spinning cell or viral supernatant mixtures at 500*g* and 37 °C for 60 min.

Cell lines were selected using the following concentration of selected agents: eHAP (puromycin: 0.4 μg ml^−1^, hygromycin: 400 μg ml^−1^, blasticidin: 8 μg ml^−1^); HT-1080 (puromycin: 1 μg ml^−1^; blasticidin: 8 μg ml^−1^); NCIH-460 (puromycin: 1.0 μg ml^−1^, blasticidin: 8 μg ml^−1^); A673: (puromycin: 1.0 μg ml^−1^); HeLa Kyoto (puromycin: 1.0 μg ml^−1^); U2OS (puromycin: 1 μg ml^−1^); DU145 (puromycin: 1 μg ml^−1^); A549 (puromycin: 1 μg ml^−1^); HEK293A (puromycin: 1 μg ml^−1^); HCT116 (puromycin: 1 μg ml^−1^); and RPE-1 p53 KO (puromycin: 20 μg ml^−1^).

### Inducible Cas9 parental cell-line generation

HT-1080 iCas9, NCIH-460 iCas9, TOV112D iCas9 and A673 iCas9 parental cell lines were generated by transducing lentiviral particles generated as described in ‘Lentivirus preparation and infection’ harbouring the Edit-R Inducible Lentiviral Cas9 plasmid purchased from Dharmacon or Horizon (Horizon, catalogue number CAS11229). Transduced cells were enriched by selecting with blasticidin (8 μg ml^−1^) for 2–3 d until untransduced cells died. Single-cell clones were isolated from these pools of transduced cells and iCas9 activity was measured as described in ‘Inducible Cas9 activity assay’. Clones exhibiting <5% Cas9 activity in uninduced (no doxycycline) and >90% Cas9 activity in induced (with doxycycline) were used as parental iCas9 cell lines.

### Inducible Cas9 activity assay

Inducible Cas9 clones were assessed for Cas9 activity by transducing lentiviral particles harbouring the pKLV2-U6gRNA5(gGFP)-PGKBFP2AGFP-W or pKLV2-U6gRNA5(gGFP)-PGKmCherry2AGFP-W plasmids (Addgene, catalogue nos. 67980 and 67982). Cells were split into two populations in the absence or presence of 1 μg ml^−1^ of doxycycline to induce Cas9 expression 24 h post-transduction. Cells were then maintained with or without doxycycline for 3 d (changing doxycycline as needed every 2 d). Cells were harvested and BFP–mCherry and GFP populations were assessed using flow cytometry. BFP^+^ cells were gated for GFP^+^ (no Cas9 activity) and GFP^−^ cells (Cas9 activity).

### Generation of CRISPR KO cell lines

To generate eHAP iCas9 PrimPol, FANCD2, SLFN11, RAD18 or USP1 KO clones, an sgRNA targeting PrimPol, FANCD2, SLFN11, RAD18 or USP1 was cloned into pLenti-sgRNA-puro vector. This vector was transfected into eHAP iCas9 WT or PrimPol KO parental cells while Cas9 was induced on addition of 1 μg ml^−1^ of doxycycline. Cells were enriched for those harbouring the vector by selecting with puromycin for 1–2 d while maintaining Cas9 expression with doxycycline. The resulting pool of transfected cells was seeded by limiting dilution and clones were picked and screened by purifying genomic DNA using the PureLink Genomic DNA Mini Kit (Thermo Fisher Scientific, catalogue number K182001) and PCR amplifying and Sanger sequencing for the sgRNA cut site. Positive clones were further screened by western blotting to assess protein levels.

### Generation of cell lines harbouring an integrated Cas9–sgRNA

The indicated parental cell lines were transduced with lentiviral particles harbouring a vector containing either a constitutively expressed sgRNA (pLenti-sgRNA-puro or pLenti-sgRNA-hygro) or an all-in-one Cas9–sgRNA cassette (pLentiCRISPRv2-puro). Cells were selected with appropriate selection agents at the concentrations listed above. Selected pools of cells were then used directly in experiments or single-cell clones were isolated as indicated.

### Whole-cell extracts, chromatin fractionations, SDS–PAGE and western blotting

Whole-cell extracts were isolated by harvesting cells by centrifugation, washing once with PBS, spinning down again and freezing cell pellets at −80 °C. Cells were thawed and resuspended in radioimmunoprecipitation buffer (50 mM Tris-HCl, pH 7.4, 150 mM NaCl, 1% Triton X-100, 0.1% sodium dodecylsulfate (SDS), 0.5% deoxycholate, 1× phosphatase (Phos-Stop, Roche, catalogue number 4906845001) and 1× protease (cOmplete EDTA-free, Roche, catalogue number 11836170001) inhibitor cocktails). Cells were allowed to lyse for 15–20 min on ice, briefly sonicated to shear chromatin, then centrifuged at high speed (30,000*g*) to remove cellular debris. After centrifugation, lysates were quantified for protein concentration using the DC protein assay (BioRad). Cell lysates were normalized to equal protein concentration and mixed with appropriate volumes of NuPAGE LDS sample buffer (4×; Thermo Fisher Scientific, catalogue number NP0008) supplemented with 100 mM 2-mercaptoethanol. Lysates were boiled at 90 °C for 10 min and frozen at −20 °C until ready to run on SDS–polyacrylamide gel electrophoresis (PAGE).

Protein extracts were thawed at room temperature before loading 30–40 μg of total protein on to 4–12% Bis–Tris NuPAGE SDS–PAGE gradient gels or NuPAGE 3–8% Tris-acetate gradient gels (Thermo Fisher Scientific) with an appropriate number of wells. SDS–PAGE gels were run with an applied voltage of 120–180 V for 1–2.5 h.

For chromatin fractionations, whole-cell lysates were split in two and pelleted as above. One half of each sample was resuspended in radioimmunoprecipitation buffer as above. The other half of each sample was extracted with cytoskeleton (CSK) buffer (10 mM PIPES, pH 7.0, 100 mM NaCl, 300 mM sucrose, 1.5 mM MgCl_2_, 5 mM EDTA, 0.5% Triton, 1× phosphatase and 1× protease inhibitor cocktails) on ice for 5 min. Chromatin pellets were harvested by centrifuging at maximum speed for 15 s, after which pellets were washed in CSK buffer and centrifuged again. Chromatin pellets were resuspended in 1× NuPAGE LDS sample buffer, sonicated and boiled as with whole-cell lysates.

Proteins were transferred to nitrocellulose western blotting membranes (GE Healthcare or Amersham) by applying a constant amperage of 400 mA for 1 h. Membranes were stained with Ponceau S stain (Thermo Fisher Scientific) and imaged to assess protein loading. Membranes were then blocked using 5% non-fat milk powder dissolved in Tris-buffered saline with Tween 20 (TBS-T) for 1 h at room temperature. Primary antibodies diluted in 5% non-fat milk powder in TBS-T were applied overnight at 4 °C. Non-specific proteins were washed away with TBS-T 3× for at least 5 min per wash. Secondary horseradish peroxidase-conjugated antibodies were diluted 1:2,000 in 5% non-fat milk powder dissolved in TBS-T and applied to membranes for 1 h at room temperature. Membranes were then thoroughly washed at least 5× with TBS-T before the addition of chemiluminescence reagent (BioRad, Clarity or ClarityMAX). Membranes were then imaged using a BioRad ChemDoc imaging system.

Primary antibodies were utilized at the following concentrations: PrimPol^17^ (Proteintech, catalogue number 29824-1-AP, 1:1,000); PCNA (Santa Cruz, catalogue number sc-56, 1:1,000); vinculin (Sigma-Aldrich, catalogue number V9131, 1:5,000); RPA32 pS33 (Bethyl, catalogue number A300-246A, 1:1,000); RPA32 (abcam, catalogue number ab2175, 1:500); Chk1 pSer345 (Cell Signaling Technology, catalogue number 9664S, 1:1,000); Chk1 (Sigma-Aldrich, catalogue number C9358, 1:500); pGCN2 T899 (abcam, catalogue number 75836, 1:1,000); *SLFN11* (Santa Cruz, catalogue number sc-374339, 1:500); glyceraldehyde 3-phosphate dehydrogenase (Abcam (6C5), catalogue number ab8245, 1:5,000); USP1 (Bethyl, catalogue number A301-699A, 1:1,000 or Proteintech, catalogue number 14346-1-AP, 1:1,000); WDR48 (Proteintech, catalogue number 16503-1-AP, 1:600); PCNA Ub K164 (Cell Signaling Technology, catalogue number 13439, 1:1,000); SMC1 (abcam, catalogue number ab21583, 1:1,000); RAD18 (Bethyl, catalogue number A301-340A, 1:1,000); FANCL (Santa Cruz, catalogue number sc-137067, 1:500); and FANCD2 (abcam, catalogue number ab108928, 1:1,000).

### Growth curves

Indicated cell lines were grown for a total of 10 d in complete medium with the indicated concentrations of cisplatin, splitting every 2 d into fresh cisplatin as needed. Cells were counted every 2 d using a Countess 3 cell counter. Population doubling rates were determined using the following formula:

$$n=3.32\left({\mathrm{log}}\left(\frac{{X}_{{\rm{f}}}}{{X}_{{\rm{i}}}}\right)\right)$$

where *n* is population doubling, *X*_f_ the final cell count and *X*_i_ the initial cell count.

### Cell Titer Glo viability assay

The indicated cell lines were plated in white, opaque, 96-well plates optimized for luminescence assays (Greiner, catalogue number 655098) at the following concentrations: eHAP 150 cells per well; HT-1080 400 cells per well; NCIH-460 400 cells per well; A673 1,000 cells per well; HeLa Kyoto 400 cells per well; U2OS 500 cells per well; RPE-1 p53 KO 400 cells per well; HCT116 1,000 cells per well; DU145 800 cells per well; A549 600 cells per well; HEK293A 300 cells per well; and TOV112D 800 cells per well. Cells were then exposed to selected drugs at indicated concentrations and grown for a further 5 d without changing the medium (7 d for A673). To read luminescence activity, growth medium was removed and replaced with a 1:1 ratio of 90 μl of complete medium: 90 μl of Cell Titer Glo One Solution Assay reagent (Promega, catalogue number G8462). The 96-well plates were agitated for 5 min at 120 rpm, after which the luminescence readings were read using a BMG LabTech ClarioStar plate reader. Dose–response curves were generated by normalizing the luminescence reading of all samples to an untreated internal control for each cell line.

### Targeted CRISPR–Cas9 screening

Two customized sgRNA libraries containing four sgRNAs per gene were designed in house and ordered from Sigma-Aldrich (Supplementary Table 1). Pool 1 was designed to target genes found at human telomeres whereas pool 2 was designed as a supplemental sgRNA library to broaden the scope of pool 1 to cover additional targets involved in the DDR. Lentiviral particle preparations were obtained from Sigma-Aldrich and titered by infecting an equal number of eHAP iCas9 WT cells with increasing volumes of virus and determining the multiplicity of infection.

The eHAP iCas9 WT and PrimPol KO cells were infected with either pool 1 or pool 2 lentivirus in biological triplicate at a multiplicity of infection = 0.2 and an sgRNA representation of at least 500 cells per sgRNA. This representation was maintained throughout the rest of the screen. Cells harbouring the sgRNA libraries were enriched by selecting with puromycin for 2 d. Doxycycline was added to the medium to induce Cas9 expression over 6 d, splitting every 2 d with fresh doxycycline. Cells were then split into two arms per screen: untreated or treated with 450 nM cisplatin. Cells were grown for 6 d more, splitting every 2 d with fresh cisplatin in the treatment arm.

Cell pellets were collected throughout the screens with an appropriate number of cells to maintain 500 cells per sgRNA representation. Genomic DNA was isolated from these cells and subsequently used as templates for single-step PCR amplification of the sgRNA sequences present in each sample. PCR products were cleaned up using AMPure XP beads (Beckman Coulter, catalogue number A63800), according to the manufacturer’s instructions and quantified using a QuBit system. PCR products were deep sequenced using Illumina Sequencing.

Sequencing reads were trimmed, sgRNA counts were calculated (Supplementary Tables 3 and 4) and comparisons between samples were performed using the MaGeCK algorithm^50^ with default settings (Supplementary Table 5).

### Colony formation viability assay

The eHAP iCas9 cells were plated at a concentration of 200 cells per well in a 24-well plate in technical triplicates for each condition in each biological replicate. Cells were drugged with the indicated doses of cisplatin 18 h after plating. Colonies were allowed to form for 5 d after drugging, after which the medium was aspirated, colonies were washed with PBS, then stained with Crystal Violet stain (0.5% Crystal Violet in 20% methanol) for 15–30 min at room temperature. Excess Crystal Violet stain was removed and cells were washed with water and allowed to dry completely before imaging, using an Oxford Optronix GelCount colony counter. Colonies were quantified by automatically masking wells and adjusting CHARM settings to accurately detect colonies of varied sizes.

### Flow cytometry apoptosis assay

The eHAP iCas9 cells were seeded in 12-well plates and allowed to grow for 24 h before mock, cisplatin or GCN2 inhibitor treatment. After 40 h, cells were trypsinized and harvested in PBS, then transferred to 96-well round-bottomed plates (Falcon, catalogue number 353263). Cells were centrifuged at 500*g* for 5 min, after which the supernatant was aspirated. Cells were fixed in 100 μl of BD PhosFlow Fix Buffer I (BD Biosciences, catalogue number 557870) for 15 min at 37 °C. Cells were centrifuged again then permeabilized in 100 μl of BD PhosFlow Perm Buffer III (BD Biosciences, catalogue number 558050) at 4 °C for 30 min. Cells were centrifuged then blocked in 100 μl of blocking buffer (PBS + 10% goat serum) (Thermo Fisher Scientific, catalogue number G6767) for 30 min at room temperature. A 2× primary antibody dilution was made using rabbit anti-cleaved caspase-3 (BD Biosciences, catalogue number 570524) and added to blocked cells so that the final concentration of cleaved caspase-3 antibody was 1:1,000. After primary antibody incubation for 1 h, cells were washed once in PBS + 0.1% goat serum, then stained using goat anti-rabbit Alexa Fluor-647 (Thermo Fisher Scientific, catalogue number A-21244) at a final concentration of 1:1,000. After incubation with secondary antibody for 1 h at room temperature, cells were washed once in PBS + 0.1% goat serum, then resuspended in 200 μl of PBS + 0.1% goat serum. Cells were strained through a 35-μm mesh into round-bottomed flow cytometry tubes (Thermo Fisher Scientific, catalogue number 10585801). Doublets were removed from analysis by gating for forward scattering height versus area, after which cleaved caspase-3-positive cells were quantified.

### High-content immunofluorescence

The eHAP iCas9 cells were plated at a concentration of 3,000 cells per well in a black, 96-well PerkinElmer (now Revvity) PhenoPlate. Cells were allowed to attach overnight, after which they were mock treated or treated with the indicated doses of cisplatin for 24 h. Medium was aspirated and cells were washed once in PBS. Chromatin fractions were obtained by pre-extracting soluble proteins at the same time as fixation with 4% paraformaldehyde in PBS + 0.2% Triton X-100 for 15 min at room temperature. Fixed and extracted cells were washed twice with PBS and stored at 4 °C until ready to be stained.

When possible, all biological replicates were stained at the same time. PBS was aspirated from the 96-well plates, after which the cells were permeabilized by treating with PBS + 0.2% Triton X-100 for 15 min at room temperature. Cells were washed once in PBS and then blocked in blocking buffer (PBS + 3% bovine serum albumin + 0.1% Triton X-100) for 1 h at room temperature. Blocking buffer was removed and primary antibodies were applied at the following concentrations in blocking buffer for 1 h at room temperature: RPA32 phospho-Ser33 (Bethyl, catalogue number A300-246A, 1:2,500); RAD51 (Millipore, catalogue number ABE257, 1:500); γH2AX phospho-Ser139 (Millipore clone JBW301, catalogue number 05-636, 1:2,500); and FANCD2 (abcam, catalogue number ab108928, 1:2,000). After incubation with primary antibodies, cells were washed 3× for at least 5 min with PBS + 0.1% Triton X-100. Secondary antibodies and DAPI were diluted in blocking buffer to final concentrations of 1:1,000 (secondary antibodies) and 0.5 μg ml^−1^ of (DAPI), then incubated with cells for 1 h at room temperature. Goat anti-mouse Alexa Fluor-488 (Invitrogen, catalogue number A-11029) and goat anti-rabbit Alexa Fluor-647 (Invitrogen, catalogue number A-21245) were used in all experiments. After incubation with secondary antibodies, cells were washed 3× with PBS + 0.1% Triton X-100 for at least 5 min each. Cells were then washed with PBS twice and stored in PBS at 4 °C until ready to image.

Stained plates were scanned using the Operetta CLS confocal high-content system (×40). Maximum intensity projections were used for phenotypic analysis on 17 fields of view (FOVs); within each FOV, 5 planes were taken within each *z*-stack. Cell nuclei were identified using Harmony 5.1 software, followed by foci identification in the relevant imaging channels. Foci intensities were calculated as the sum intensity of foci per cell.

### QIBC

The eHAP iCas9 WT, PrimPol KO or SLFN11 KO cells with indicated genotypes were plated at a concentration of 10,000 (cisplatin treatment for 24 h) or 15,000 (ST1926 treatment time course) cells per well in a black, 96-well PhenoPlate in the presence of 10 μM BrdU. Treatments were added to cells as described in each figure. After completion of each treatment, cells were washed with PBS once. Chromatin-bound proteins were pre-extracted on ice in CSK buffer for 2 min. Cells were washed with PBS, then fixed in 4% paraformaldehyde in PBS for 15 min, washed again and stored in PBS until staining.

When possible, biological replicates for experiments were stained together. Cells were permeabilised in PBS + 0.2% Triton X-100 for 10 min and then blocked in blocking buffer (PBS + 3% BSA + 0.01% Triton X-100) for 45 min at room temperature. Primary antibodies targeting BrdU (mouse, BD Biosciences, catalogue number 555627, 1:250), RPA32 (rat, CST, catalogue number 2208 1:500) and either ATF4 (rabbit, CST, catalogue number 11815, 1:500) or γH2AX–pSer139 (rabbit, CST, catalogue number 2577, 1:500) were diluted at the indicated concentrations in blocking buffer and incubated with cells for 1 h at room temperature. Cells were washed 3× in PBS + 0.01% Triton X-100, after which secondary antibodies (goat anti-rat Alexa Fluor-647: Thermo Fisher Scientific, catalogue number A-21247, 1:1,000; HCA goat anti-mouse Alexa Fluor-488: Thermo Fisher Scientific, catalogue number A-11029, 1:1,000; HCA donkey anti-rabbit Alexa Fluor-568: Thermo Fisher Scientific, catalogue number A10042, 1:1,000) and DAPI (0.5 μg ml^−1^) were incubated with cells for 1 h at room temperature in the dark. Cells were washed twice with PBS + 0.01% Triton X-100 and once in PBS before imaging.

Stained plates were scanned using the Operetta CLS confocal high-content system (×20). Maximum intensity projections were used for phenotypic analysis on 49 FOVs. Within each FOV, five planes were taken within each *z*-stack. Cell nuclei were identified by DAPI staining using Harmony 5.1 software, followed by quantification of nuclear sum and mean intensities for each staining.

Raw mean BrdU–γH2AX–pSer139 and RPA32 intensities were plotted against one another in R Studio on log_2_(transformed) axes. Linear relationships between BrdU and RPA32 signals was derived from a linear transformation of untreated cells. RPA-exhausted cells were quantified by observing at which intensities the signal began to deviate from linearity. For experiments containing ATF4 staining, histograms of mean ATF4 nuclear intensities were generated for each condition. A threshold for all ATF4^+^ cells was drawn for each condition, after which the top quartile (25%) of ATF4^+^ cells was scored as ATF4^high^.

### Statistics and reproducibility

All statistical tests were performed using GraphPad Prism v.10.6.1 (799). The number of biological replicates and statistical test utilized for each figure panel are available in the corresponding figure legend. No statistical method was used to predetermine sample size. No data were excluded from the analyses. The experiments were not randomized and the investigators were not blinded to allocation during experiments and outcome assessment.

### Reporting summary

Further information on research design is available in the Nature Portfolio Reporting Summary linked to this article.

## Online content

Any methods, additional references, Nature Portfolio reporting summaries, source data, extended data, supplementary information, acknowledgements, peer review information; details of author contributions and competing interests; and statements of data and code availability are available at 10.1038/s41556-025-01852-1.

## Supplementary information

Reporting Summary

Supplementary Tables Supplementary Table 1 SgRNA library sequences. Supplementary Table 2 Oligonucleotide sequences. Supplementary Table 3 CRISPR screen, pool 1, raw counts. Supplementary Table 4 CRISPR screen, pool 2, raw counts. Supplementary Table 5 MaGeCK gene summaries.

## Source data

Source Data Figs. 1–7 and Extended Data Figs. 1, 2, 4 and 8 Unprocessed western blots.

Source Data Figs. 1–7 and Extended Data Figs. 1–7 and 9 Statistical source data.

## Data Availability

Raw Illumina sequencing FASTQ files related to the CRISPR–Cas9 screens performed in this study are publicly available in the National Center for Biotechnology Information Sequence Read Archive and BioProject Databases with accession number PRJNA1214387. Source data are provided with this paper.
