# Supplementary material for: RPA exhaustion activates SLFN11 to eliminate cells with heightened replication stress
Source: Nat Cell Biol. 2026 Jan 9;28(2):240–54. doi: 10.1038/s41556-025-01852-1 (PMC12904793; doi:10.1038/s41556-025-01852-1)
Supplement: Supplementary file 1 — Reporting Summary [file 41556_2025_1852_MOESM1_ESM.pdf]

nature portfolio

Corresponding author(s): Simon J. Boulton

Last updated by author(s): Nov 18, 2025

Reporting Summary

Nature Portfolio wishes to improve the reproducibility of the work that we publish. This form provides structure for consistency and transparency in reporting. For further information on Nature Portfolio policies, see our Editorial Policies and the Editorial Policy Checklist.

Statistics

For all statistical analyses, confirm that the following items are present in the figure legend, table legend, main text, or Methods section.

n/a	Confirmed	
☐	☒	The exact sample size (*n*) for each experimental group/condition, given as a discrete number and unit of measurement
☐	☒	A statement on whether measurements were taken from distinct samples or whether the same sample was measured repeatedly
☐	☒	The statistical test(s) used AND whether they are one- or two-sided<br>*Only common tests should be described solely by name; describe more complex techniques in the Methods section.*
☒	☐	A description of all covariates tested
☒	☐	A description of any assumptions or corrections, such as tests of normality and adjustment for multiple comparisons
☐	☒	A full description of the statistical parameters including central tendency (e.g. means) or other basic estimates (e.g. regression coefficient) AND variation (e.g. standard deviation) or associated estimates of uncertainty (e.g. confidence intervals)
☐	☒	For null hypothesis testing, the test statistic (e.g. *F*, *t*, *r*) with confidence intervals, effect sizes, degrees of freedom and *P* value noted<br>*Give P values as exact values whenever suitable.*
☒	☐	For Bayesian analysis, information on the choice of priors and Markov chain Monte Carlo settings
☒	☐	For hierarchical and complex designs, identification of the appropriate level for tests and full reporting of outcomes
☒	☐	Estimates of effect sizes (e.g. Cohen's *d*, Pearson's *r*), indicating how they were calculated

Our web collection on statistics for biologists contains articles on many of the points above.

Software and code

Policy information about availability of computer code

Data collection	Cell Titer Glo: BMG Labtech Clariostar v5.70R2; Western blotting: Biorad Chemidoc v3.0.1.14; Colony formation assays: Oxford Optronix Gelcount v1.2; Flow cytometry: FACSDiva v10; High Content Immunofluorescence: Revvity Harmony v5.1.
Data analysis	Statistics and graphing: Prism Graphpad v10.6; Flow cytometry: FlowJo v10; Colony formation assays: Oxford Optronix Gelcount v1.2; High content immunofluorescence: Revvity Harmony v5.1; CRISPR screening: MaGeCK v0.5.9, Data visualisation and calculations: R Studio 2024.04.1+748

For manuscripts utilizing custom algorithms or software that are central to the research but not yet described in published literature, software must be made available to editors and reviewers. We strongly encourage code deposition in a community repository (e.g. GitHub). See the Nature Portfolio guidelines for submitting code & software for further information.

Data

Policy information about availability of data

All manuscripts must include a data availability statement. This statement should provide the following information, where applicable:

- Accession codes, unique identifiers, or web links for publicly available datasets
- A description of any restrictions on data availability
- For clinical datasets or third party data, please ensure that the statement adheres to our policy

Raw Illumina sequencing reads related to the CRISPR-Cas9 screens performed in this study are publicly available in the NCBI Sequence Read Archive and BioProject Databases with accession number PRJNA1214387. Source numerical statistics data and unprocessed raw Western blots are provided with this study.

Research involving human participants, their data, or biological material

Policy information about studies with human participants or human data. See also policy information about sex, gender (identity/presentation), and sexual orientation and race, ethnicity and racism.

Reporting on sex and gender	N/A
Reporting on race, ethnicity, or other socially relevant groupings	N/A
Population characteristics	N/A
Recruitment	N/A
Ethics oversight	N/A

Note that full information on the approval of the study protocol must also be provided in the manuscript.

Field-specific reporting

Please select the one below that is the best fit for your research. If you are not sure, read the appropriate sections before making your selection.

☒ Life sciences ☐ Behavioural & social sciences ☐ Ecological, evolutionary & environmental sciences

For a reference copy of the document with all sections, see nature.com/documents/nr-reporting-summary-flat.pdf

Life sciences study design

All studies must disclose on these points even when the disclosure is negative.

Sample size	No pre-determined sample sizes were utilised in this study. Experiments were always performed in biological duplicate (at a minimum). Sample sizes in immunofluorescence-based experiments were not pre-determined but were determined by capturing a set number of fields of view across multiple biological replicates.
Data exclusions	No data were excluded in these studies
Replication	All experiments were performed in at least biological duplicate at a minimum. No replicates were excluded in downstream analyses and all data were therefore reported. No issues arose with replication of experiments.
Randomization	No experiments were performed on organisms or participants. Randomization of samples was not relevant in this study as all experiments were conducted in an unbiased manner. Immunofluorescence-based experiments were performed using automated image acquisition as described in the materials and methods and thus was inherently unbiased.
Blinding	Blinding is not relevant to this study; drug sensitivities and cell viability assays were measured using an unbiased

Reporting for specific materials, systems and methods

We require information from authors about some types of materials, experimental systems and methods used in many studies. Here, indicate whether each material, system or method listed is relevant to your study. If you are not sure if a list item applies to your research, read the appropriate section before selecting a response.

Materials & experimental systems

n/a	Involved in the study
☐	☒ Antibodies
☐	☒ Eukaryotic cell lines
☒	☐ Palaeontology and archaeology
☒	☐ Animals and other organisms
☒	☐ Clinical data
☒	☐ Dual use research of concern
☒	☐ Plants

Methods

n/a	Involved in the study
☒	☐ ChIP-seq
☐	☒ Flow cytometry
☒	☐ MRI-based neuroimaging

Antibodies

Antibodies used: PRIMPOL (Murón et al 2013 and Proteintech 29824-1-AP); PCNA (Santa Cruz sc-56); vinculin (Sigma V9131); RPA32 pS33 (Bethyl A300-245A); RPA32 (abcam ab2175); Chk1 pS345 (Cell Signalling 9664S); Chk1 (Sigma C9358); SLFN11 (Santa Cruz sc-374339); pGCN2 T899 (abcam 75836); anti-cleaved caspase-3 (BD Biosciences #570524); GAPDH (Abcam [6C5] ab8245); USP1 (Bethyl A301-699A or Proteintech 14346-1-AP); WDR48 (Proteintech 16503-1-AP); PCNA Ub K164 (Cell Signalling 13439); SMC1 (abcam ab21583); RAD18 (Bethyl A301-340A); FANCL (Santa Cruz sc-137067); FANCD2 (abcam ab108928); RAD51 (Millipore ABE257); gamma H2AX phospho serine 139 (Millipore clone JBW301 05-636); BrdU (mouse BD Biosciences 555627), RPA32 (rat CST 2208), ATF4 (rabbit CST 11815) Goat anti-mouse Alexa Fluor 488 (Invitrogen A-11029); goat anti-rabbit Alexa Fluor 647 (Invitrogen A-21245) goat anti-rat 647 (ThermoFisher A-21247), HCA goat anti-mouse 488 (ThermoFisher A-11029); HCA donkey anti-rabbit 568 (ThermoFisher A10042).

Validation: All antibodies were validated by knockdown experiments, validation from supplier's catalog or by using the CiteAb database.

Eukaryotic cell lines

Policy information about cell lines and Sex and Gender in Research

Cell line source(s): No primary cell lines or cell lines derived from participants were utilised in this study. All cell lines in this study were sourced from the Cell Science facility at the Francis Crick Institute.

Authentication: Standard procedure in the Cell Science facility at the Francis Crick Institute is to authenticate cell lines by STR profiling; however, not all cell lines utilised in this study were authenticated.

Mycoplasma contamination: Standard procedure in the Cell Science facility at the Francis Crick Institute is to confirm all cell lines are free of mycoplasma contamination - all cell lines in this study are mycoplasma negative.

Commonly misidentified lines (See ICLAC register): No misidentified cell lines were utilised in this study.

Plants

Seed stocks: No plants were used in this study.

Novel plant genotypes: No plants were used in this study.

Authentication: No plants were used in this study.

Flow Cytometry

Plots

Confirm that:

☒ The axis labels state the marker and fluorochrome used (e.g. CD4-FITC).

☒ The axis scales are clearly visible. Include numbers along axes only for bottom left plot of group (a 'group' is an analysis of identical markers).

☒ All plots are contour plots with outliers or pseudocolor plots.

☒ A numerical value for number of cells or percentage (with statistics) is provided.

Methodology

Sample preparation	Cell lines were tested for Cas9 activity as described in the materials and methods section. Cells were trypsinised and diluted in PBS and run natively on the flow cytometers OR fixed and stained as detailed in the materials and methods
Instrument	BD Biosciences LSRFortessa
Software	BD FACSDiva was used to collect data and FlowJo was used to analyse the data
Cell population abundance	Cells were gated for those expressing either BFP or mCherry as indicative of infected cells. Within the BFP or mCherry population, GFP negative and positive cells were gated and measured as a percentage of total BFP/mCherry-positive population. For cleaved-caspase-3 cells, cells were gated for single cells and at least 10,000 cells were collected per replicate and per condition.
Gating strategy	Cells were gated using SSC-Area vs. FSC-Area, followed by doublet elimination by gating FSC-Width vs. FSC-Area. BFP or mCherry-positive cells were gated by plotting BFP/mCherry vs. FSC-Area and centering BFP/mCherry-negative cells around 10^2. BFP or mCherry-positive cells were gated based upon significant BFP or mCherry signal (greater than 10^4). Within this population, BFP or mCherry fluorescence was plotted against GFP fluorescence, and GFP-negative cells were centered around 10^2 and positive cells were gated above 10^4 fluorescence intensity. For cleaved caspase-3 staining, single cells were gated as with BFP/mCherry iCas9 assays. CC3 negative cells were centred around 10^2 intensity with all cells above 10^3 intensity were gated as CC3+.

☒ Tick this box to confirm that a figure exemplifying the gating strategy is provided in the Supplementary Information.
