# Supplementary material for: RPA exhaustion activates SLFN11 to eliminate cells with heightened replication stress
Source: Nat Cell Biol. 2026 Jan 9;28(2):240–54. doi: 10.1038/s41556-025-01852-1 (PMC12904793; doi:10.1038/s41556-025-01852-1)

Figure 1d - PRIMPOL

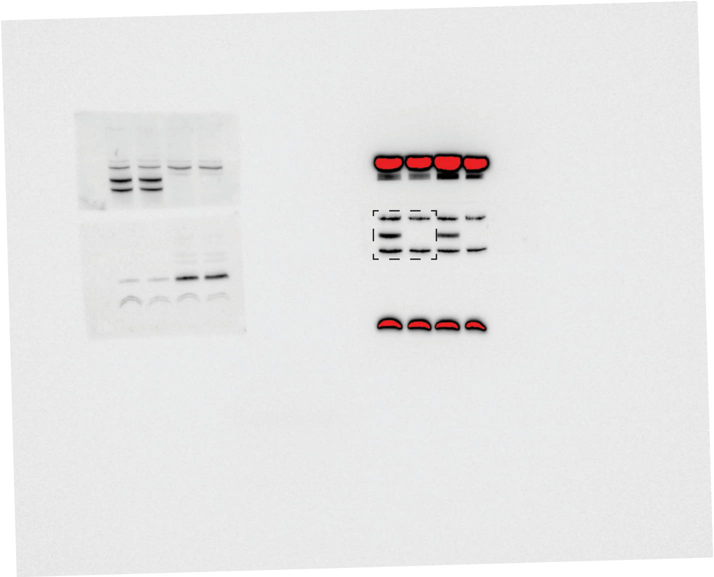

Figure 1d - PCNA

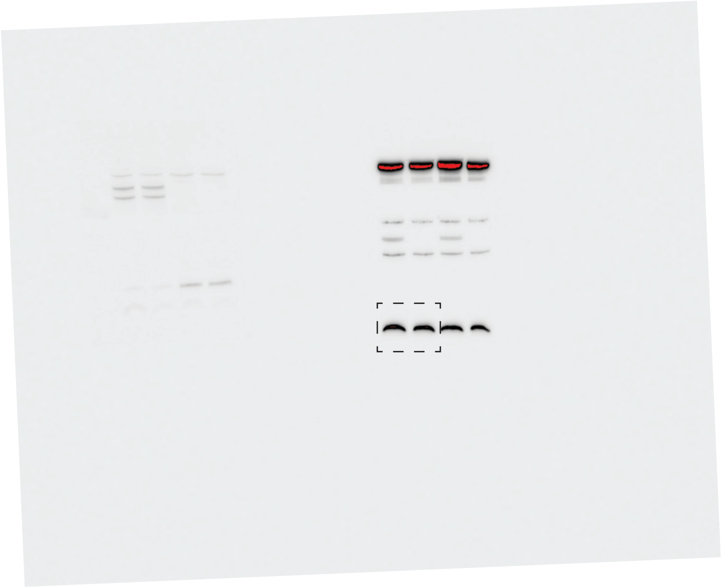

Figure 1g - PRIMPOL

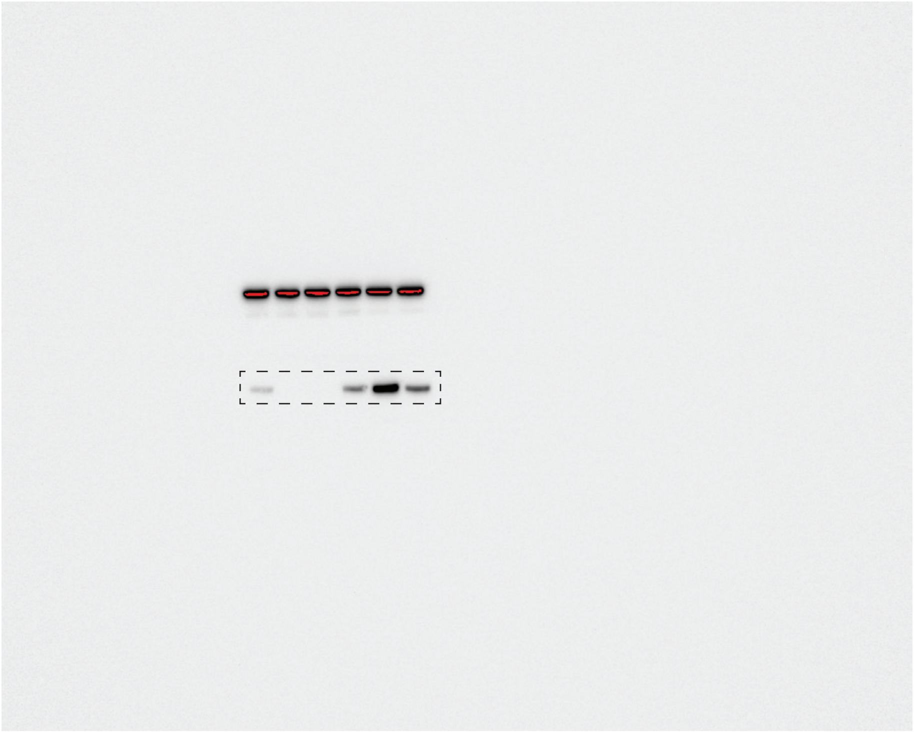

Figure 1g - vinculin

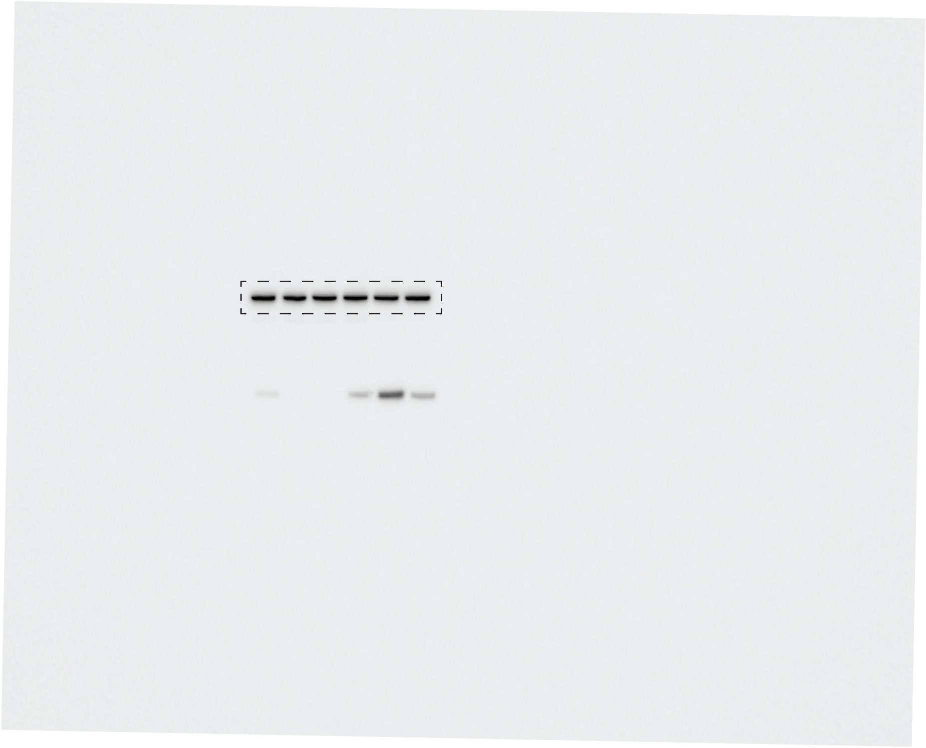

Figure 2d - PRIMPOL

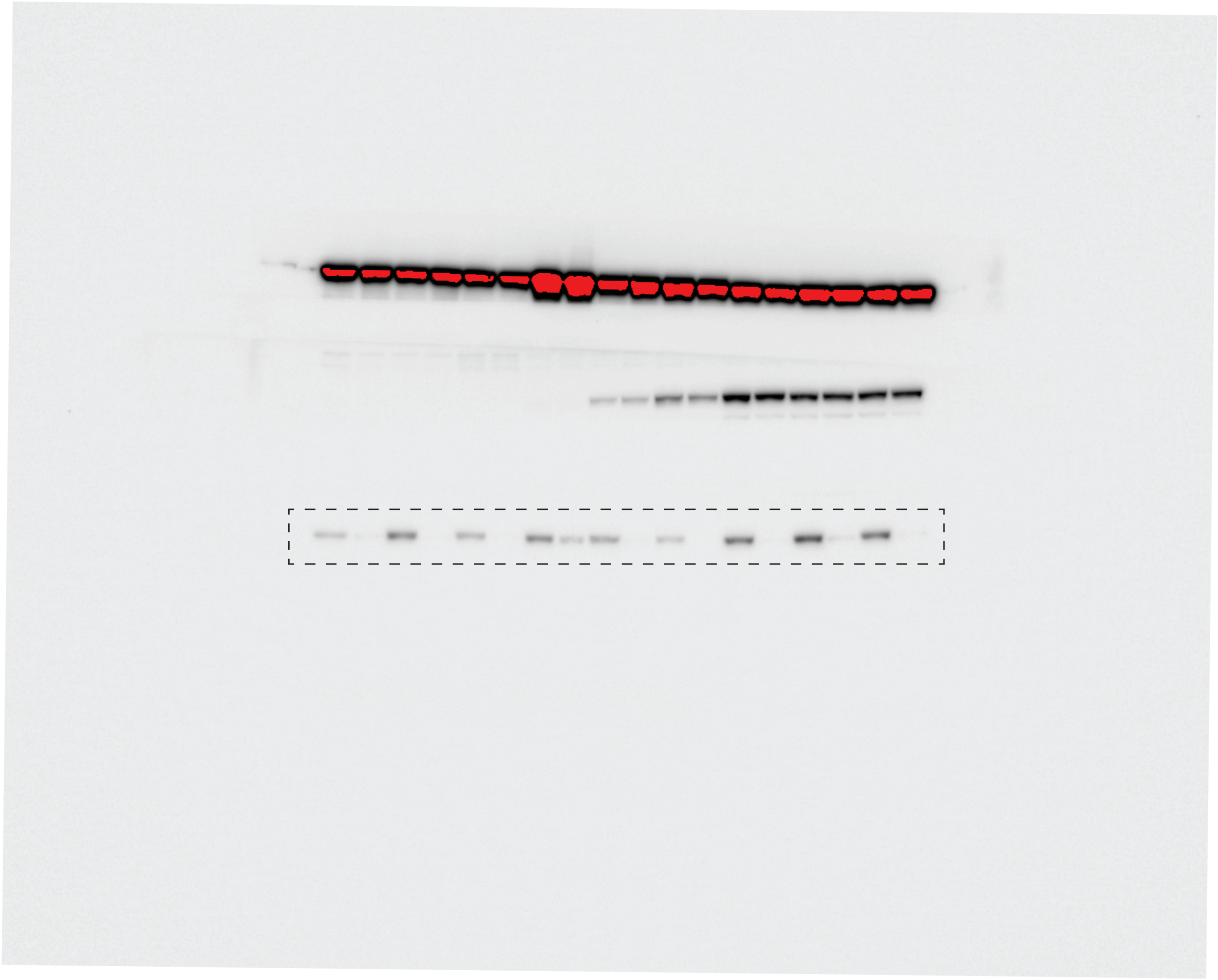

Figure 2d - SLFN11

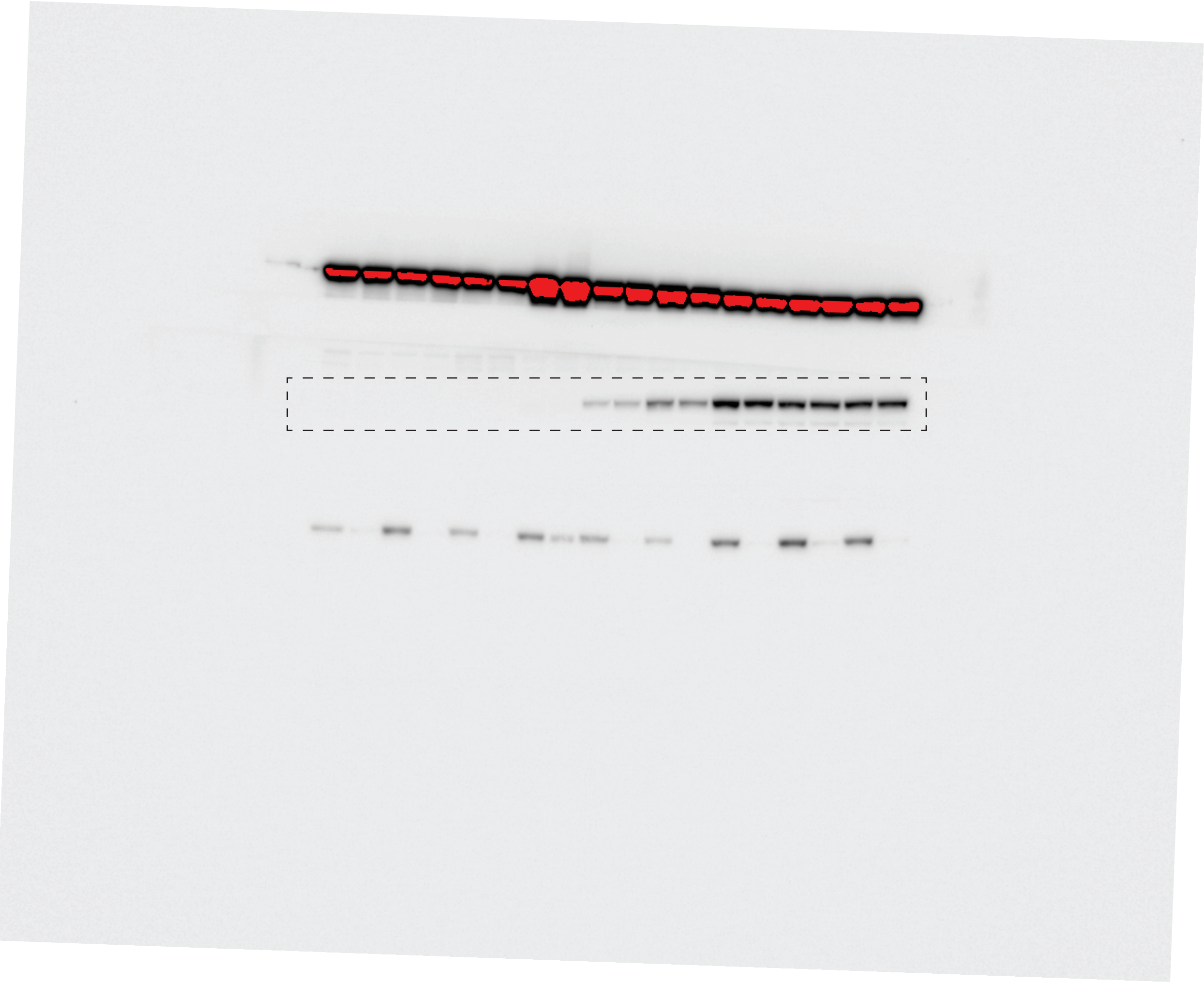

Figure 2d - vinculin

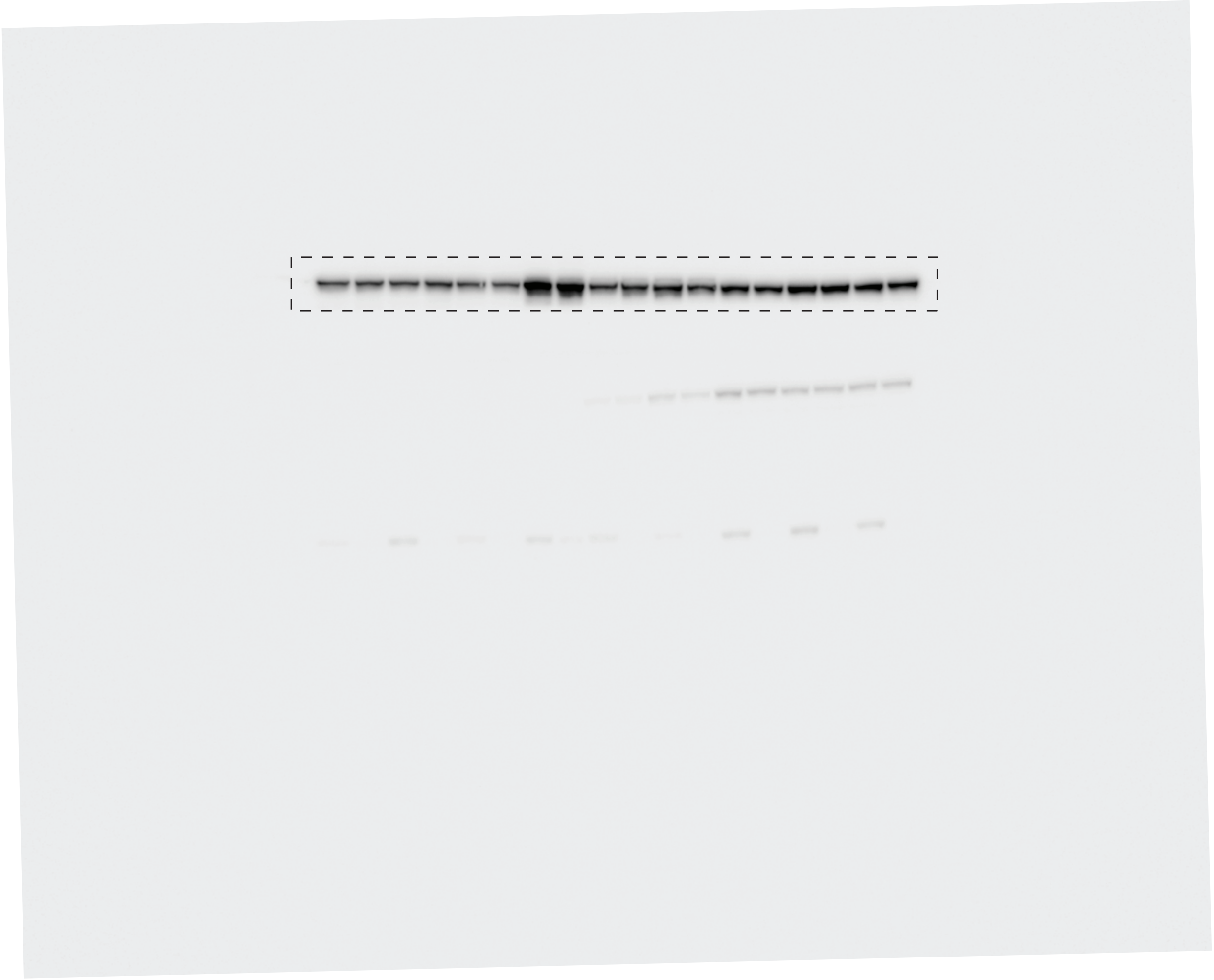

Figure 2d - ponceau

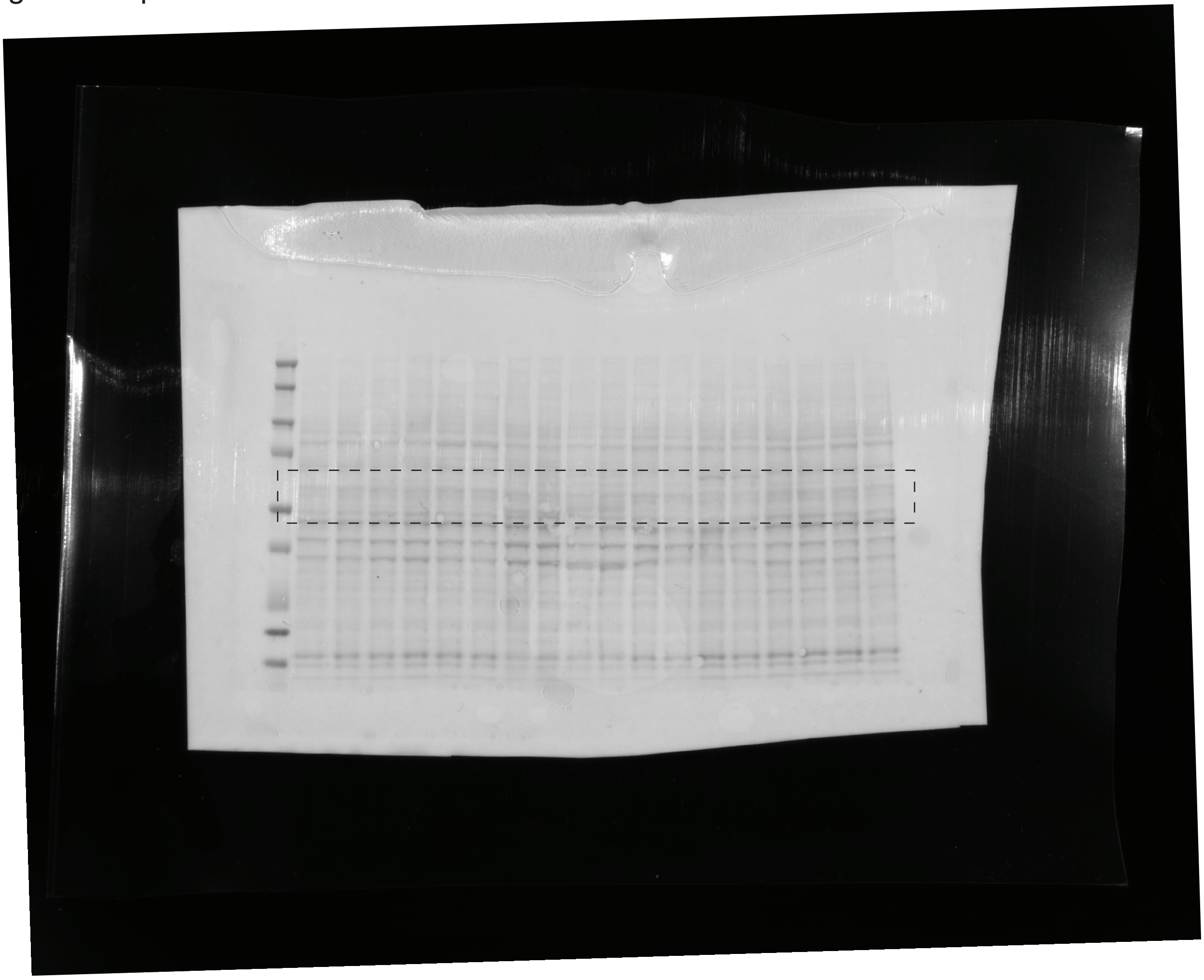

Figure 3b - PRIMPOL

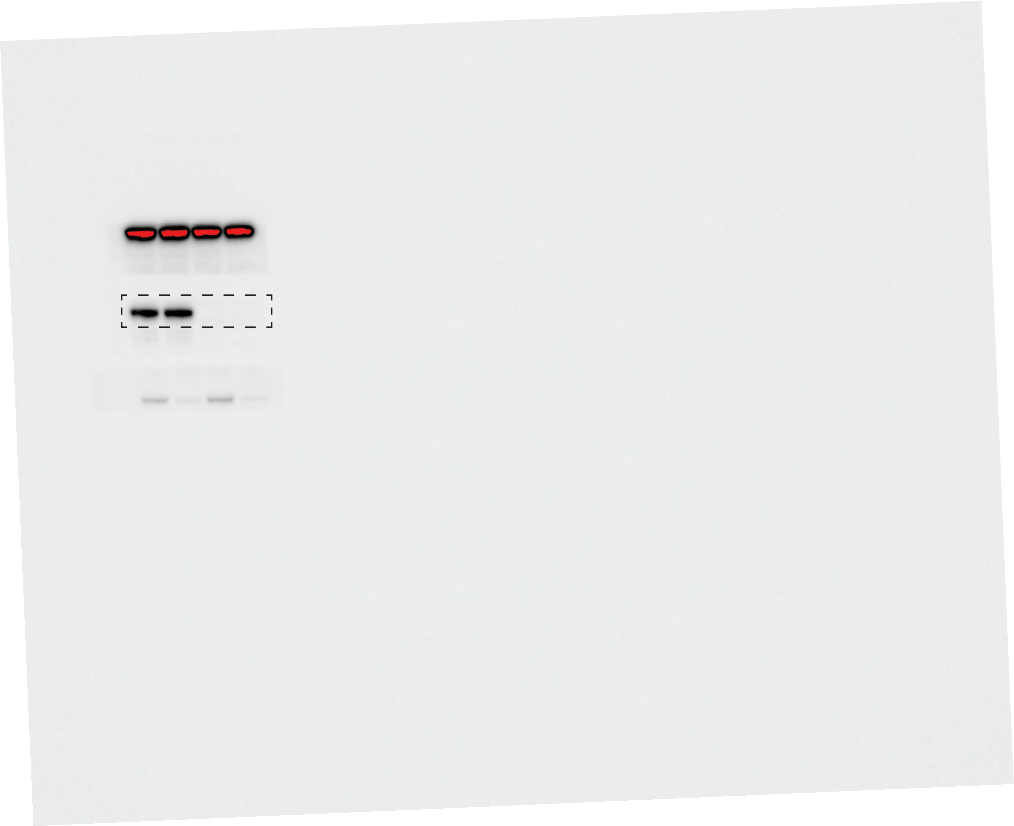

Figure 3b - SLFN11

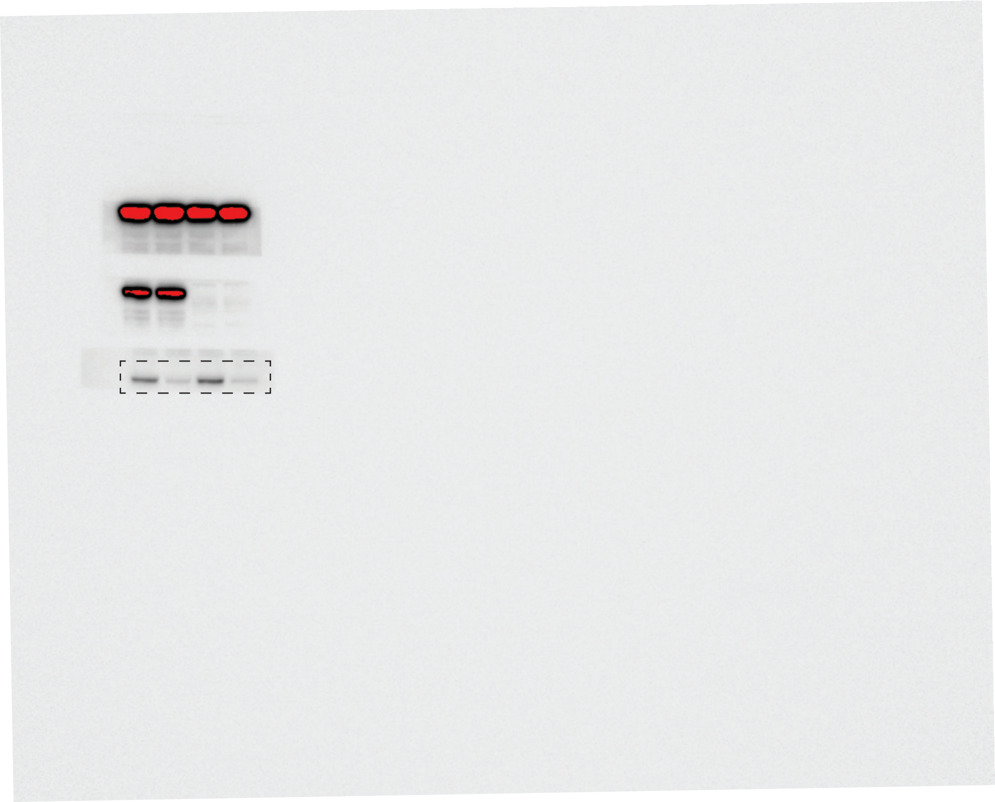

Figure 3b - vinculin

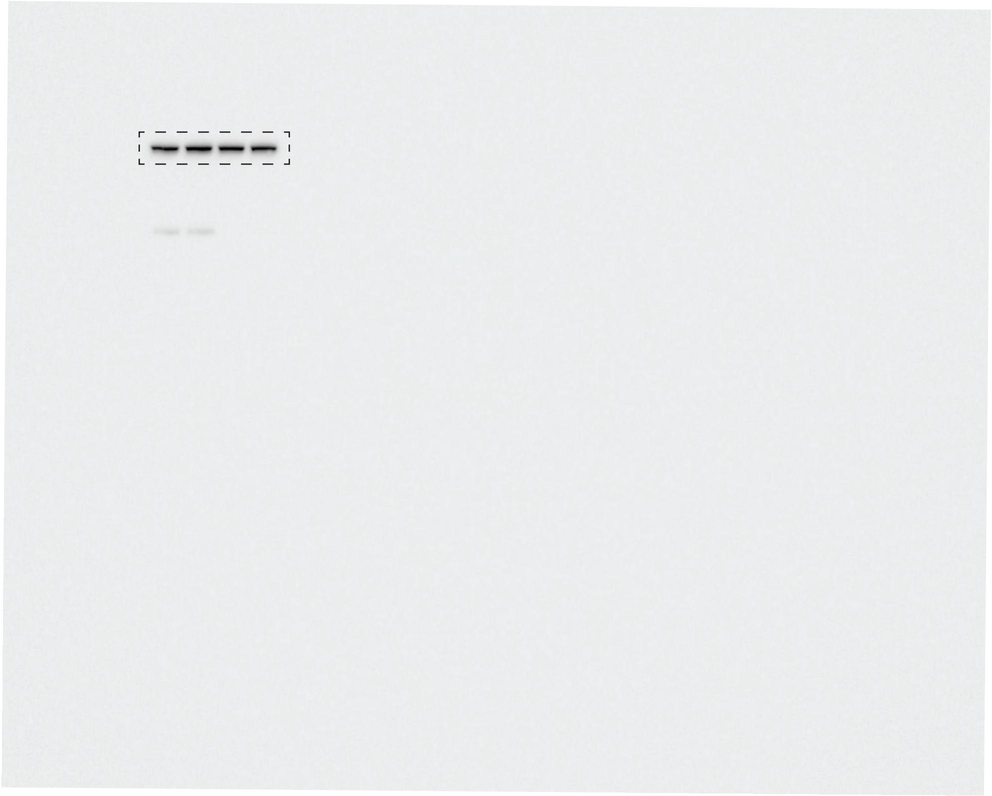

Figure 3b - ponceau

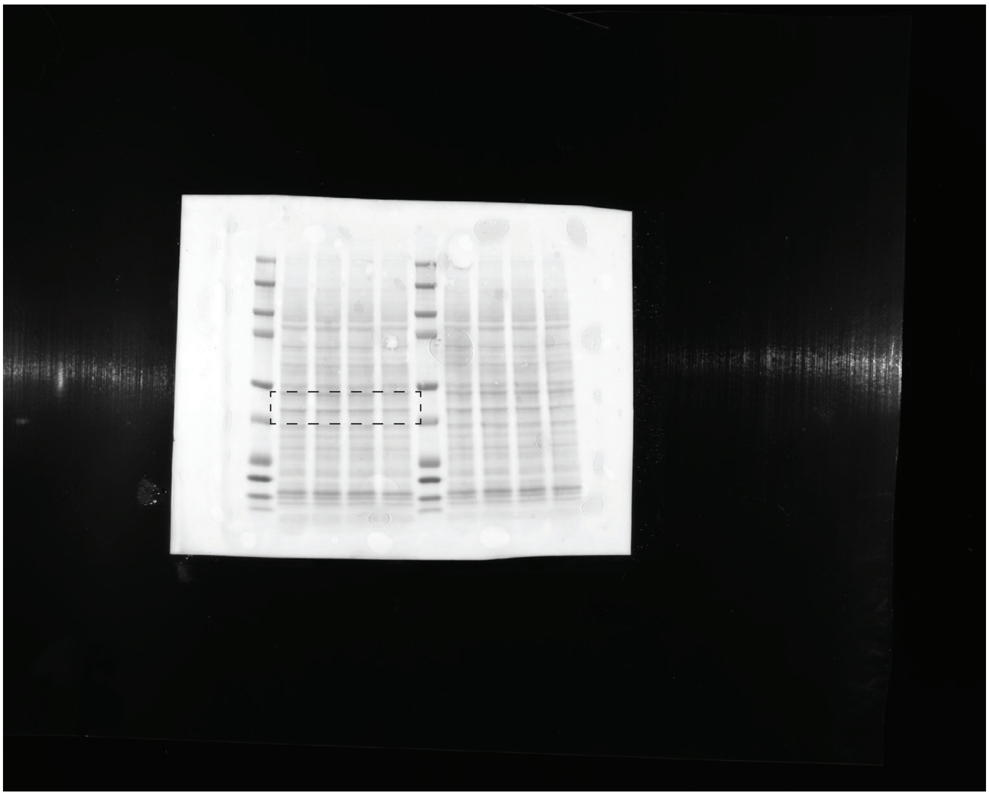

Figure 3j - SLFN11

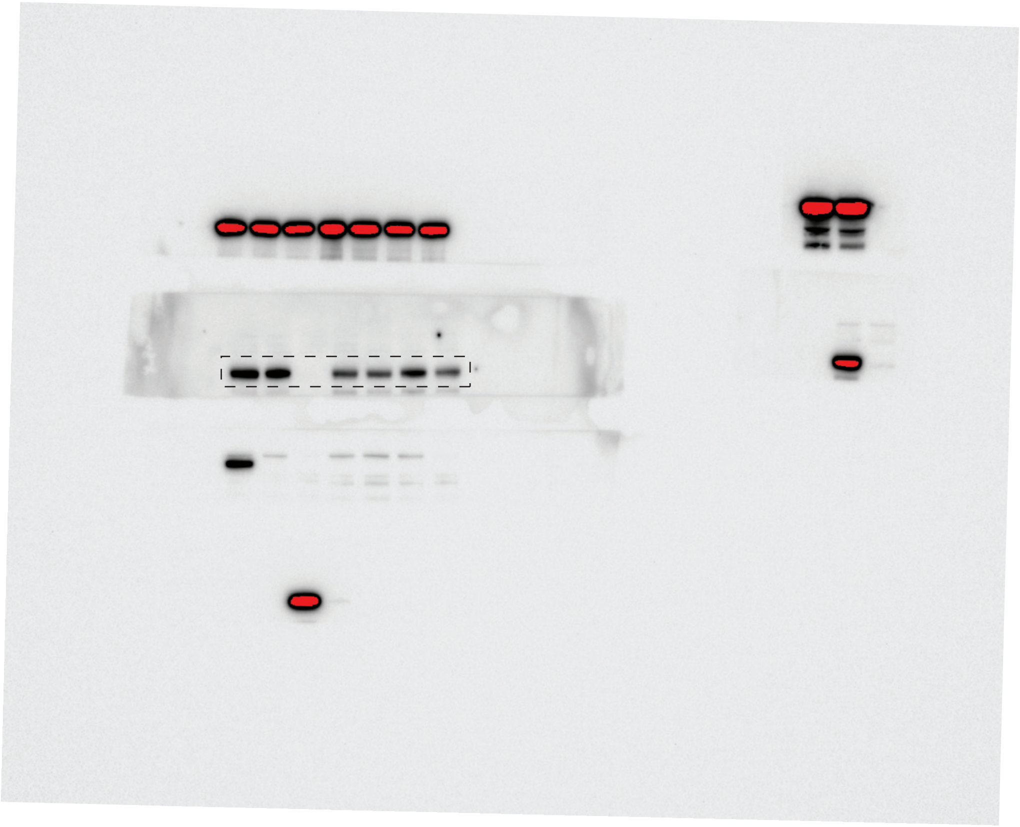

Figure 3j - PRIMPOL

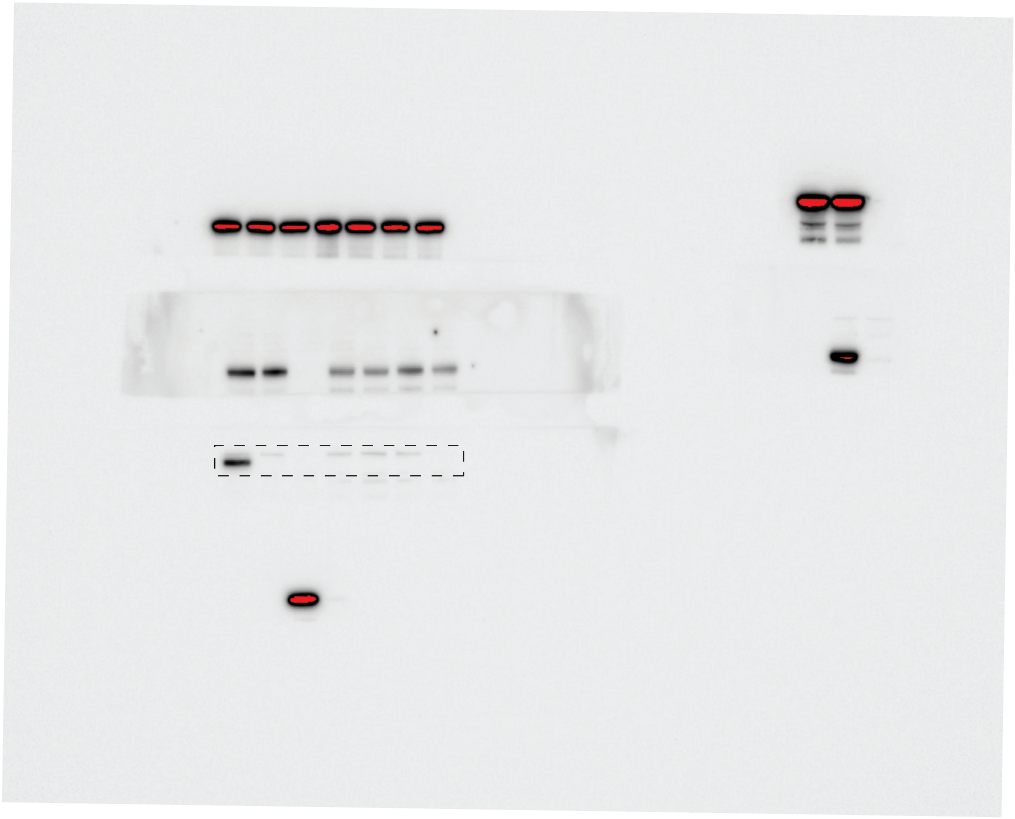

Figure 3j - GFP

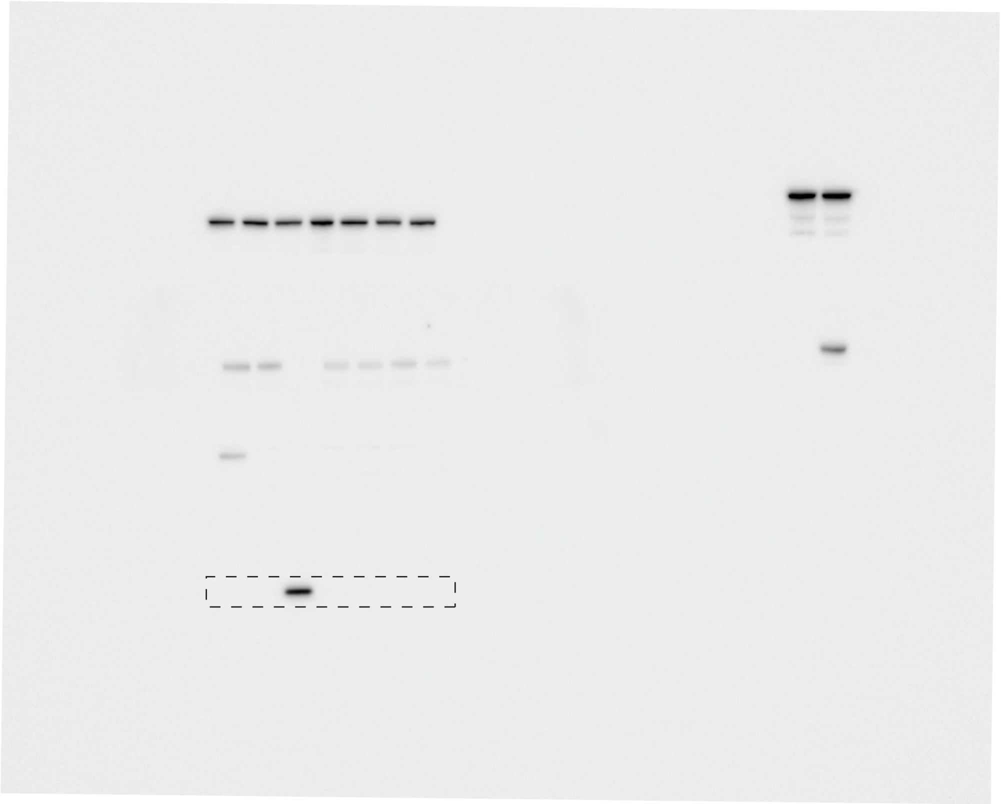

Figure 3j - vinculin

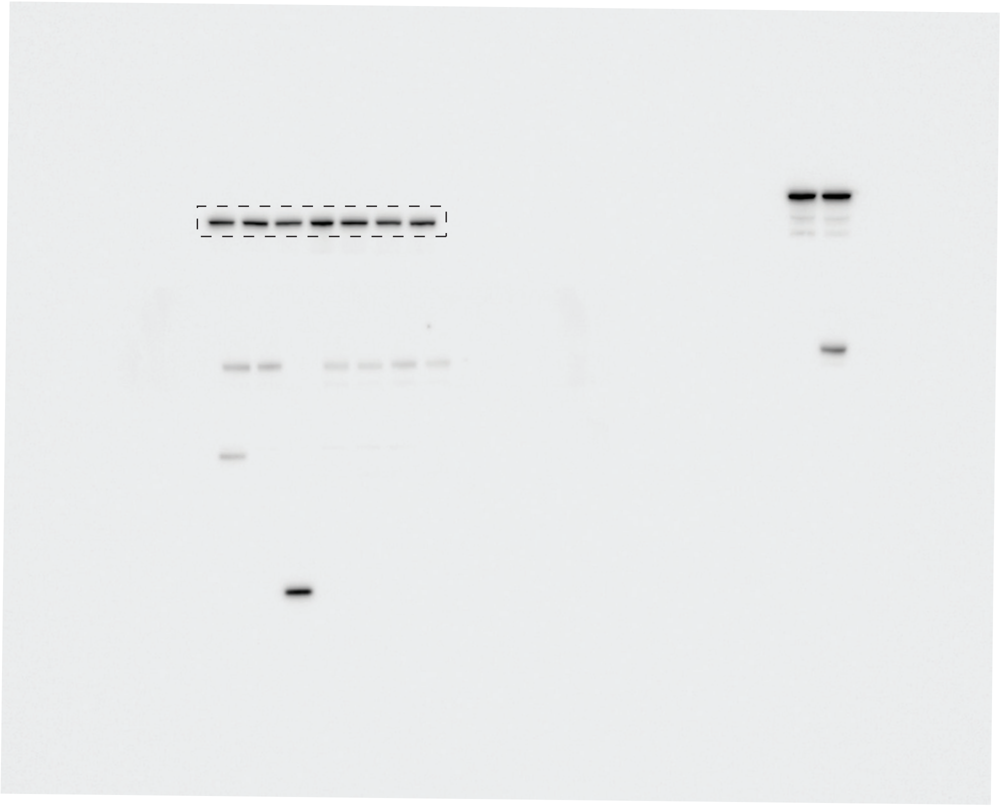

Figure 3o - pGCN2 T899

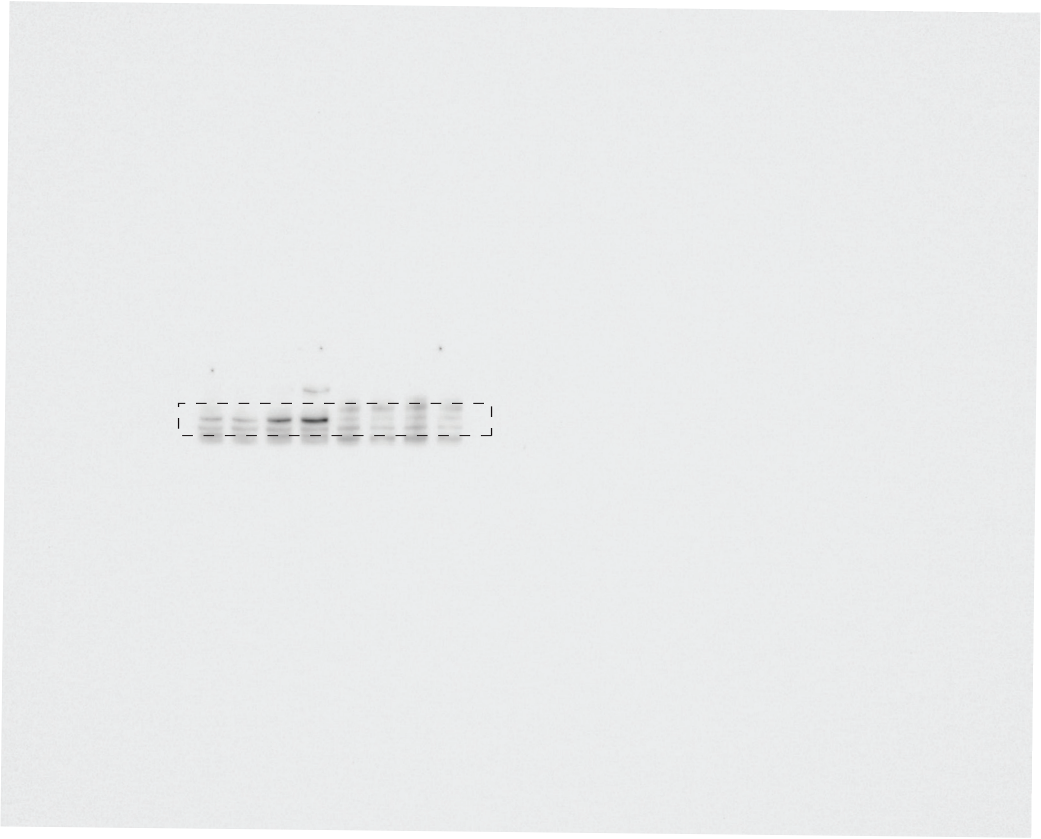

Figure 3o - SLFN11 (s.e.)

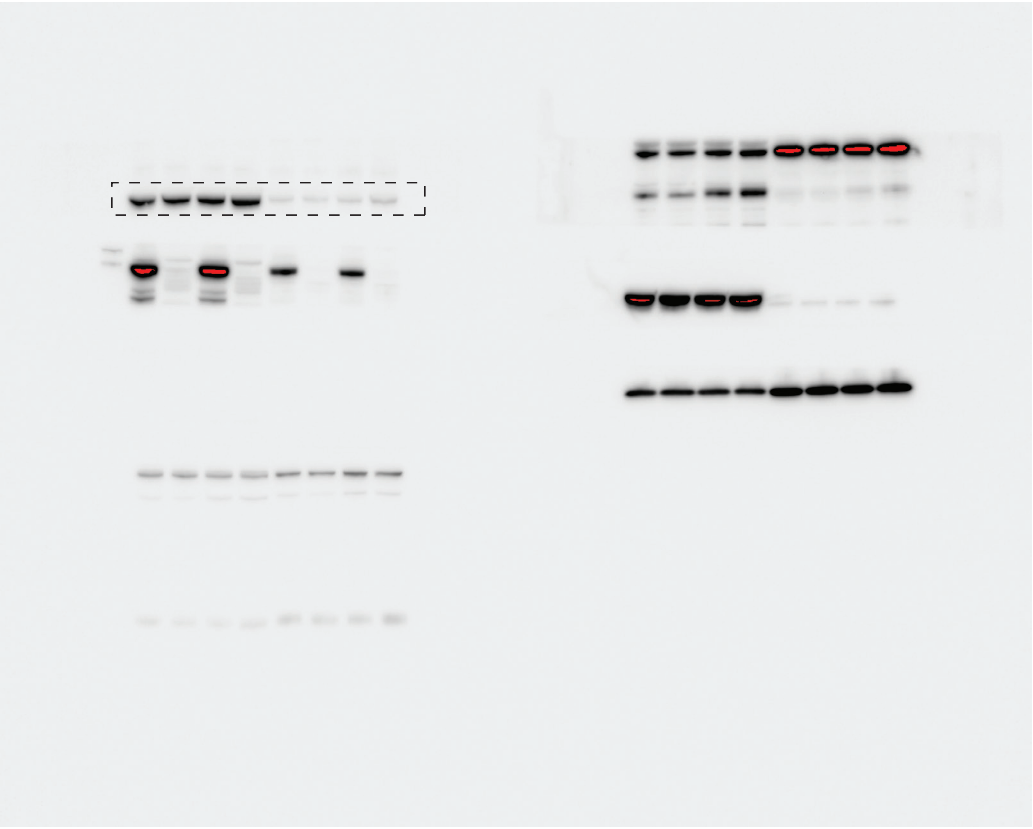

Figure 3o - SLFN11 (l.e.)

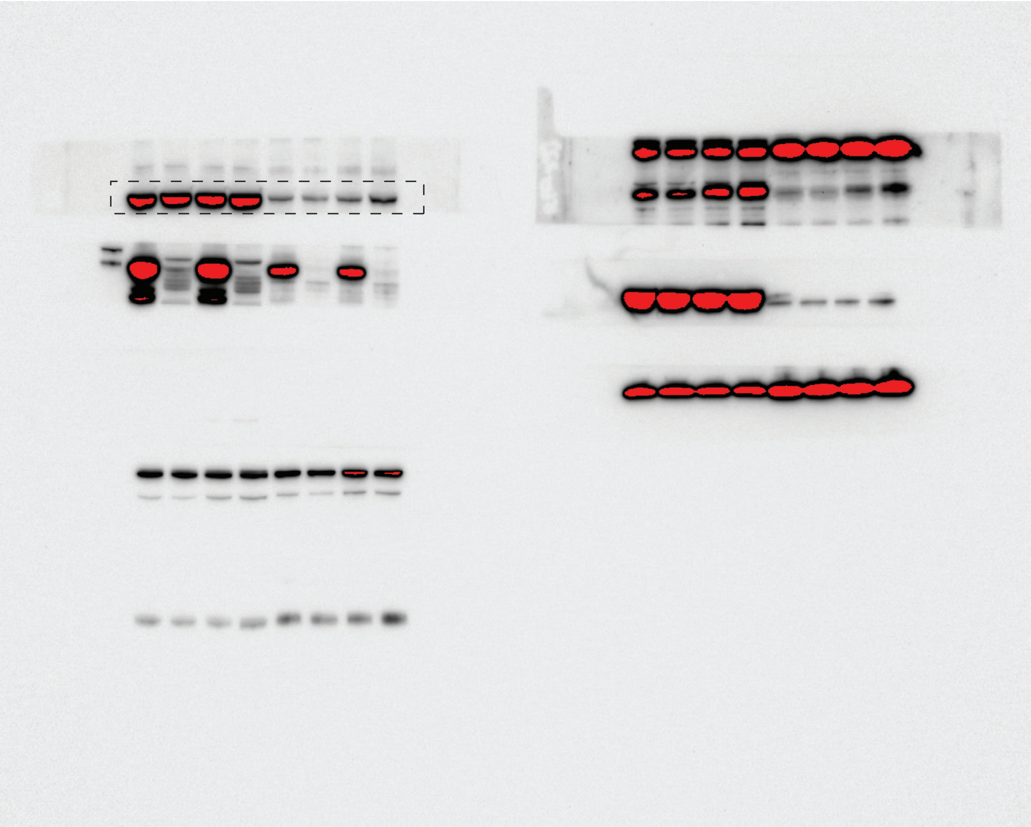

Figure 3o - RPA pS33

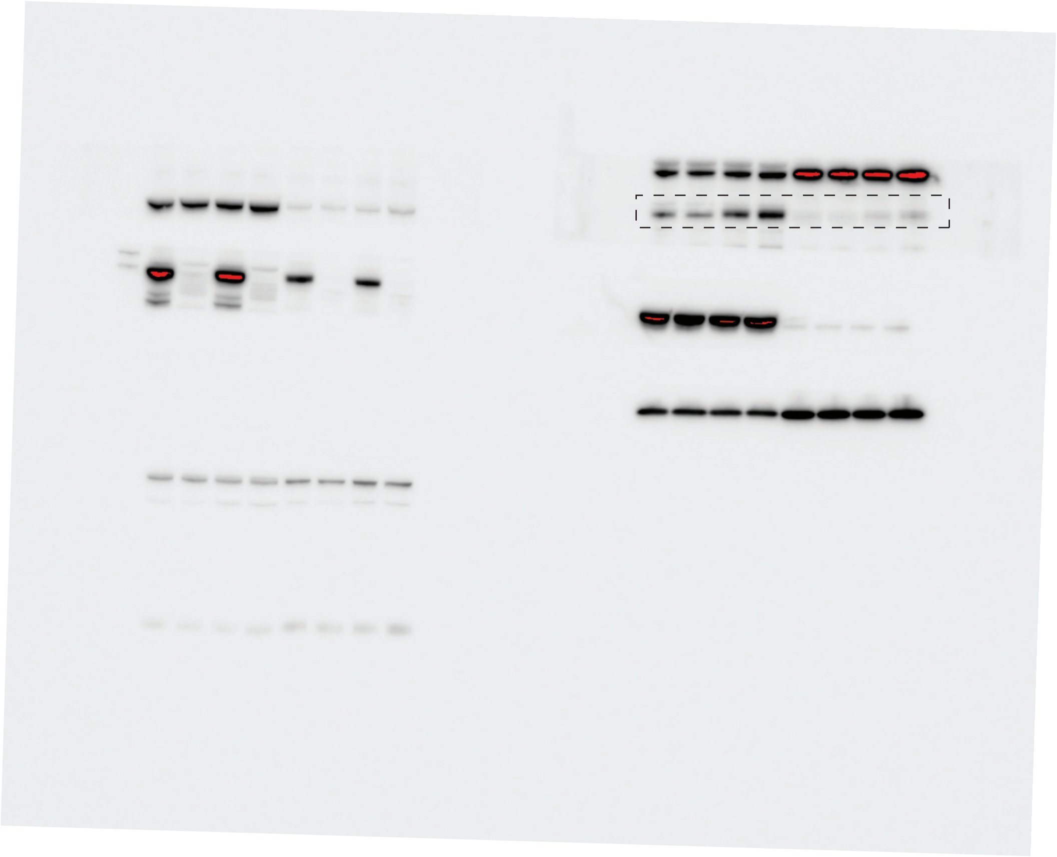

Figure 3o - PRIMPOL

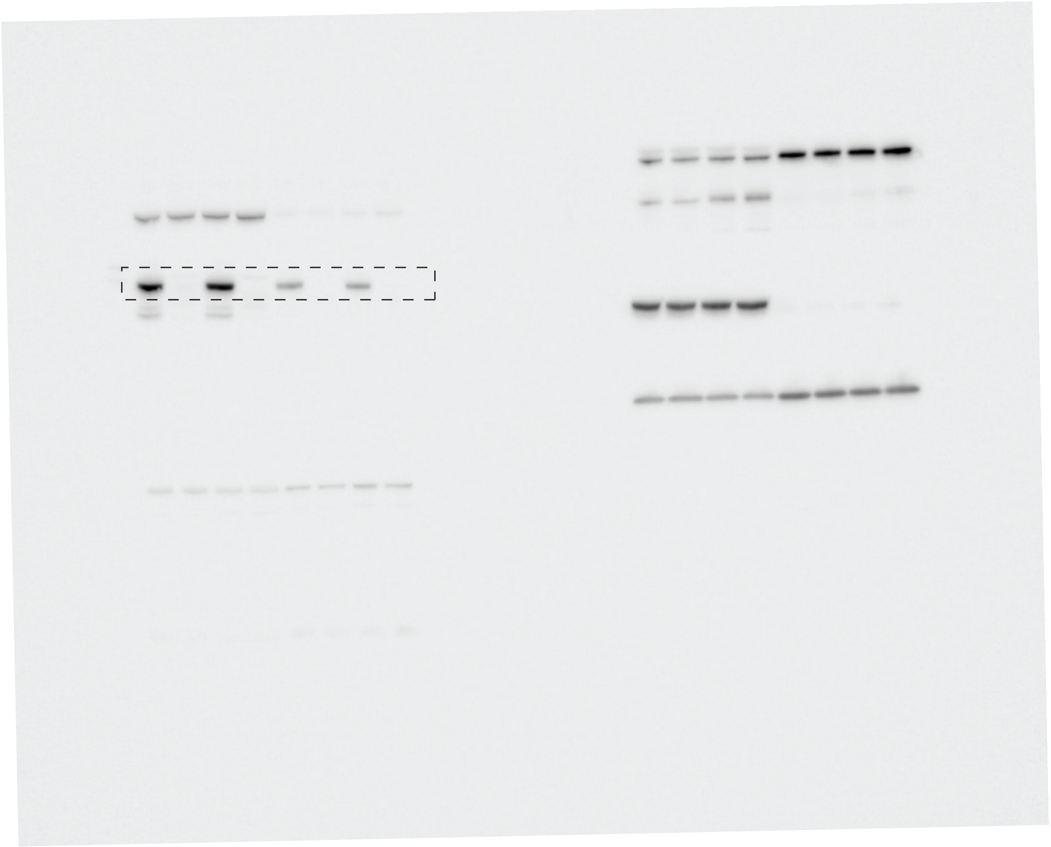

Figure 3o - tubulin

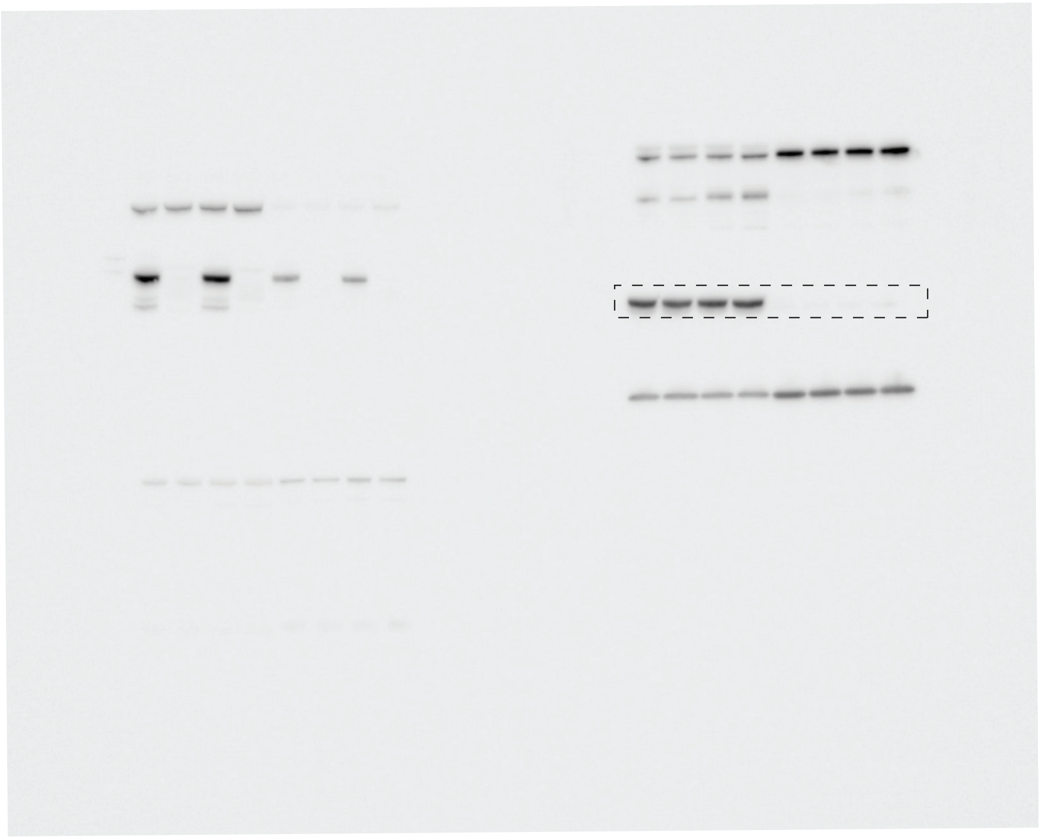

Figure 3o - histone H3

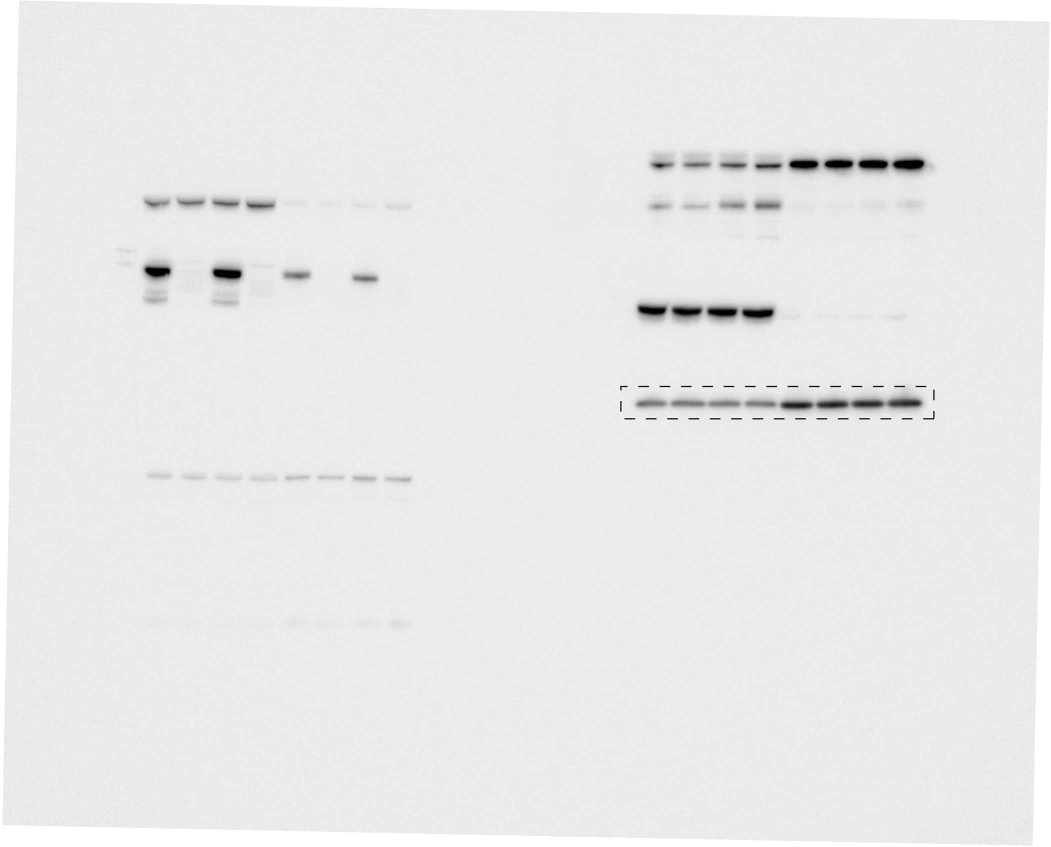

Figure 4h - RPA2

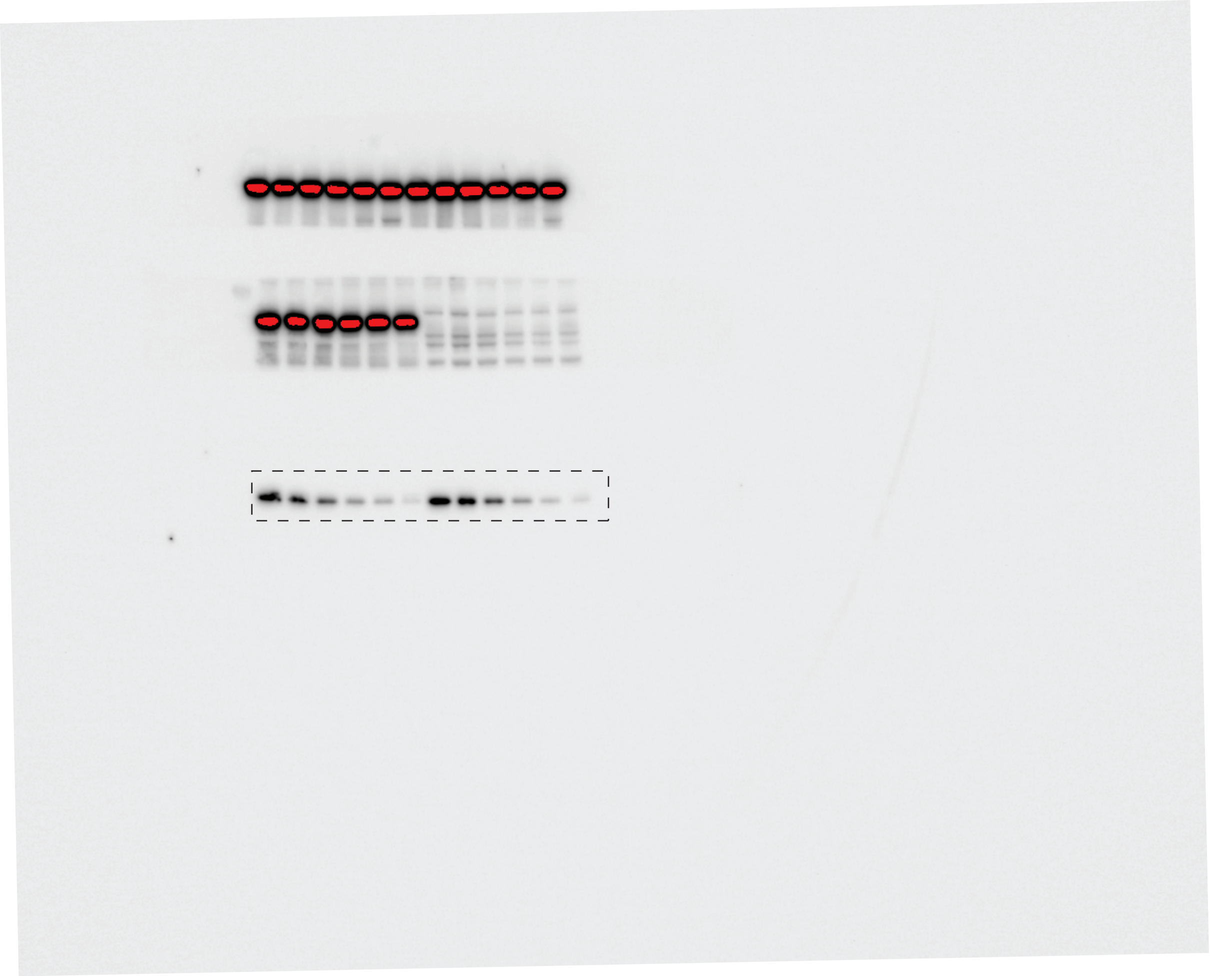

Figure 4h - PRIMPOL

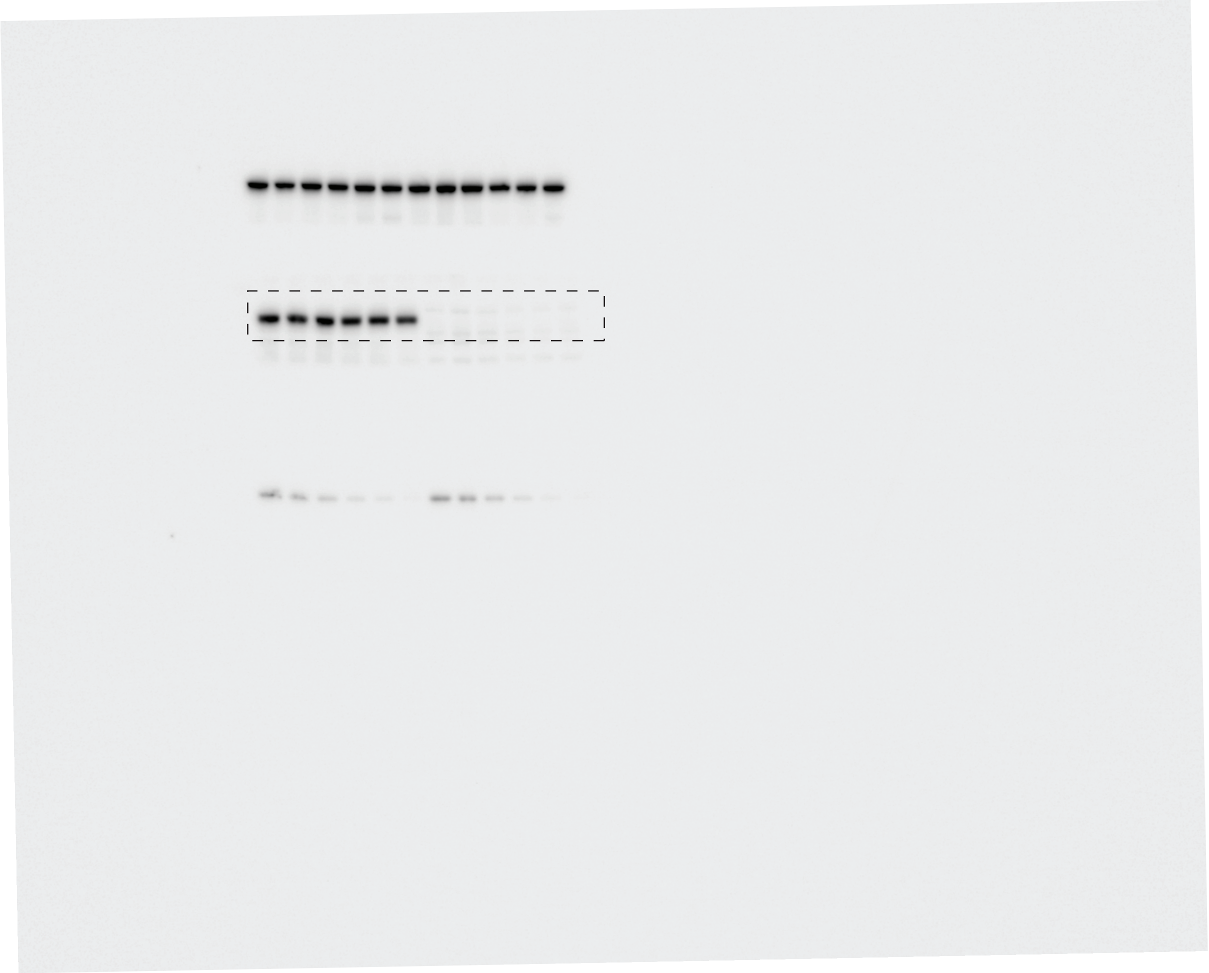

Figure 4h - vinculin

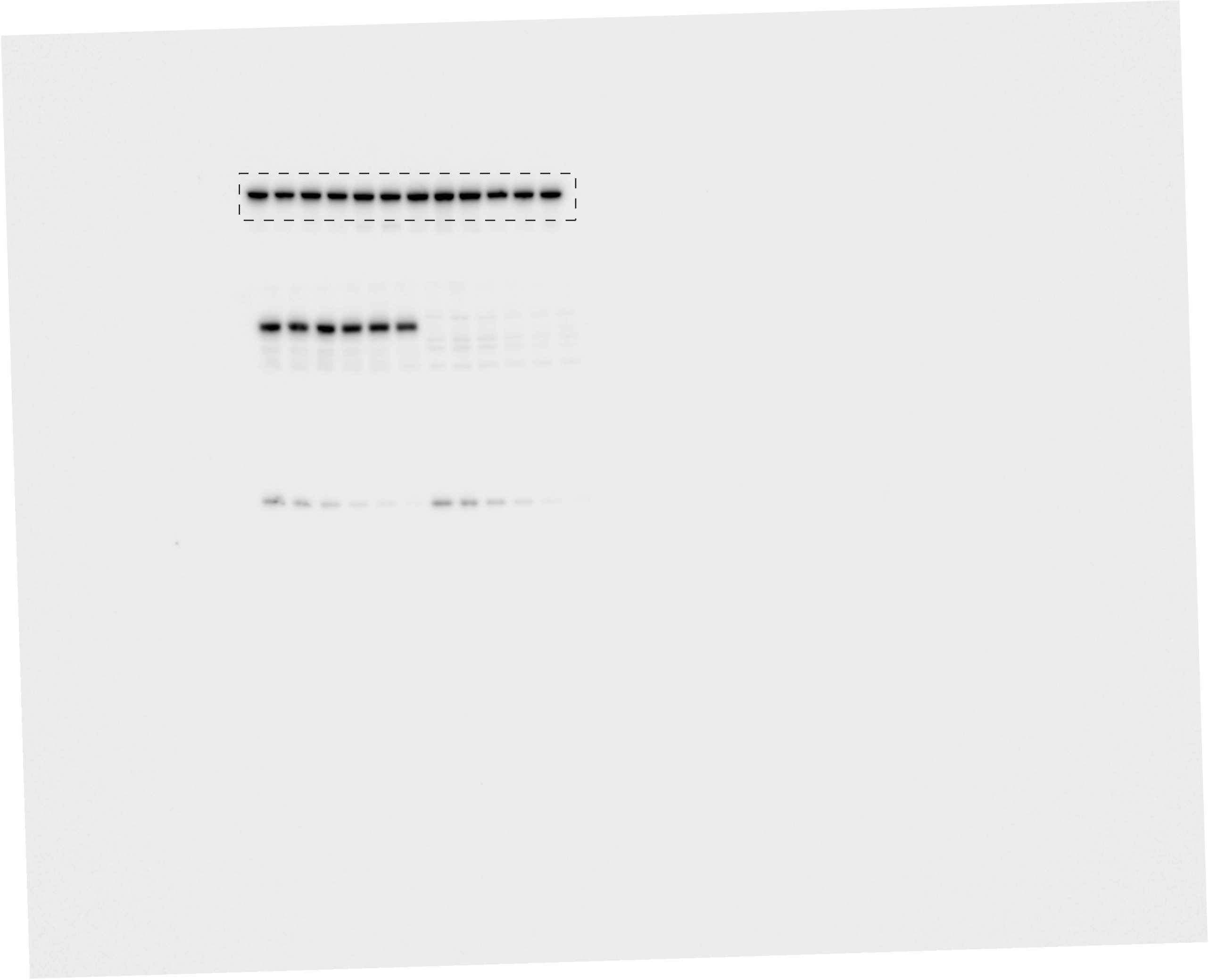

Figure 5b - PRIMPOL

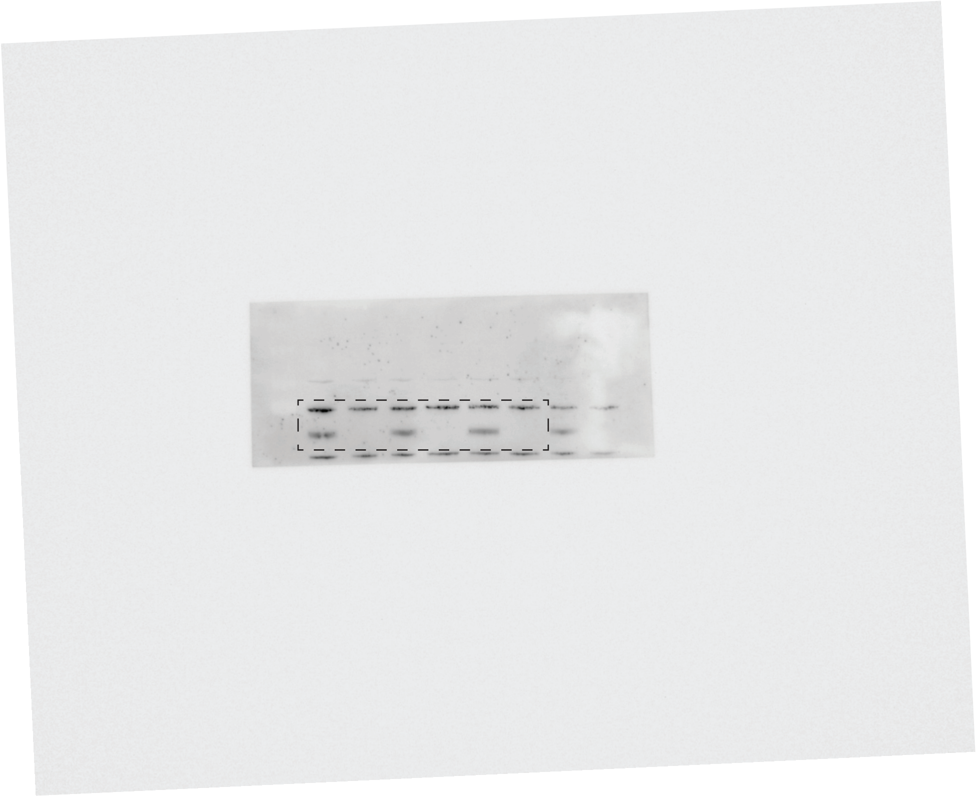

Figure 5b - USP1

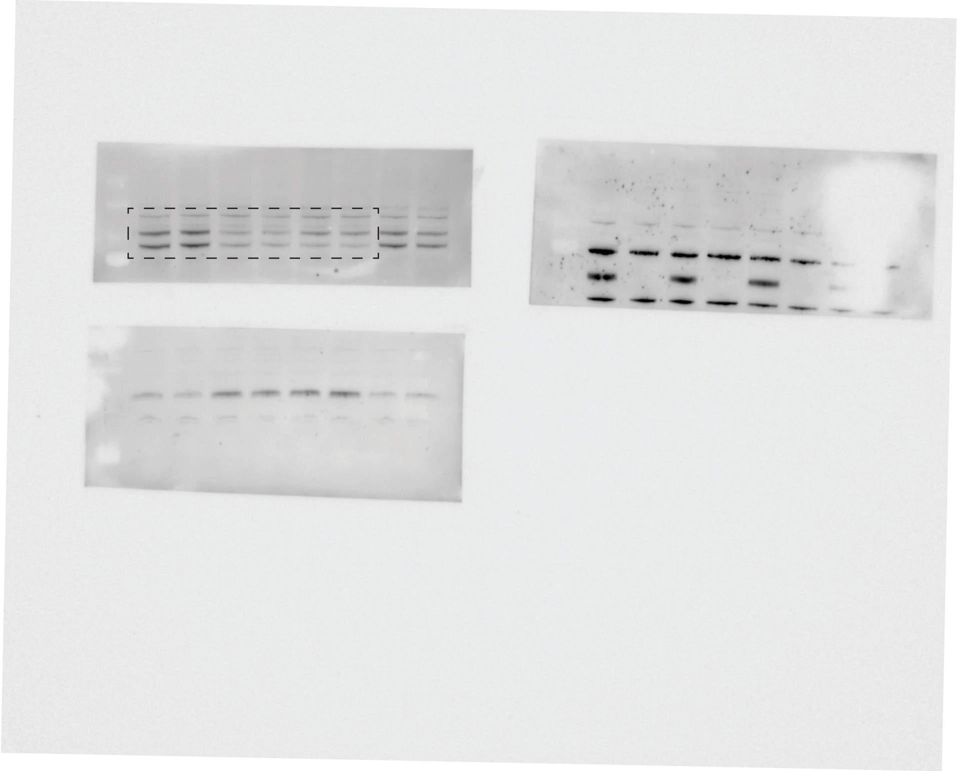

Figure 5b - WDR48

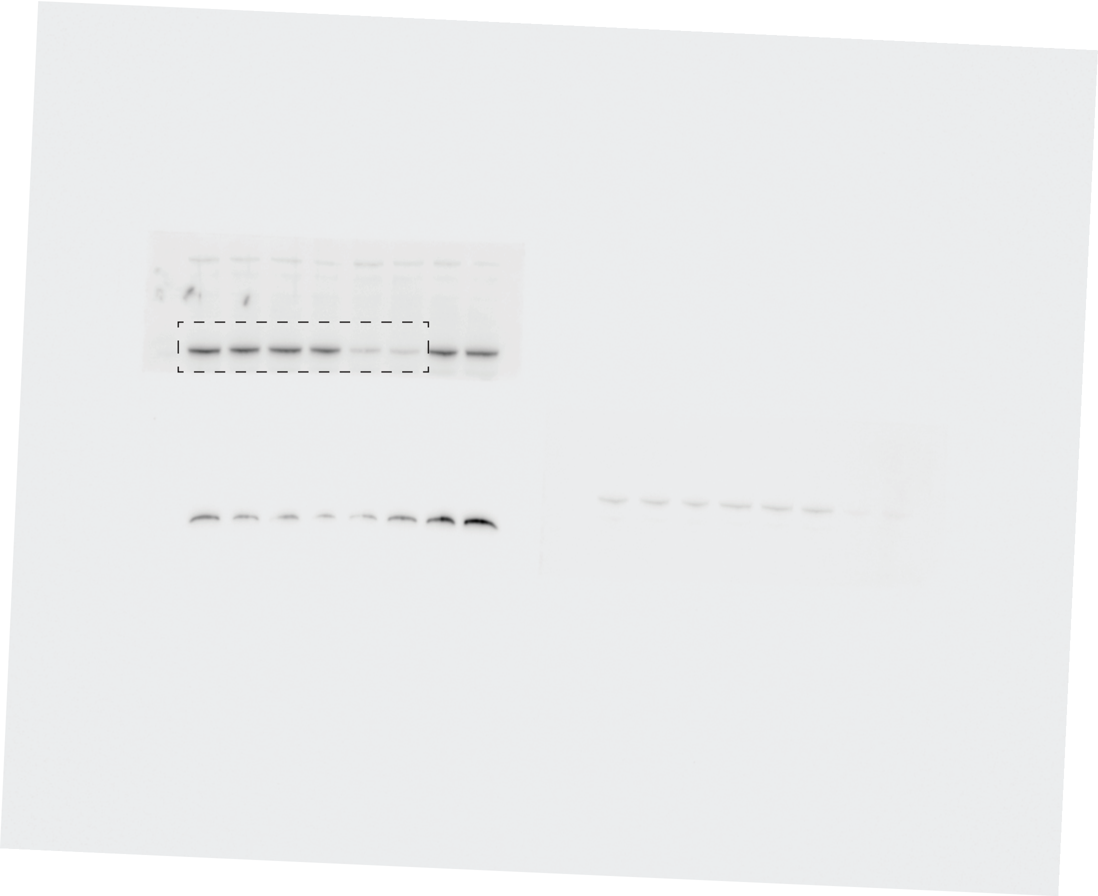

Figure 5b - ponceau

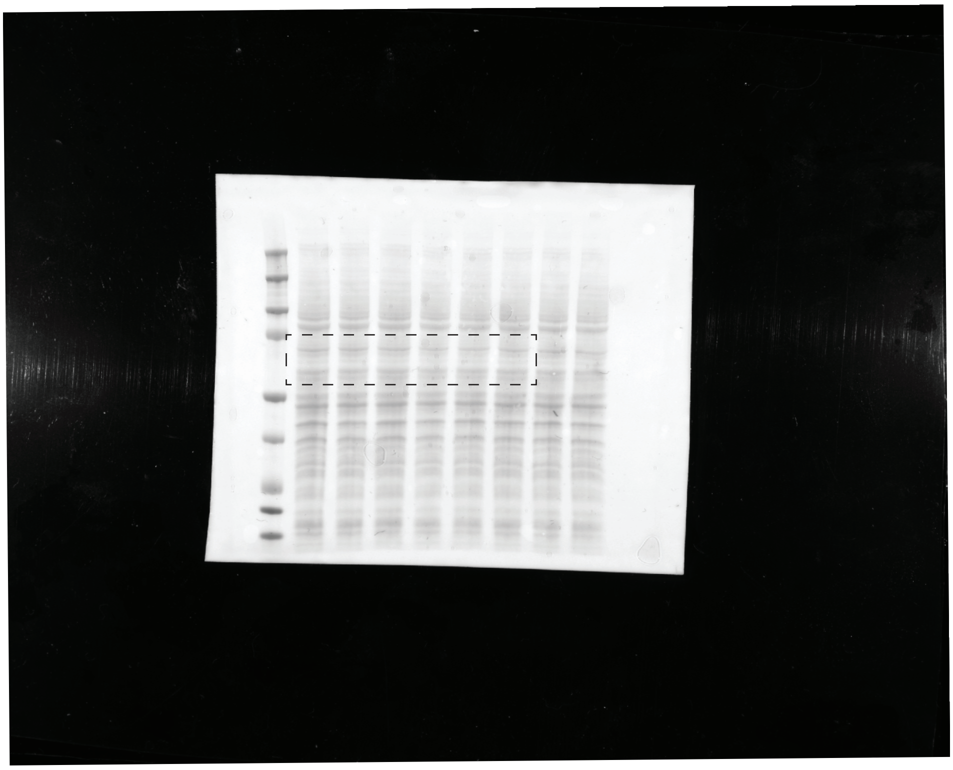

Figure 5f - USP1

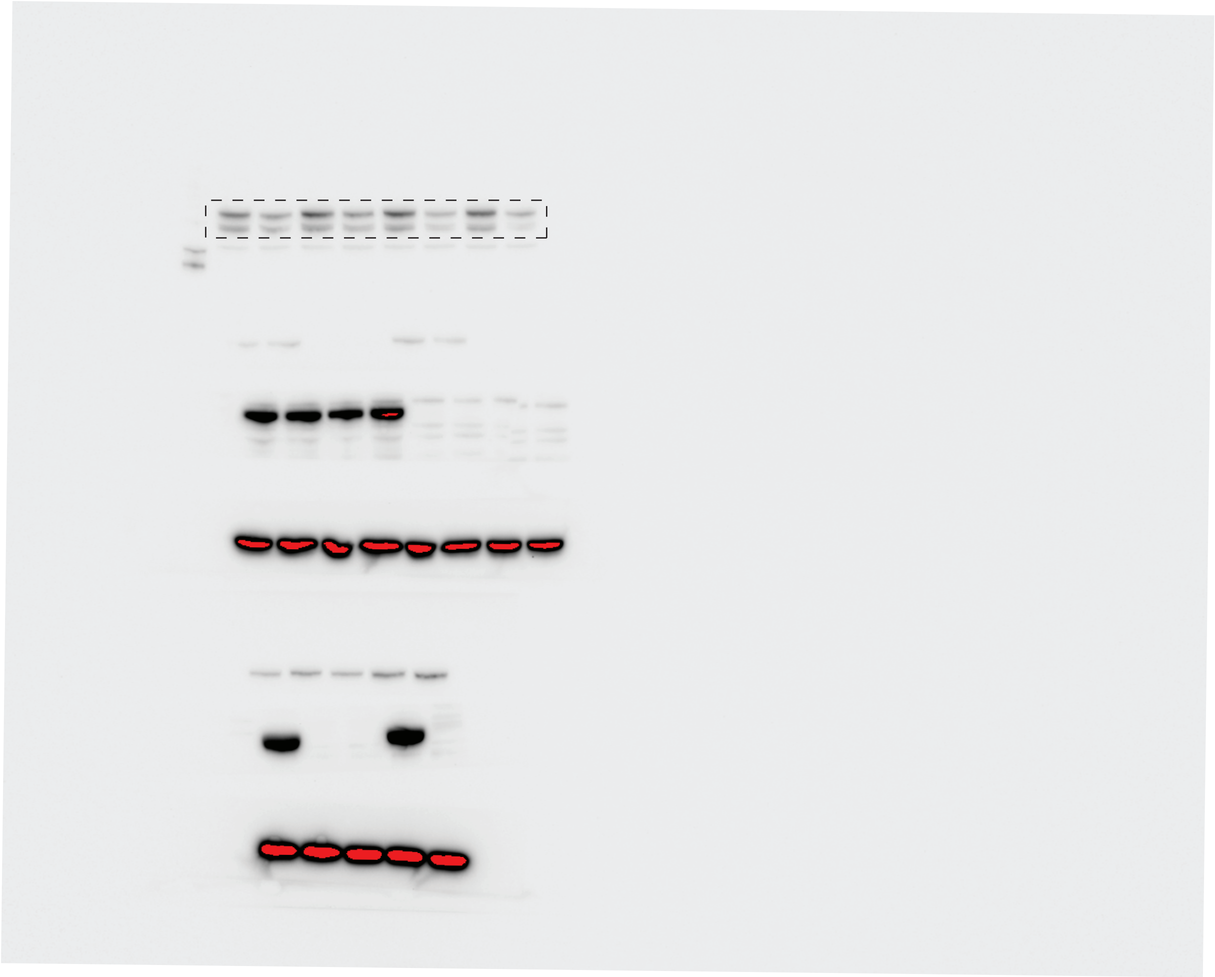

Figure 5f - SLFN11

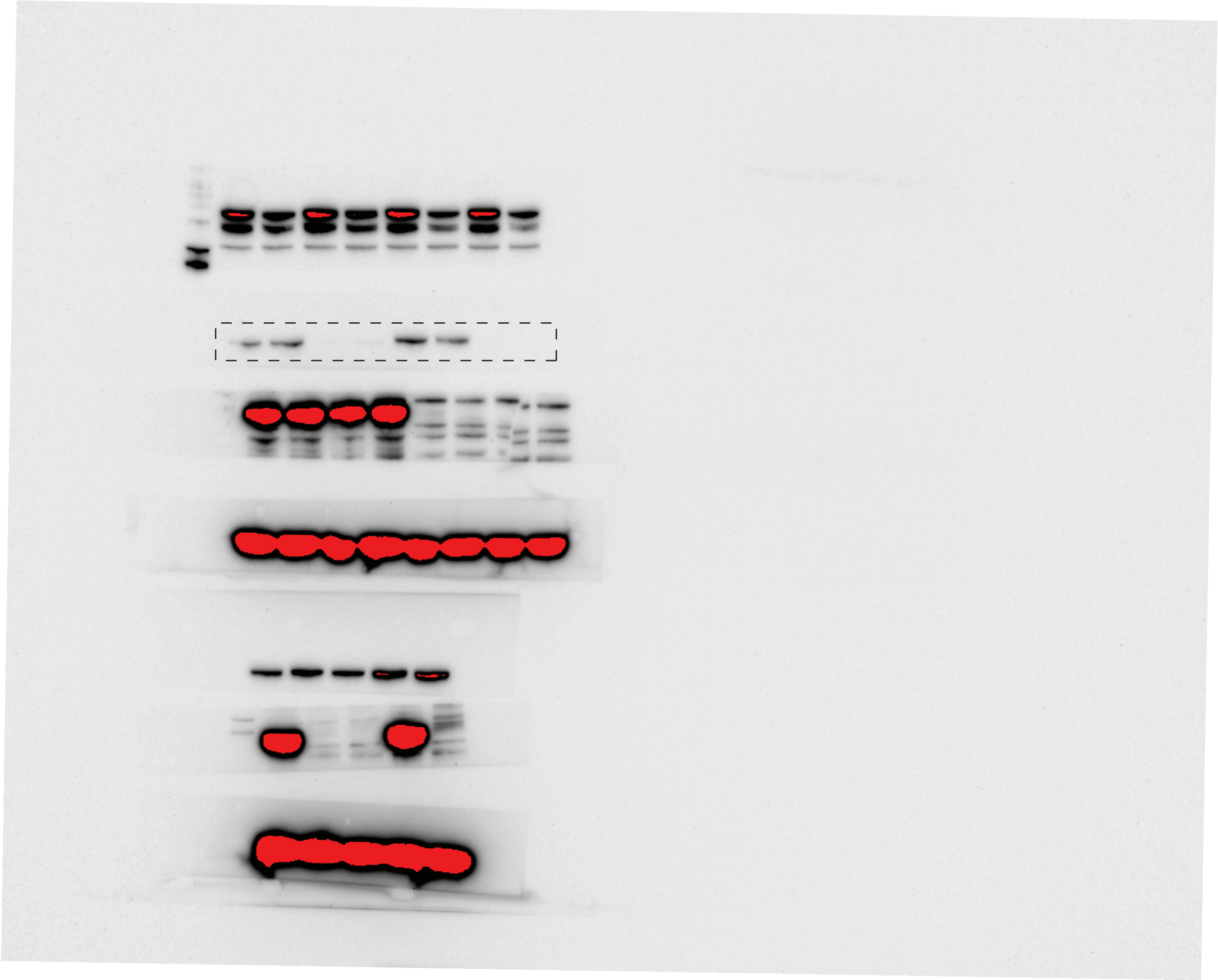

Figure 5f - PRIMPOL

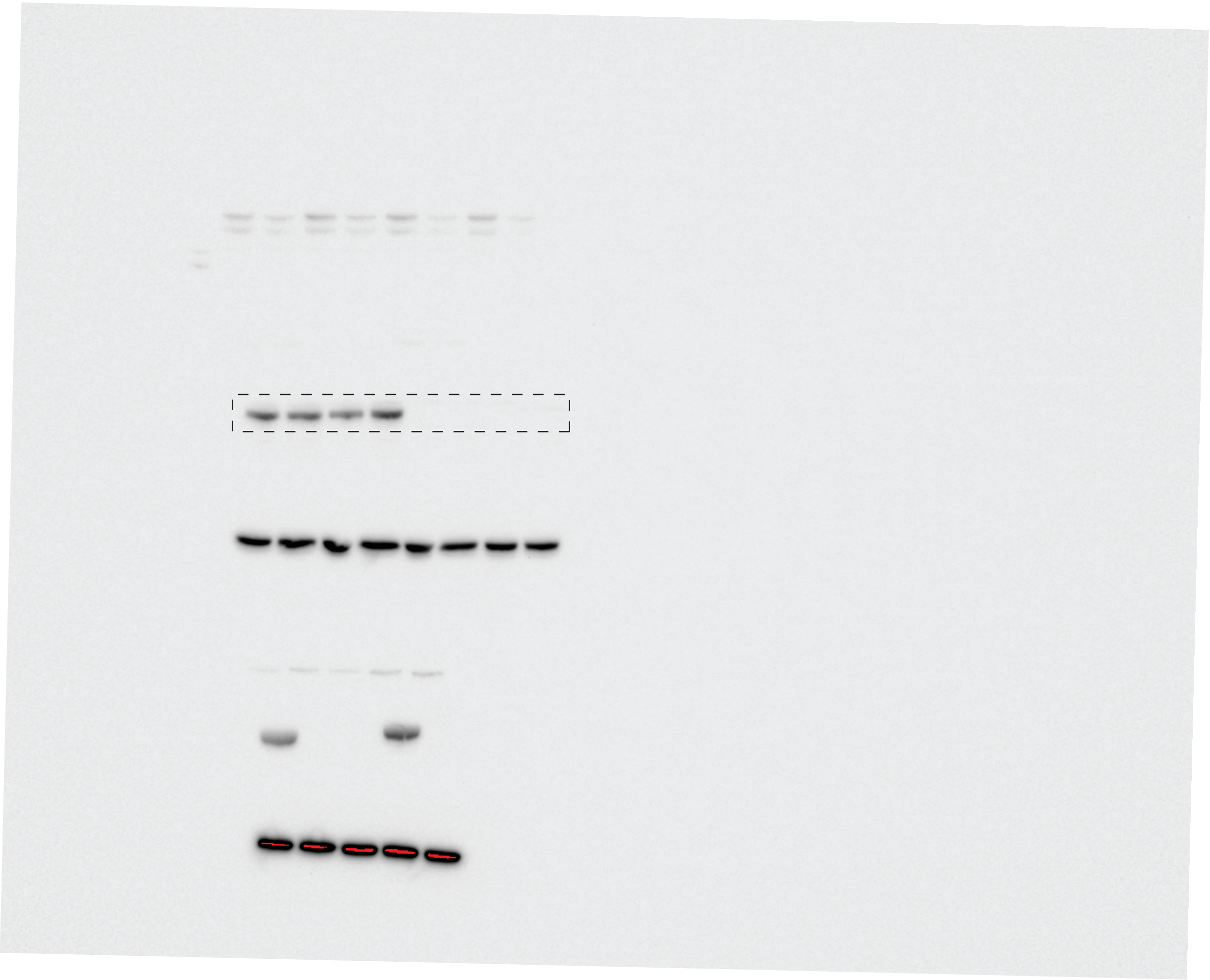

Figure 5f - ponceau

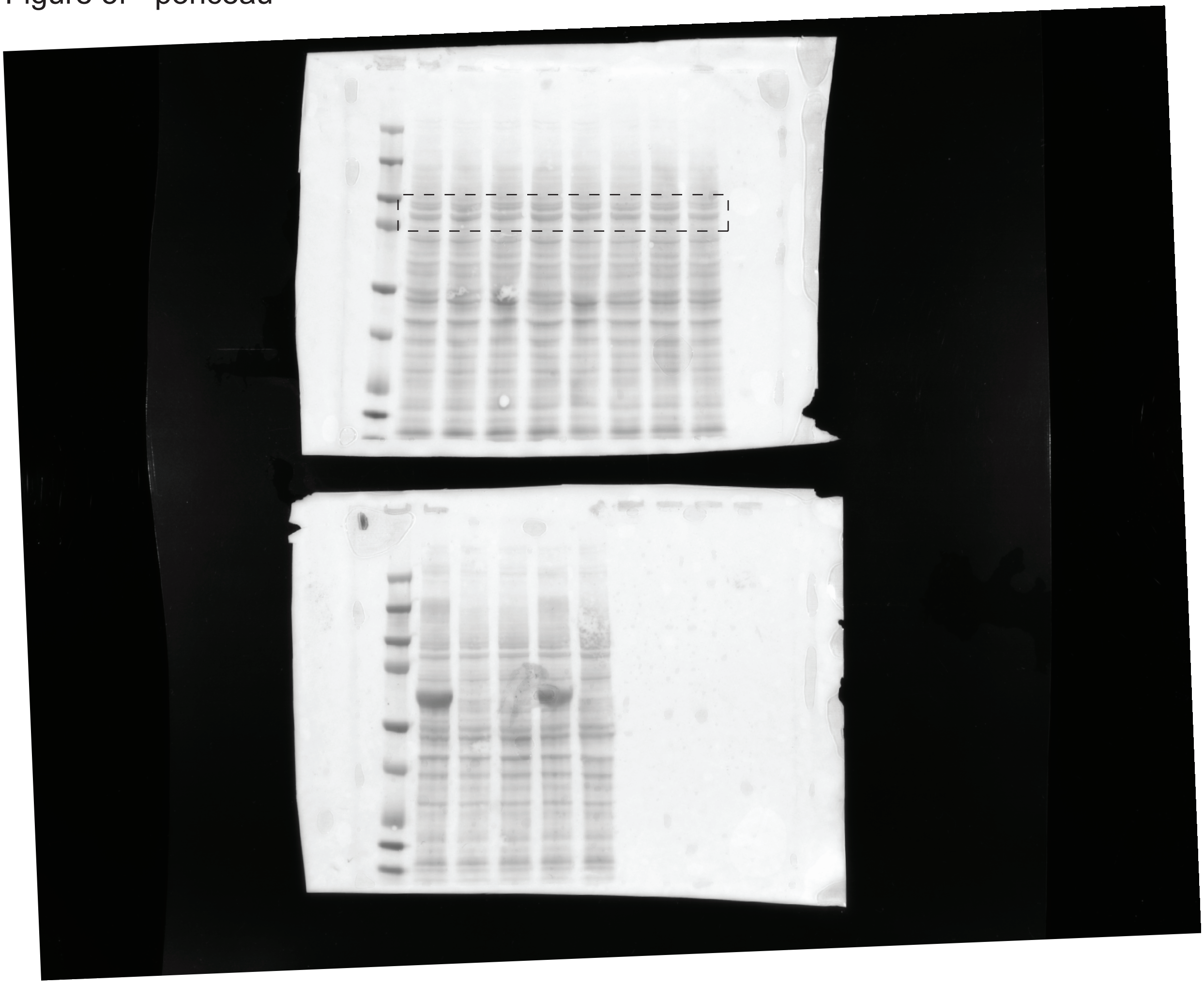

Figure 6d - SLFN11

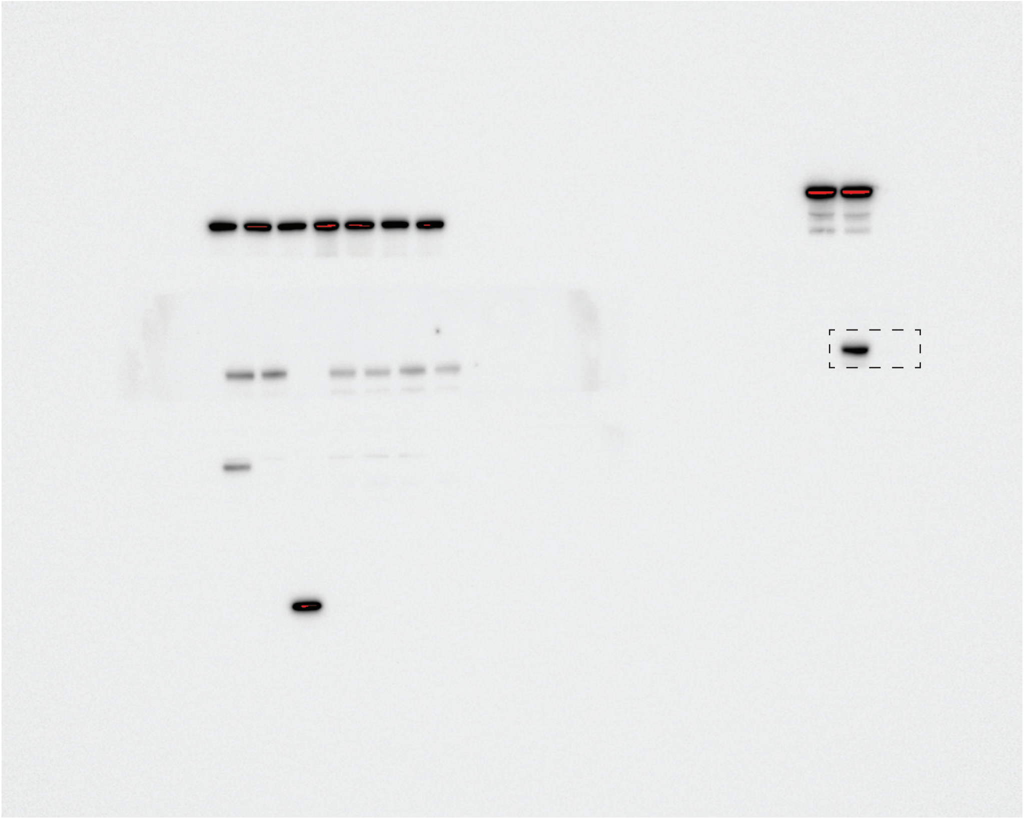

Figure 6d - vinculin

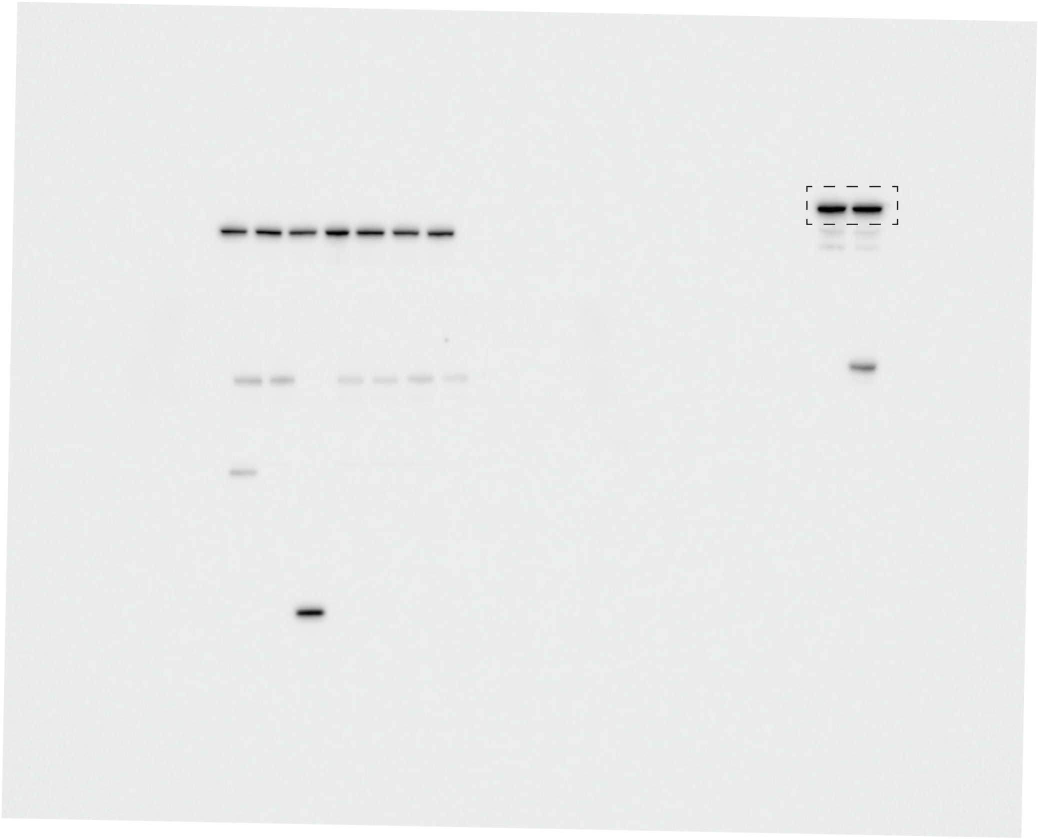

Figure 7b - SLFN11

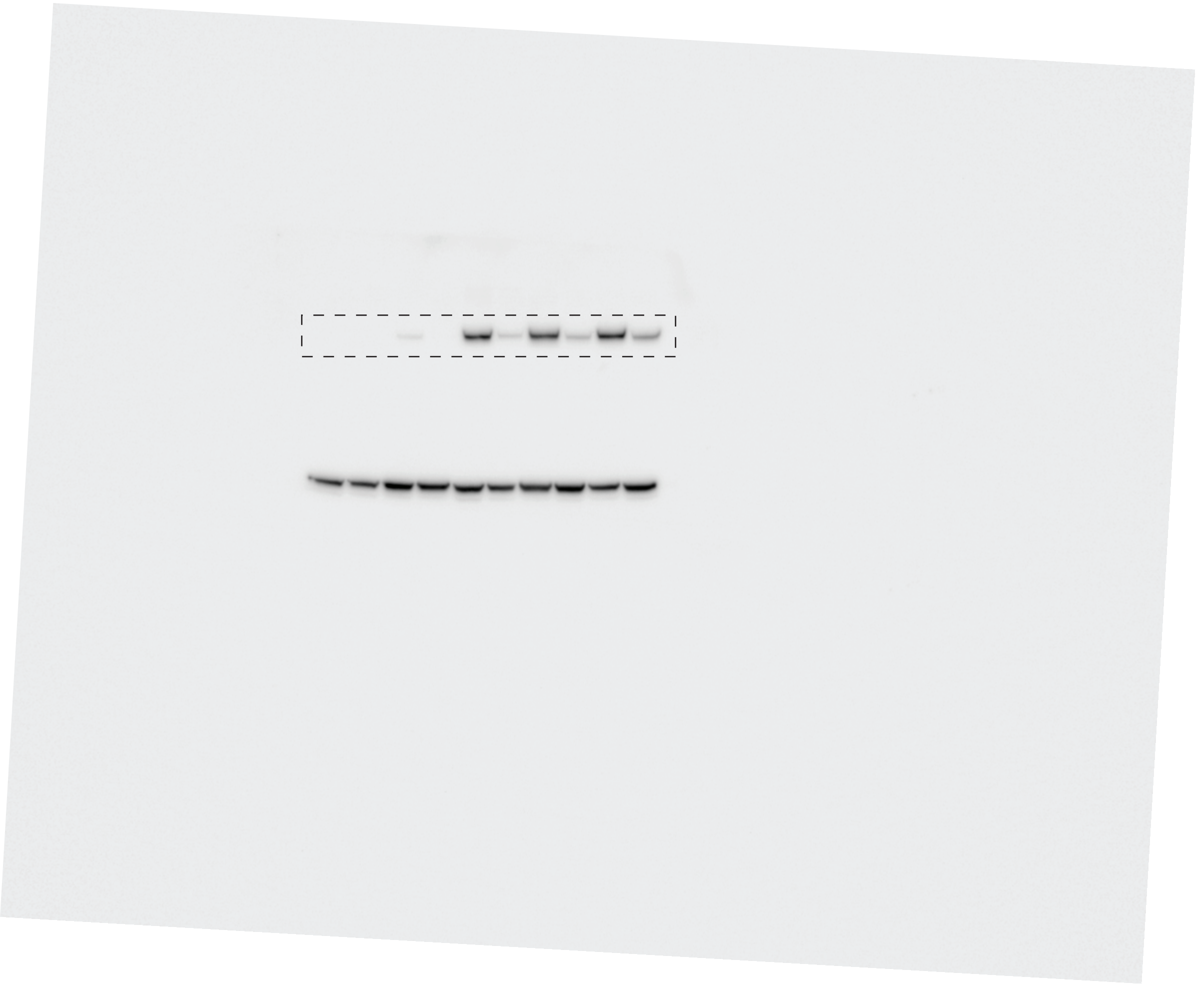

Figure 7b - ponceau

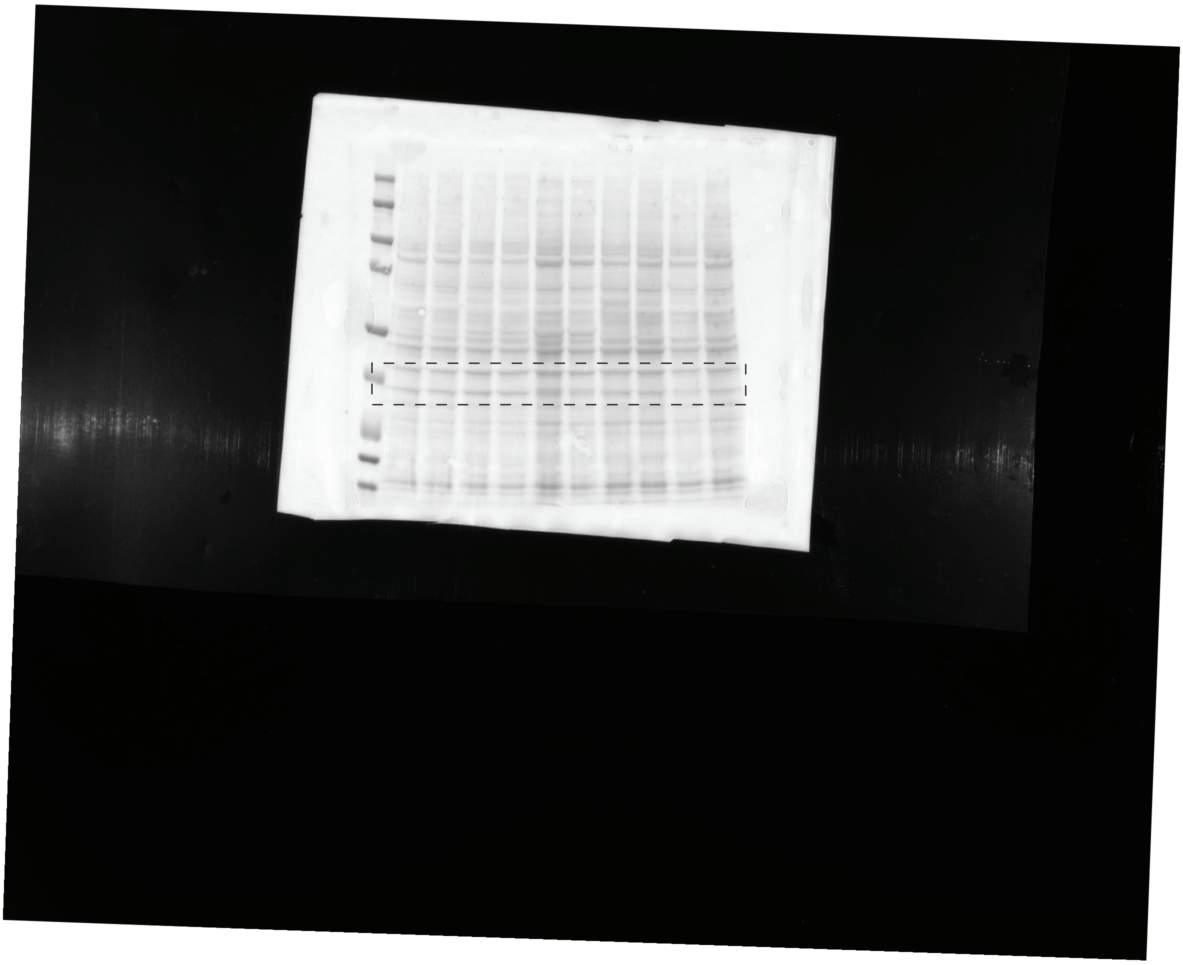

Extended Data 1g - PRIMPOL

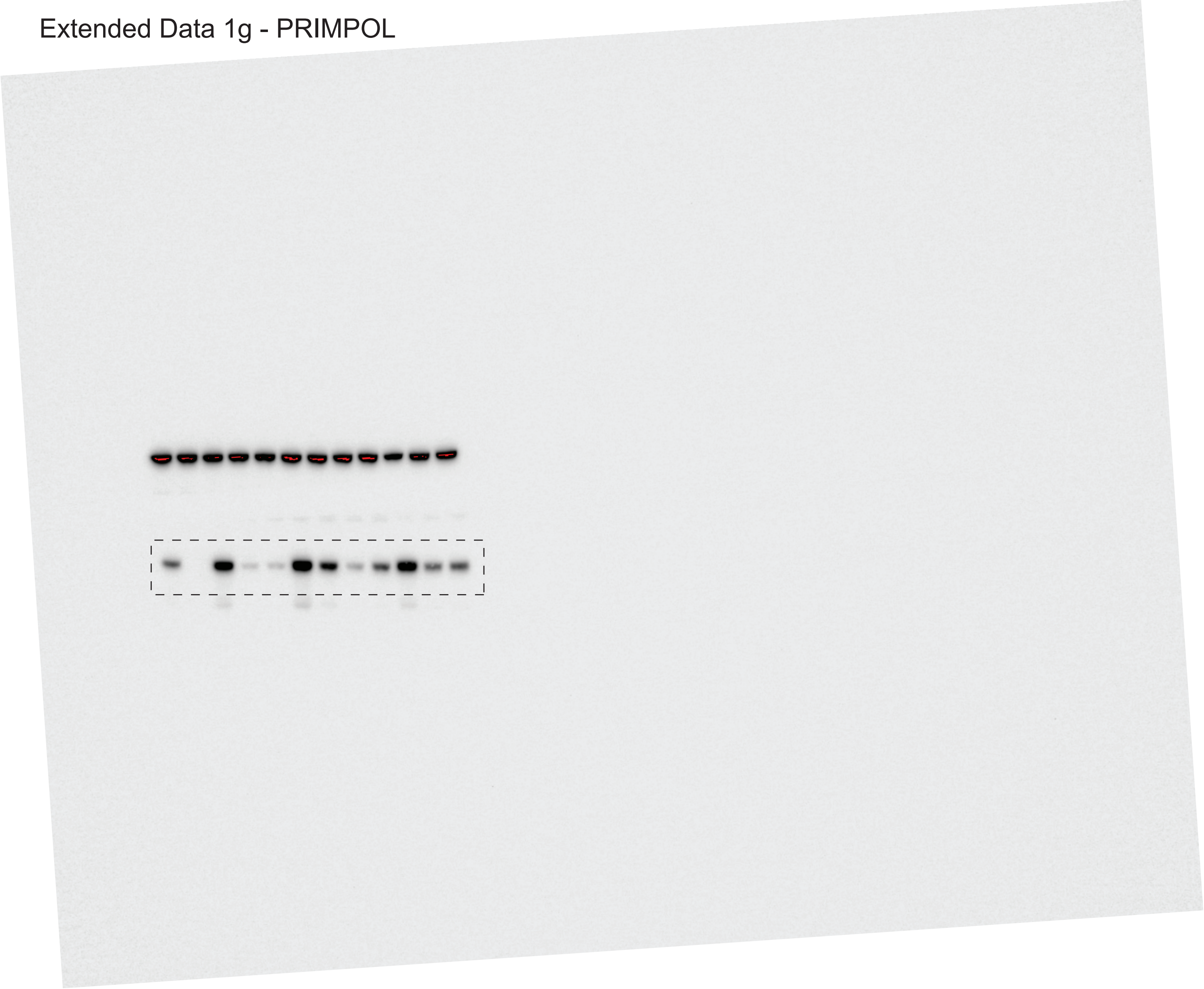

Extended Data 1g - vinculin

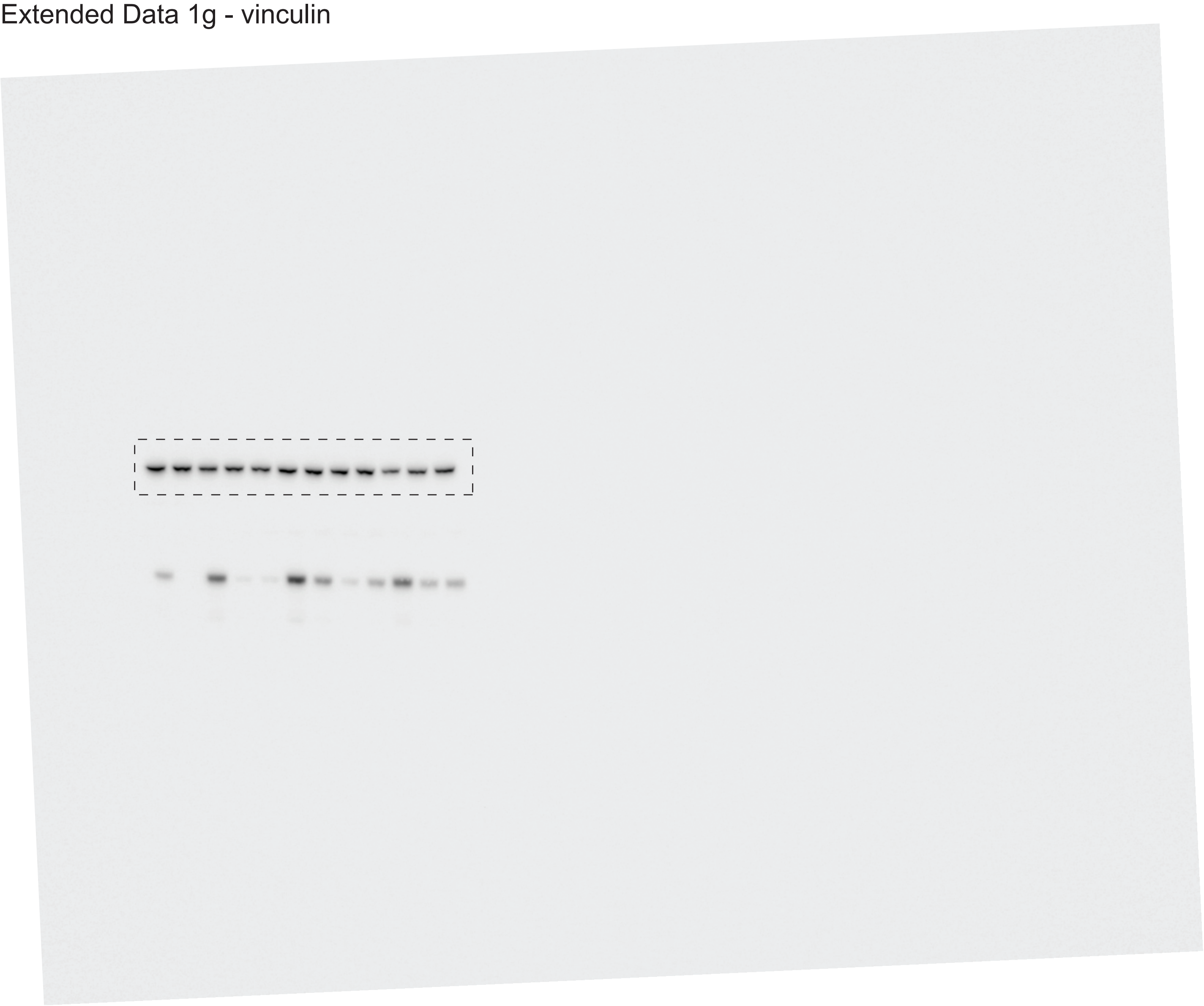

Extended Data 2b - SLFN11

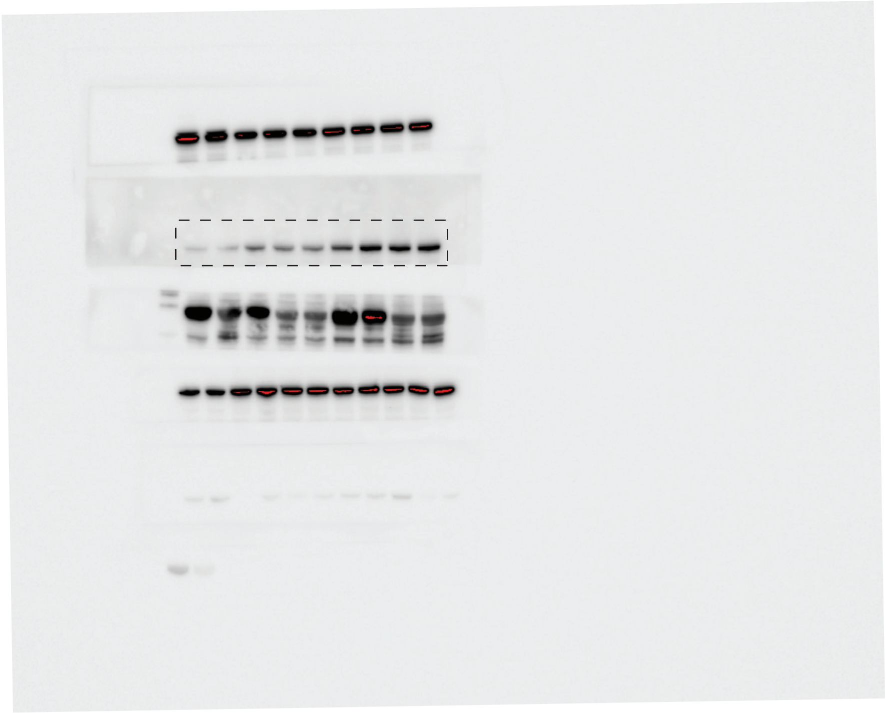

Extended Data 2b - PRIMPOL

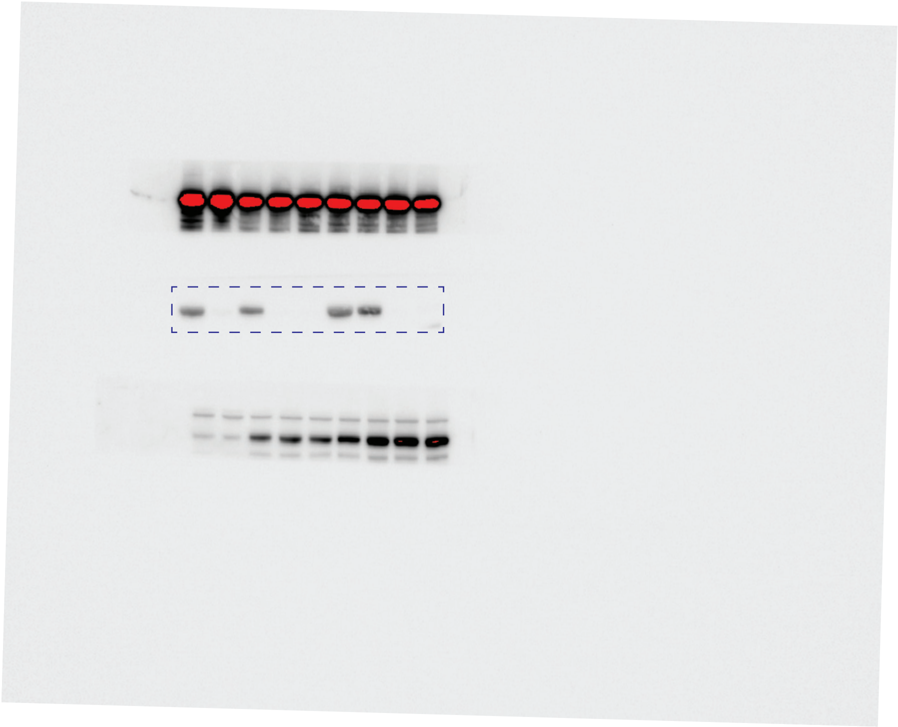

Extended Data 2b - vinculin

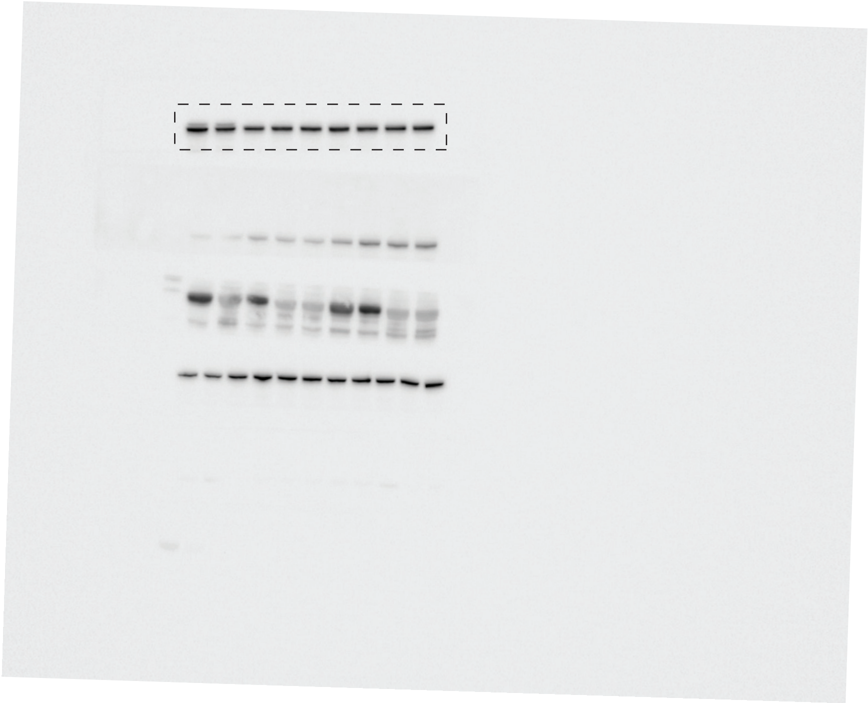

Extended Data 2b - ponceau

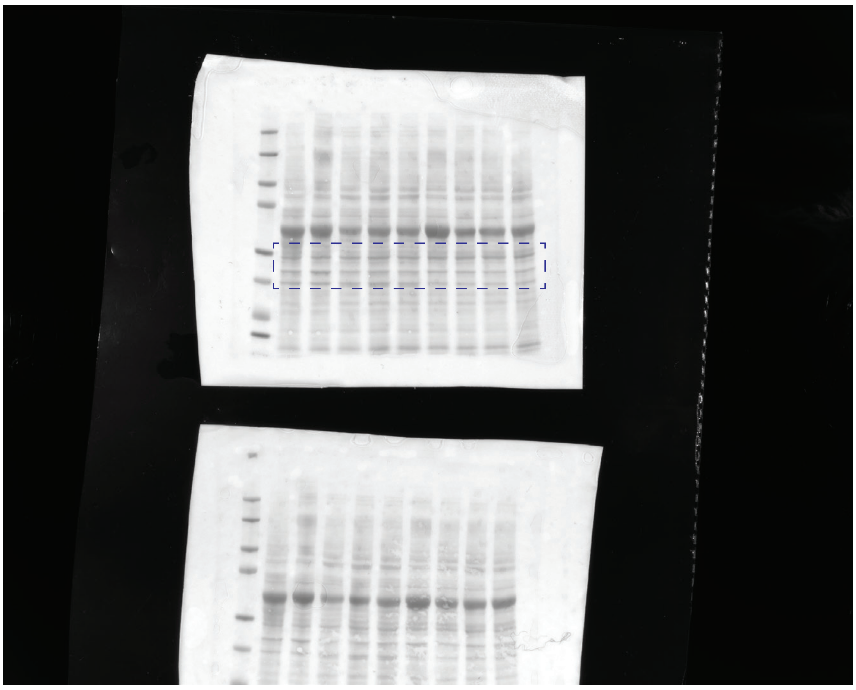

Extended Data 4e - PRIMPOL

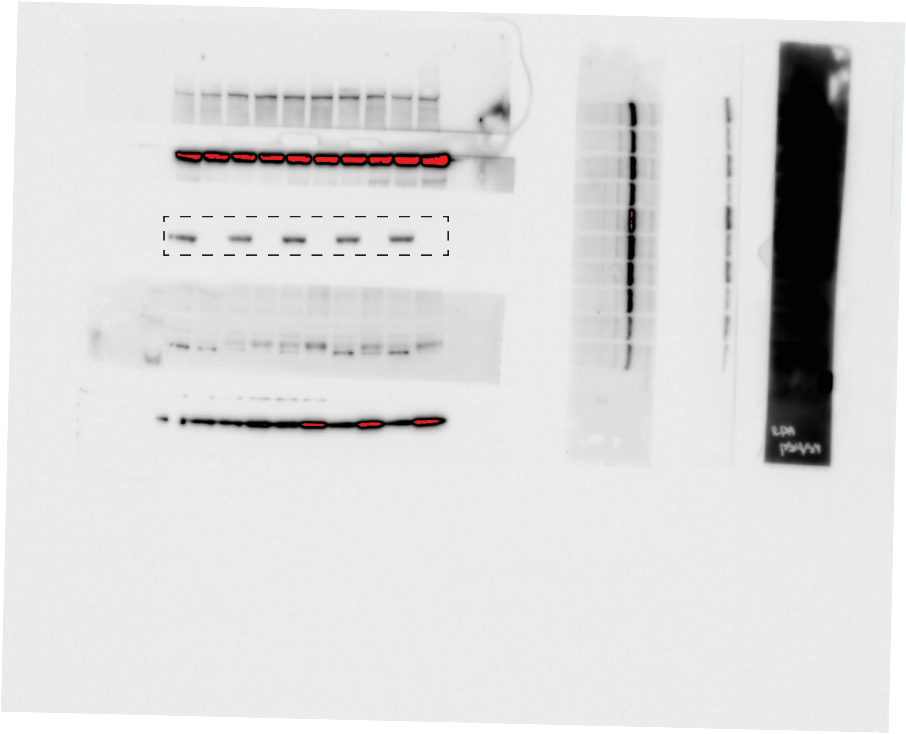

Extended Data 4e - RPA pS33

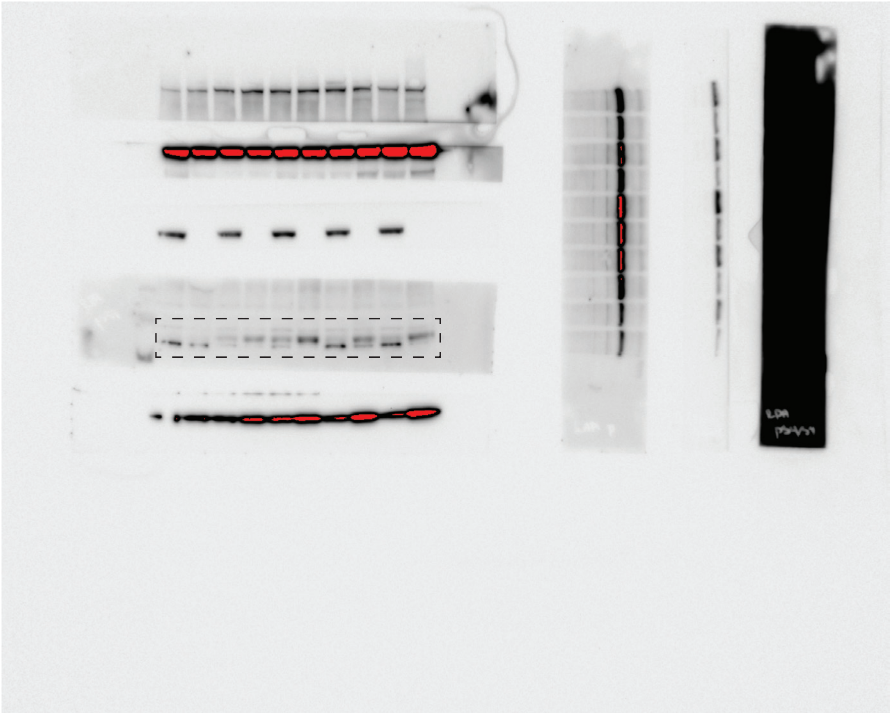

Extended Data 4e - RPA2

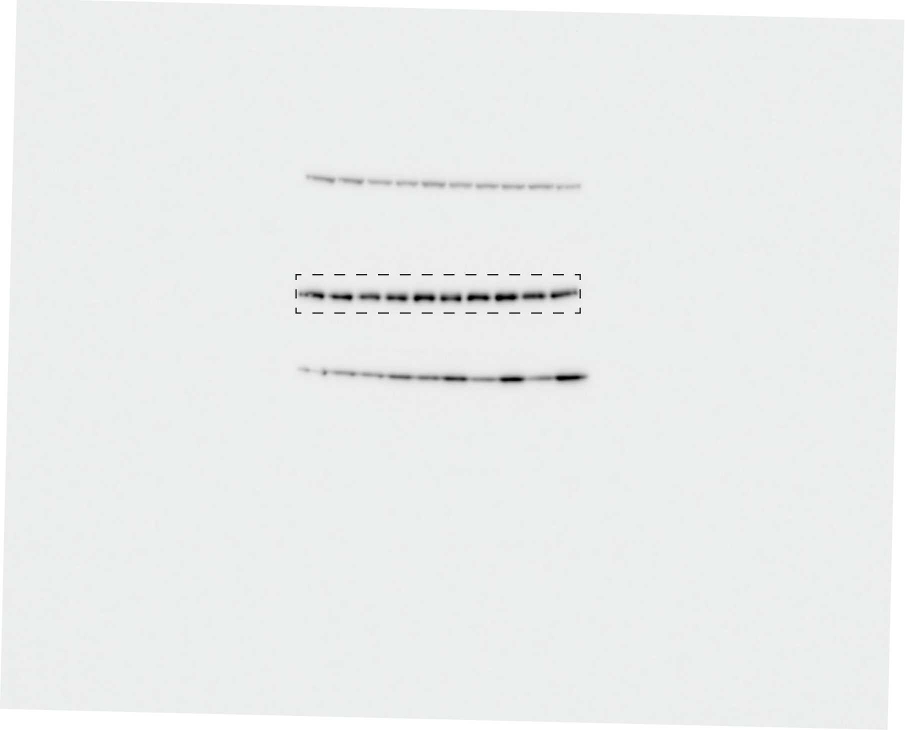

Extended Data 4e - Chk1 pS345

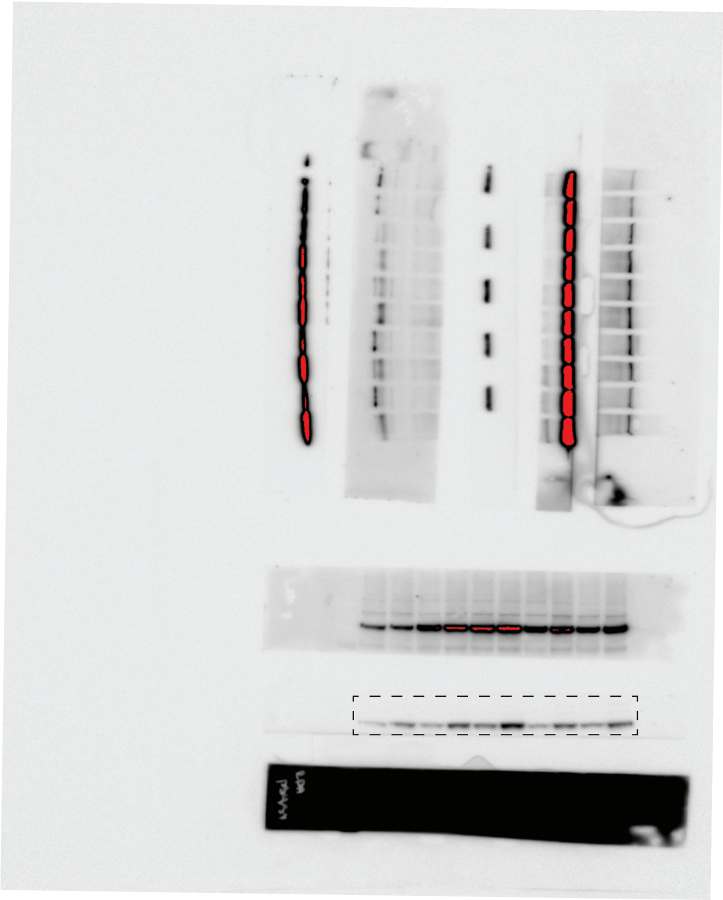

Extended Data 4e - Chk1

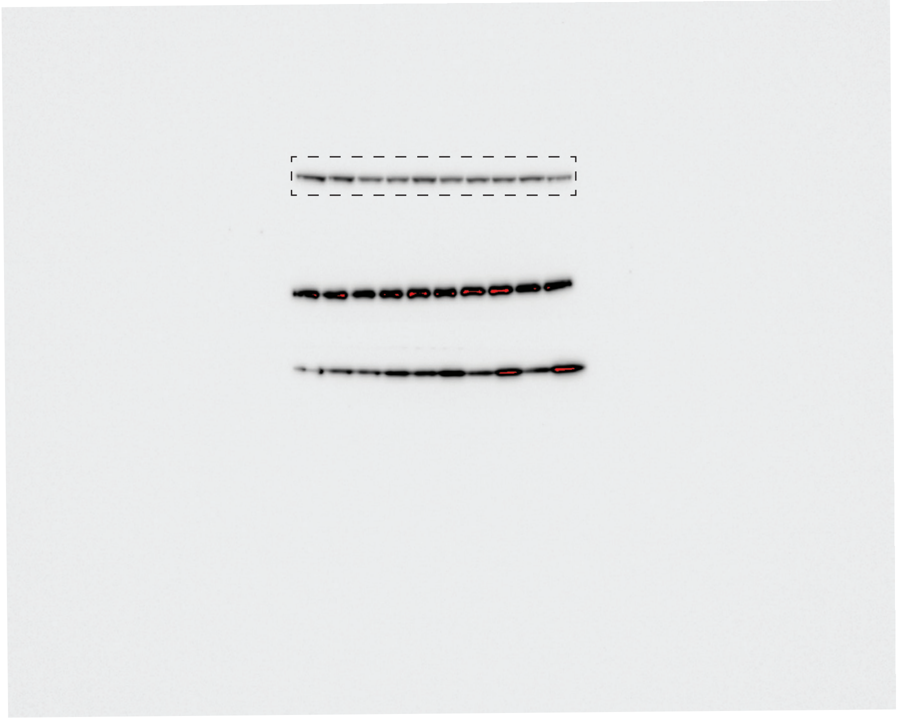

Extended Data 4e - vinculin

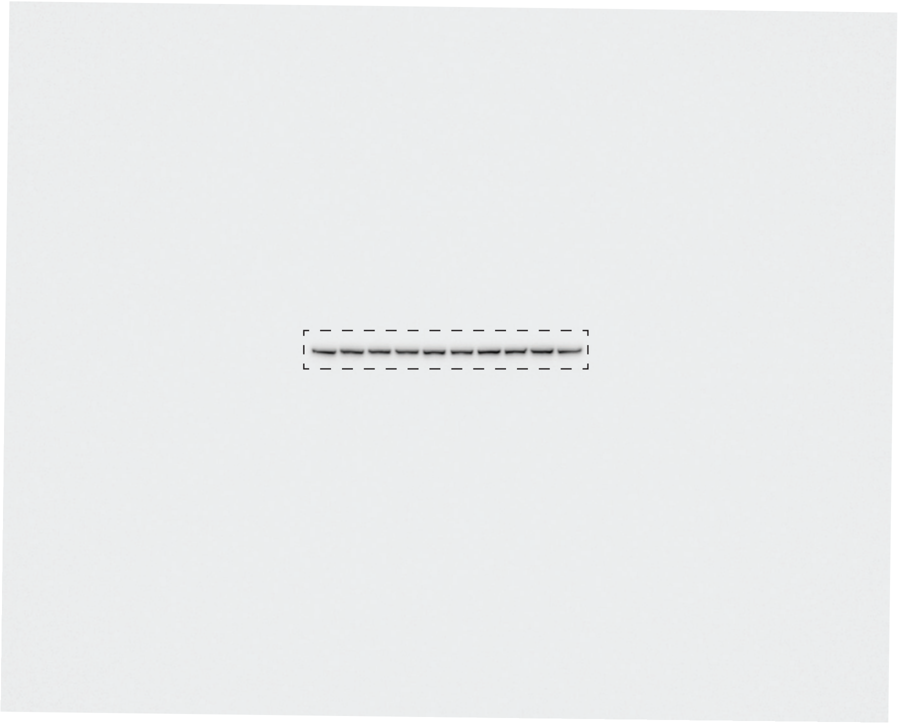

Extended Data 7b - PRIMPOL

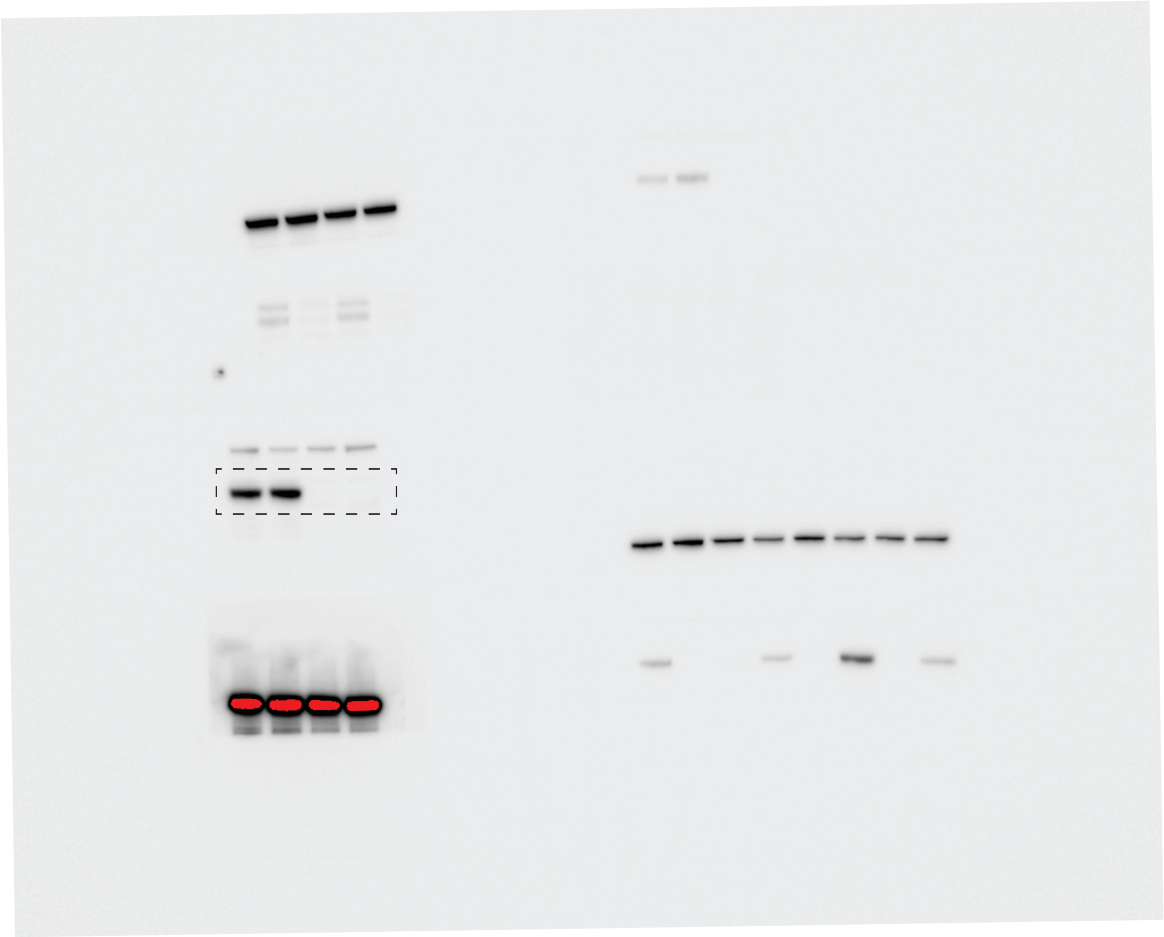

Extended Data 7b - RAD18

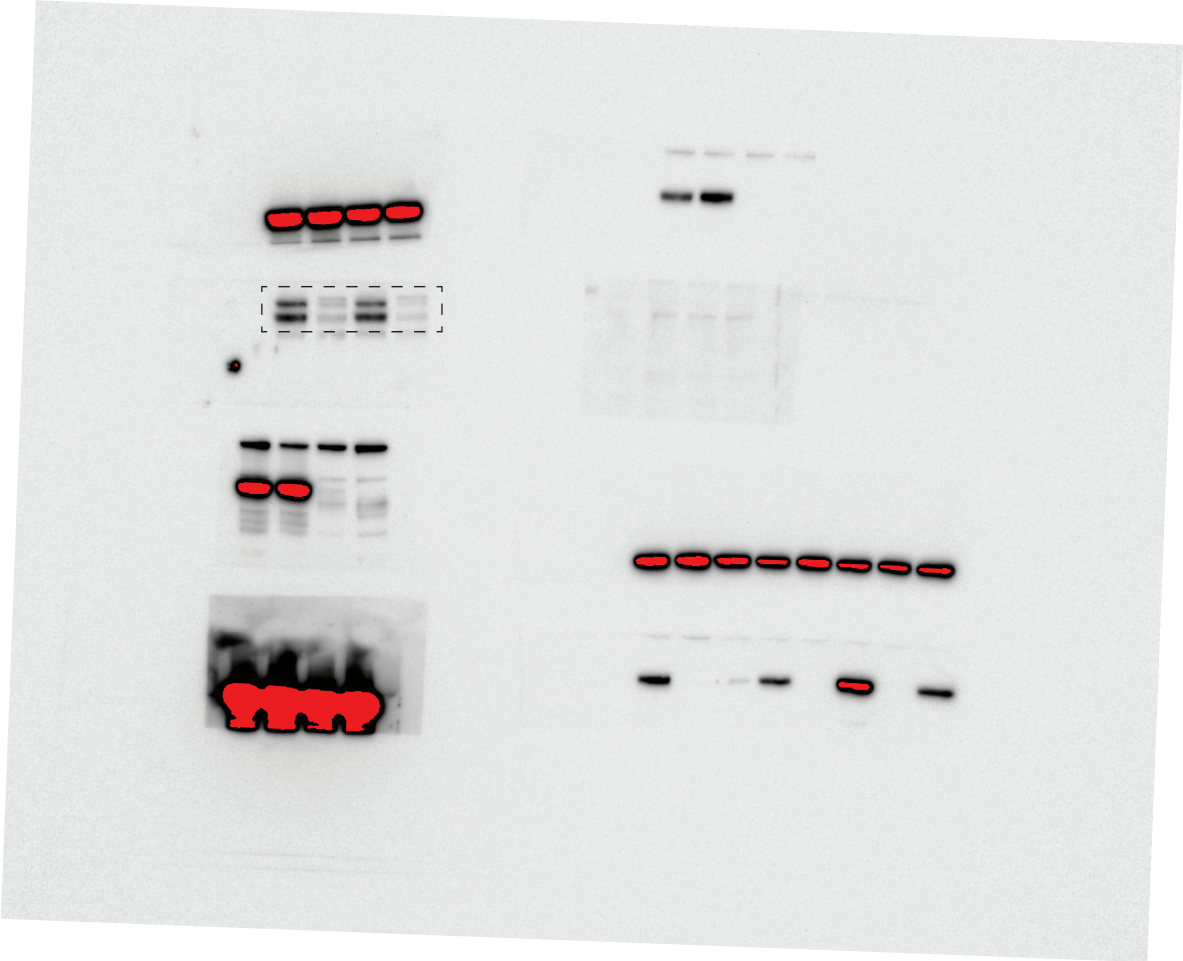

Extended Data 7b - vinculin

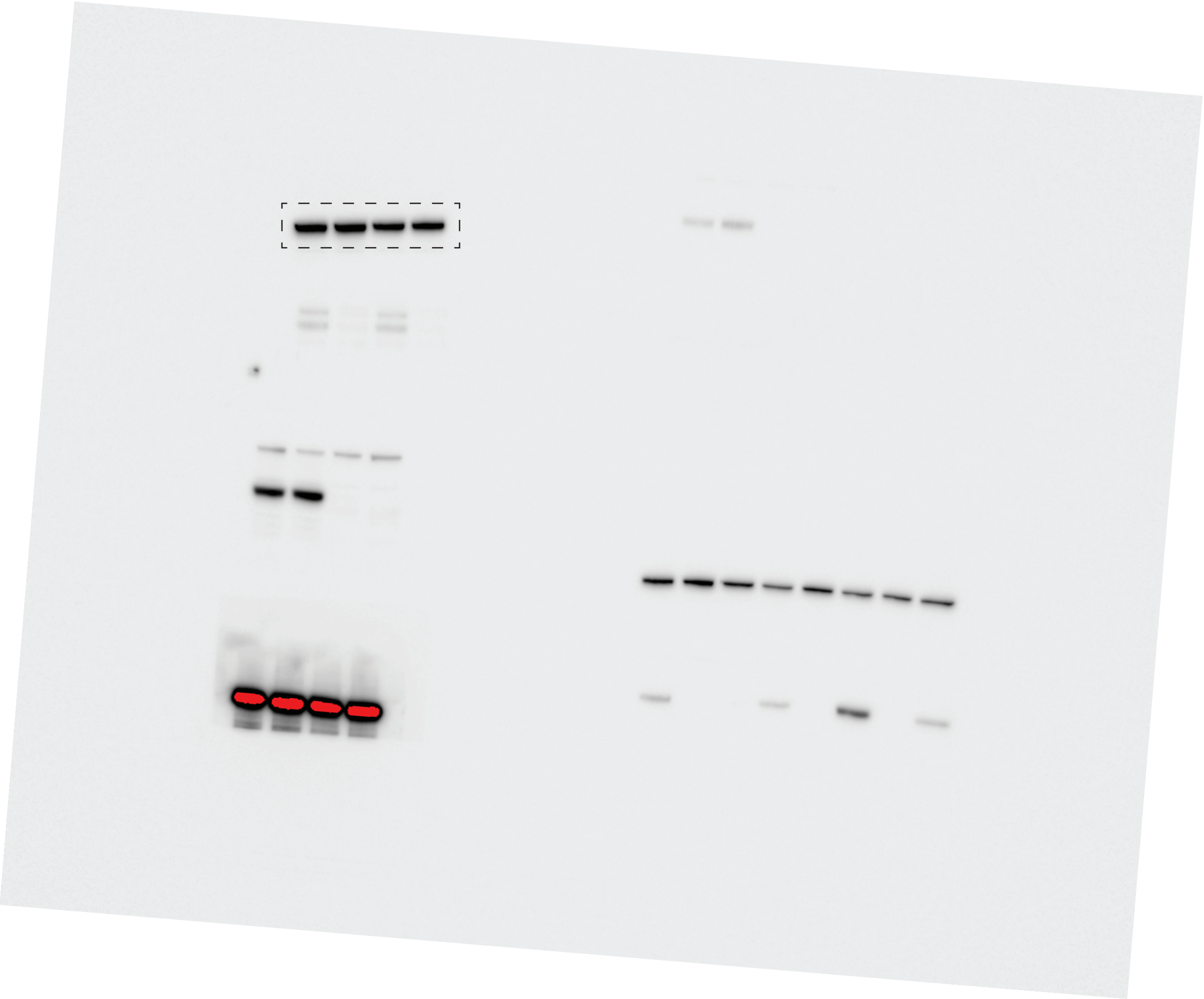

Extended Data 7c - PRIMPOL

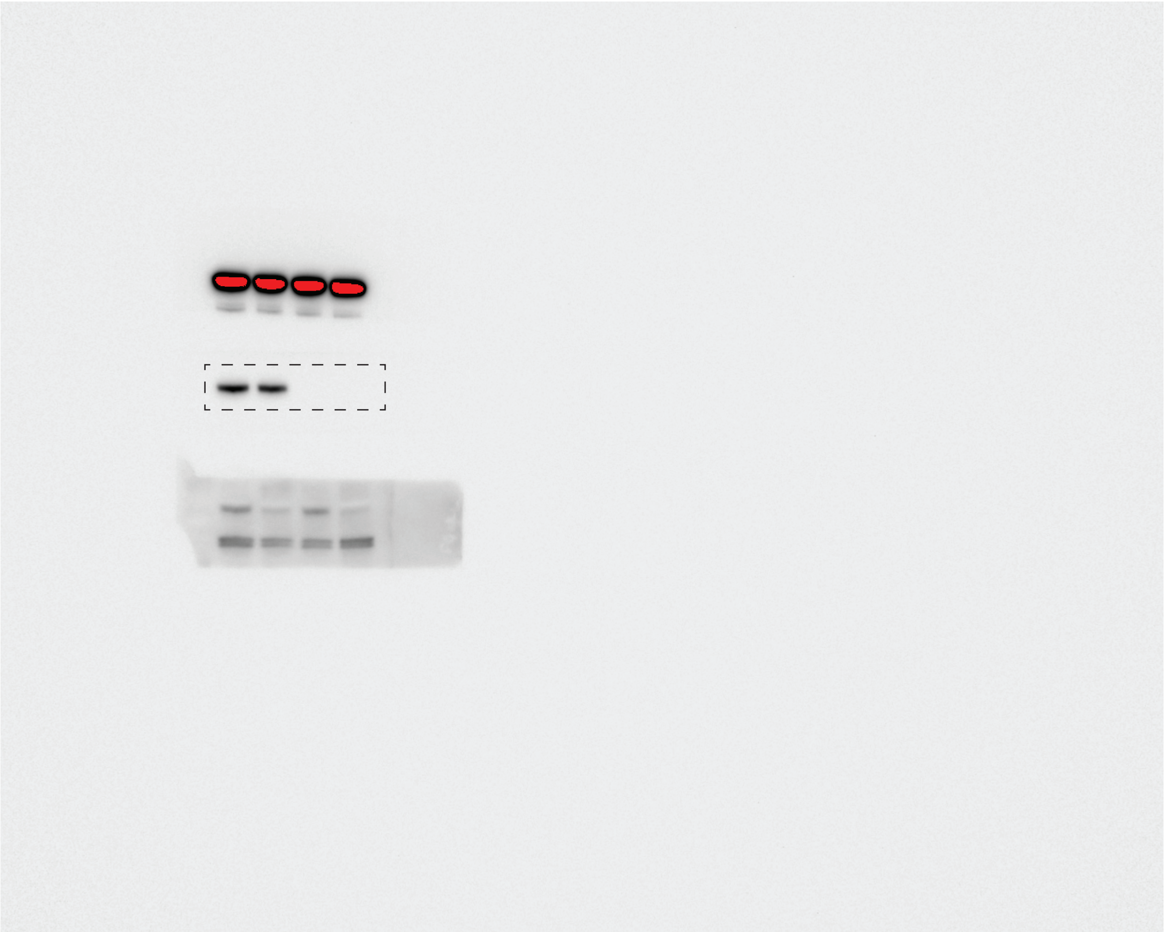

Extended Data 7c - FANCL

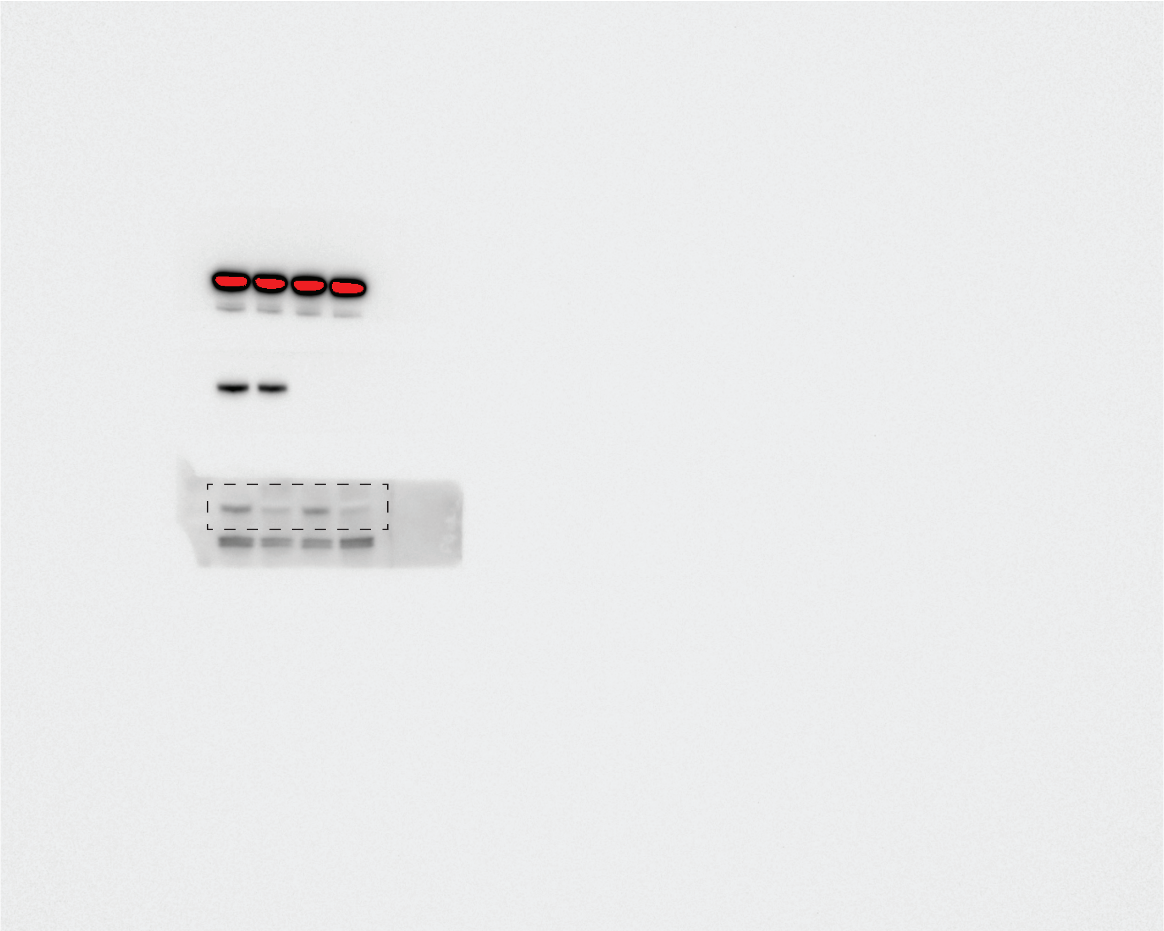

Extended Data 7c - vinculin

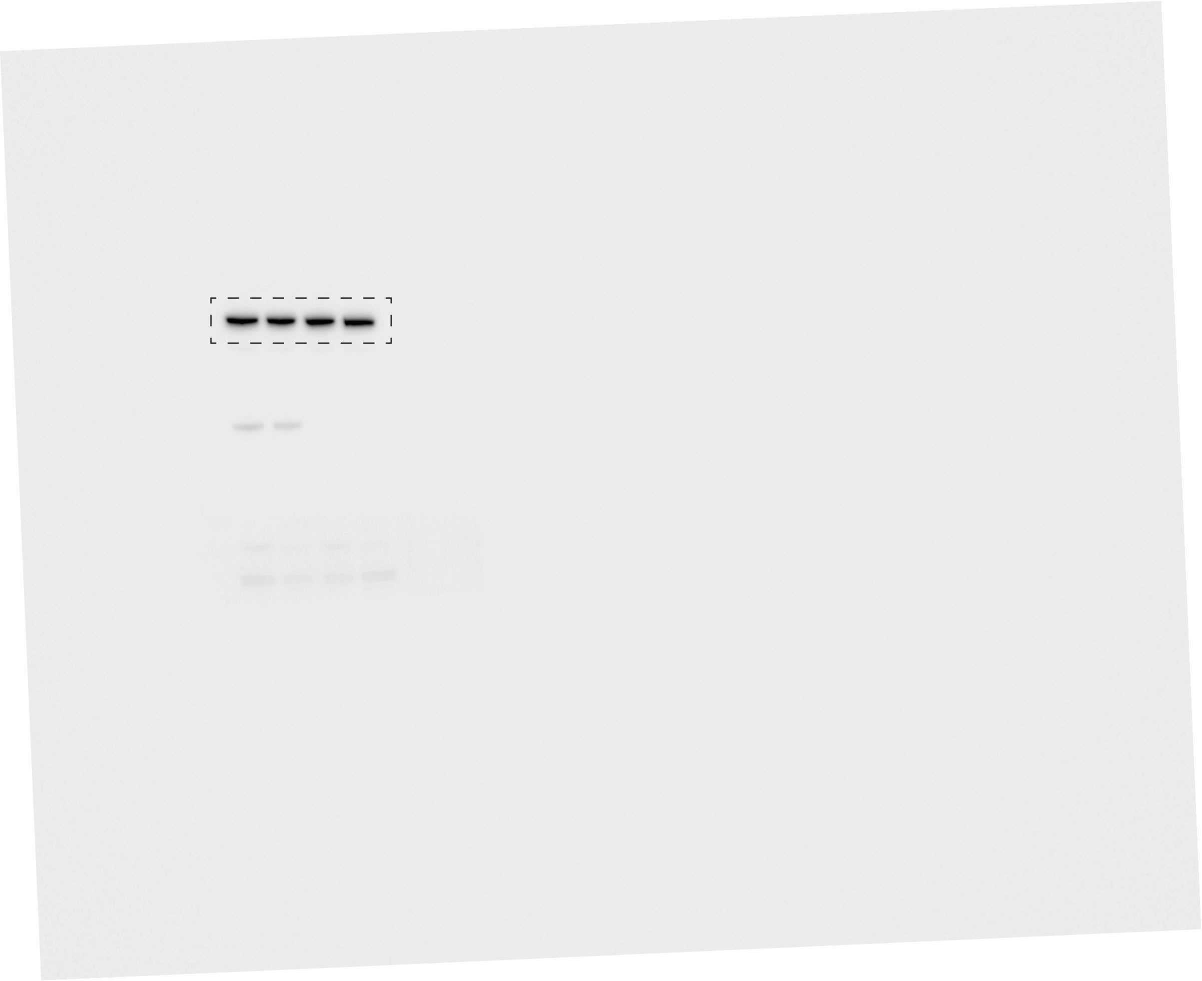

Extended Data 7f - PRIMPOL

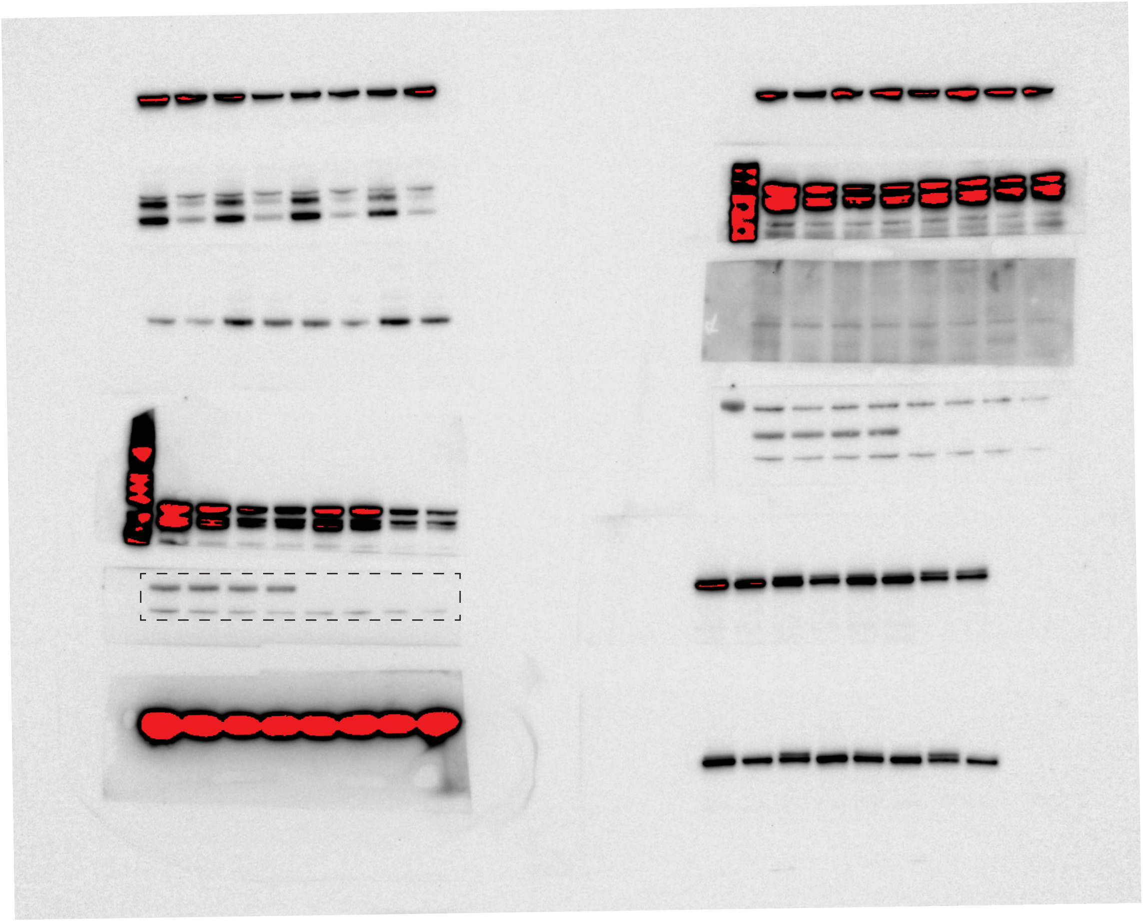

Extended Data 7f - RAD18

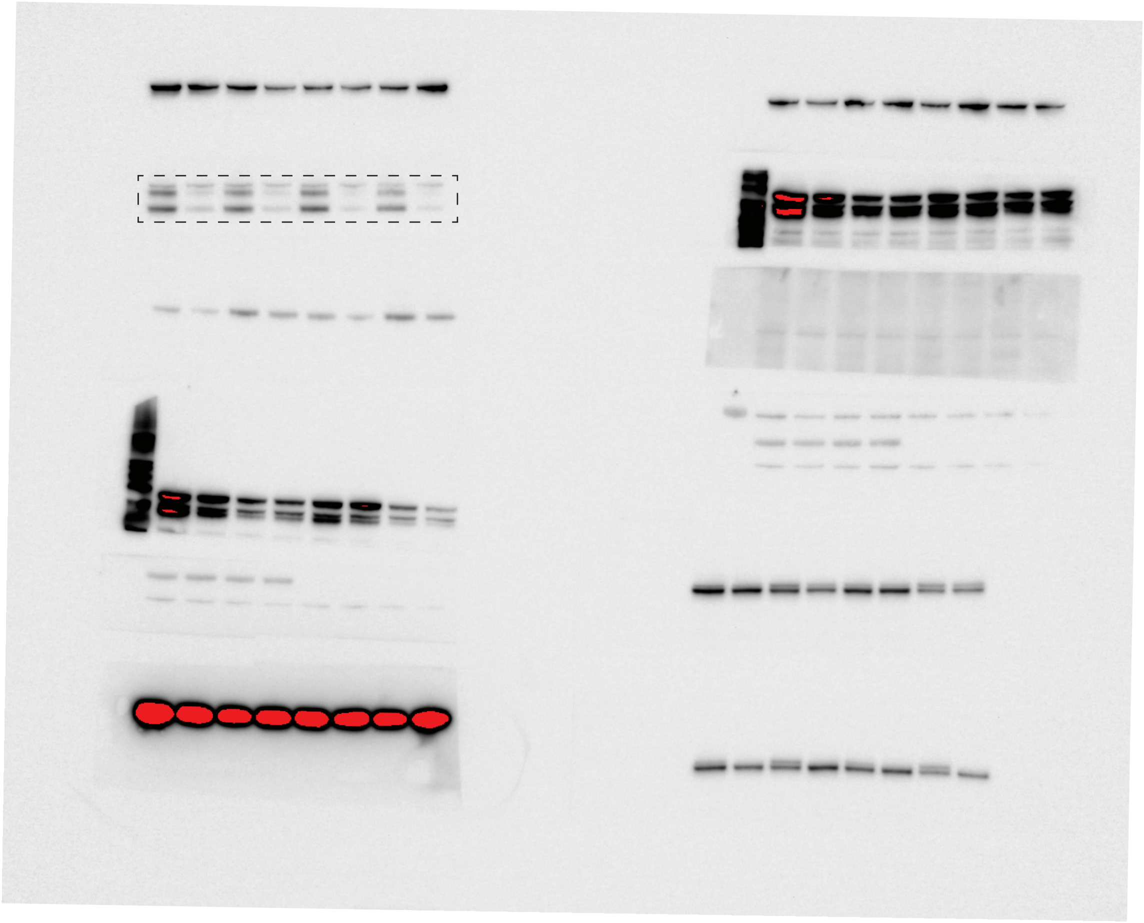

Extended Data 7f - USP1

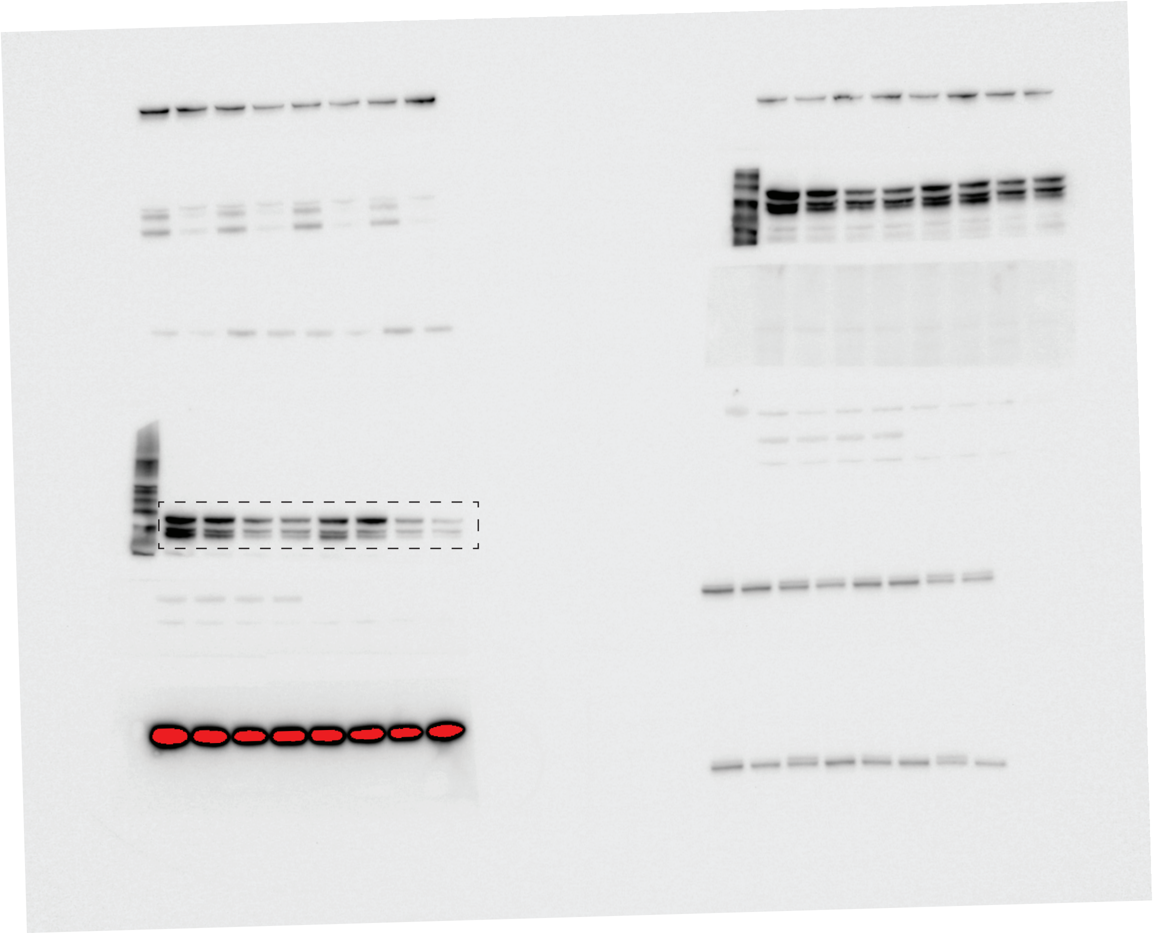

Extended Data 7f - PCNA-Ub

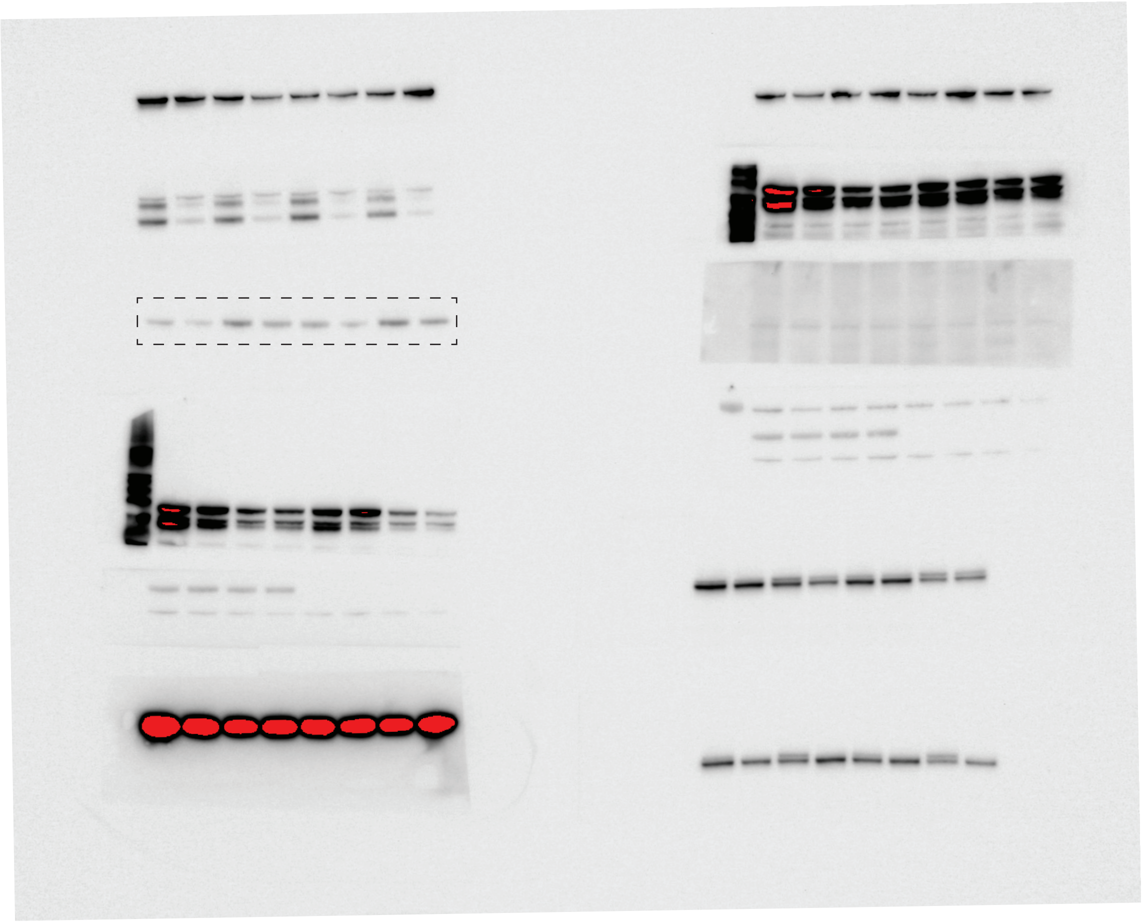

Extended Data 7f - PCNA

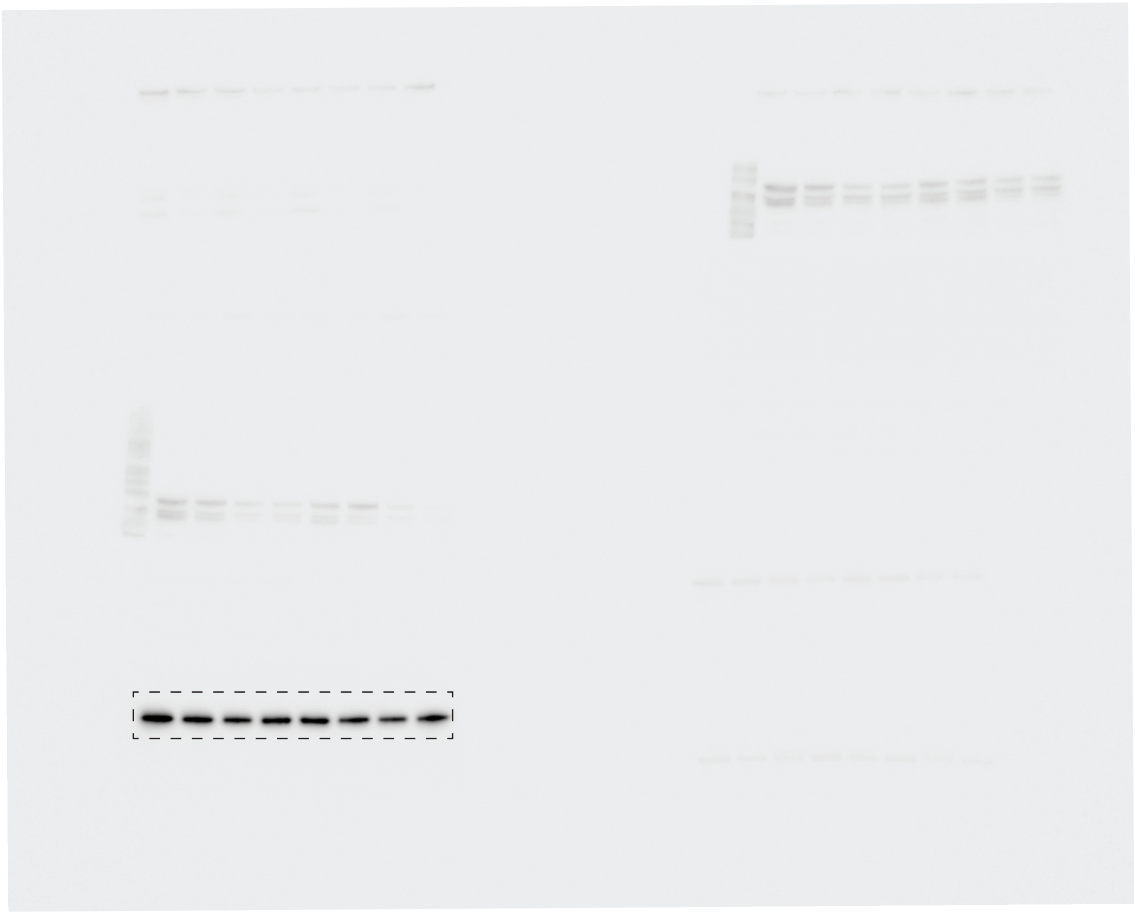

Extended Data 7f - SMC1

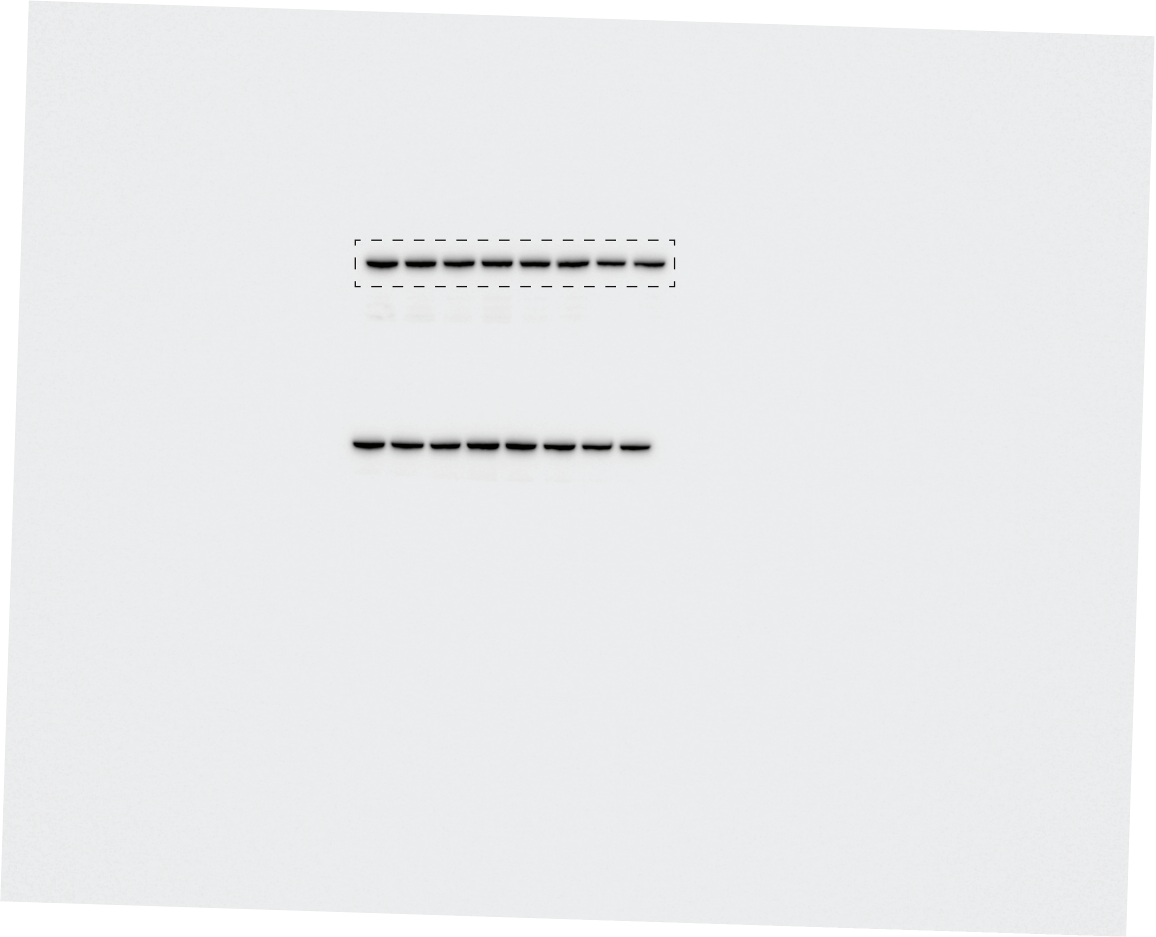

Extended Data 7f - ponceau

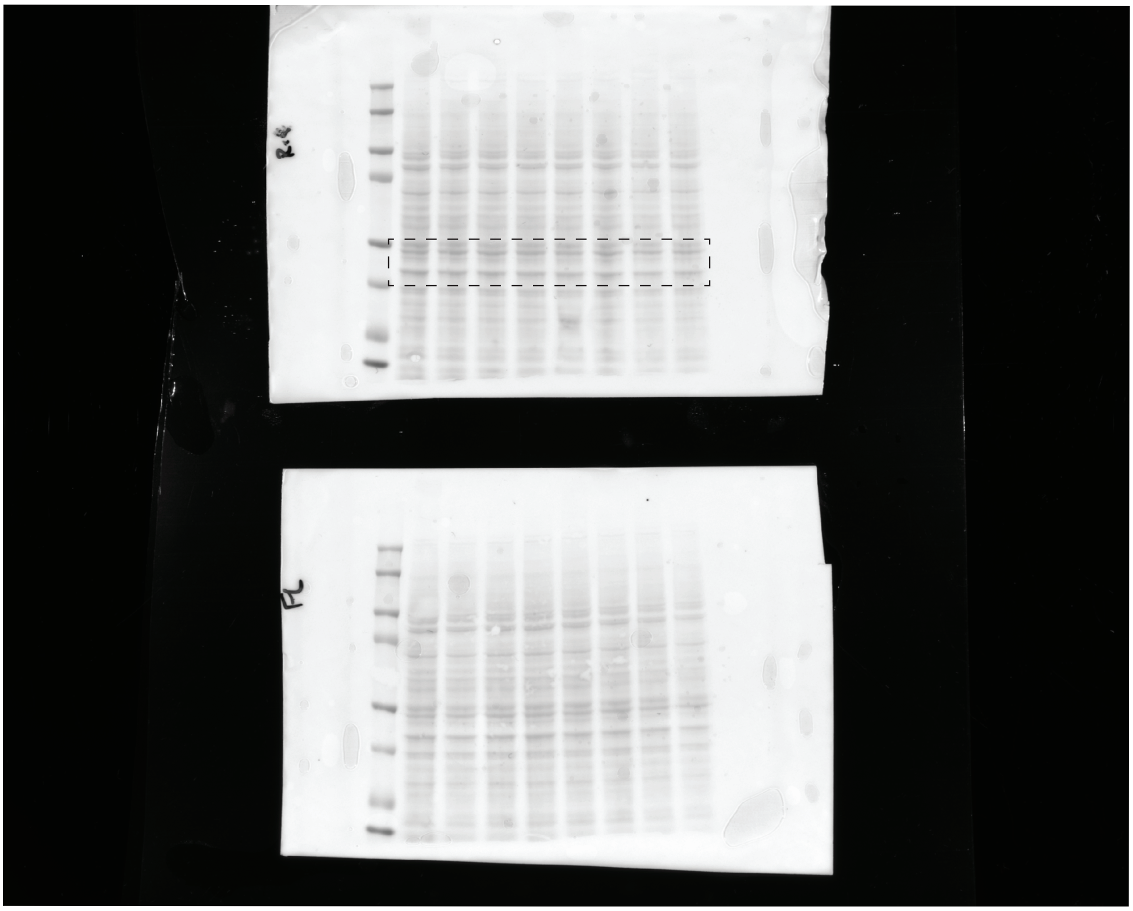

Extended Data 7g - PRIMPOL

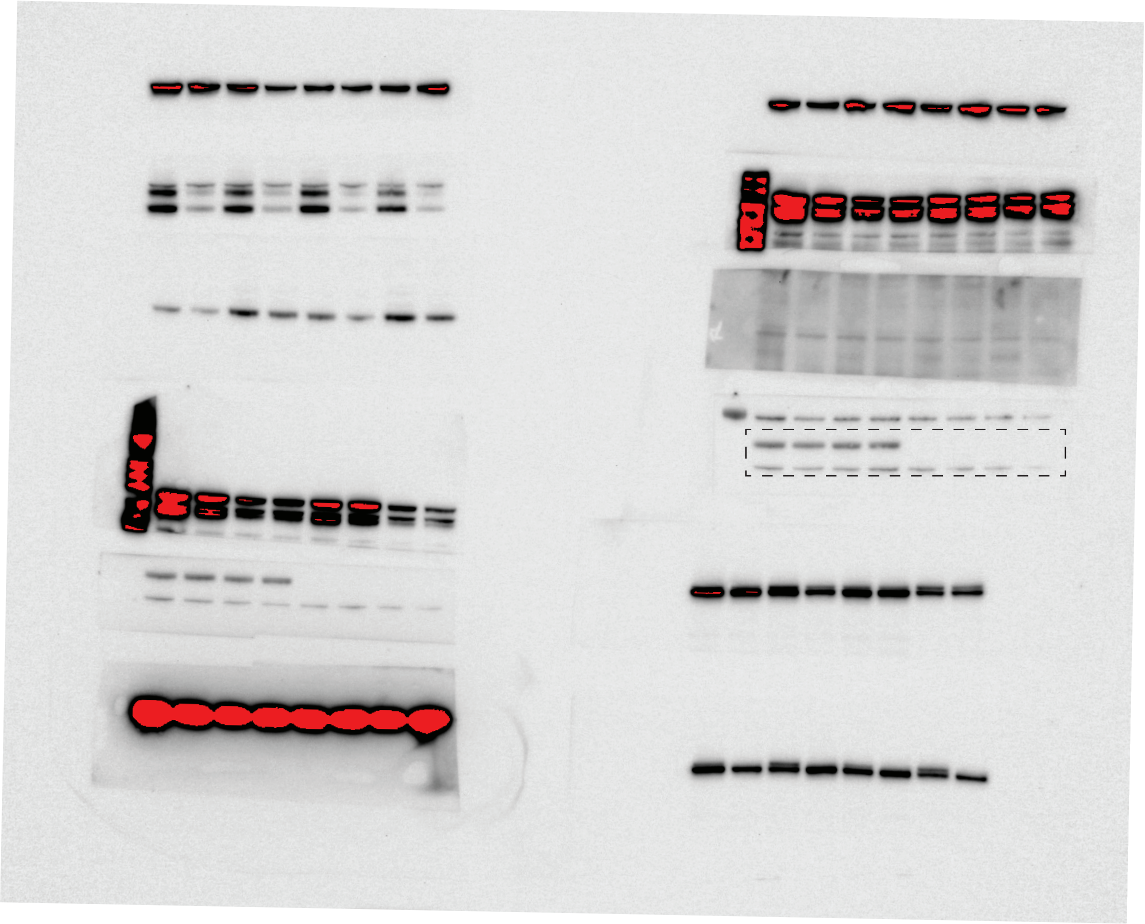

Extended Data 7g - FANCL

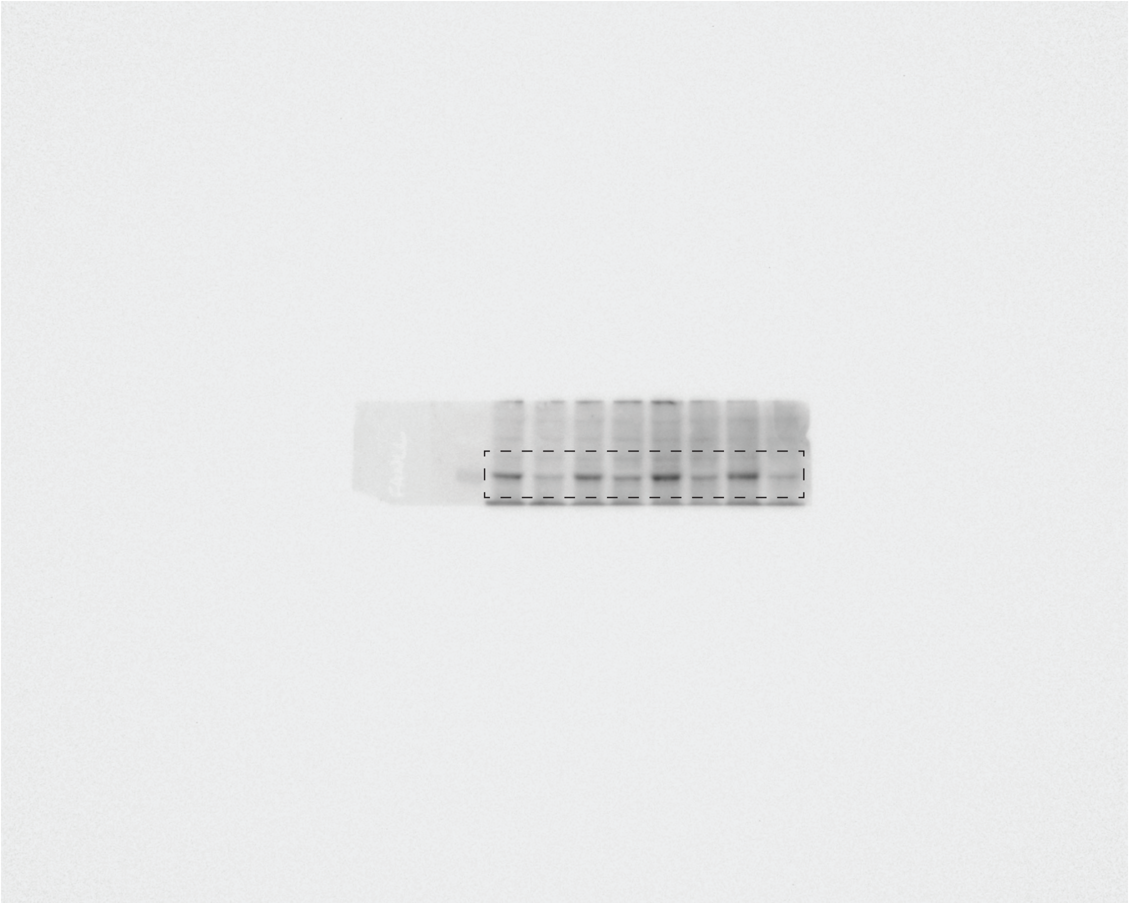

Extended Data 7g - USP1

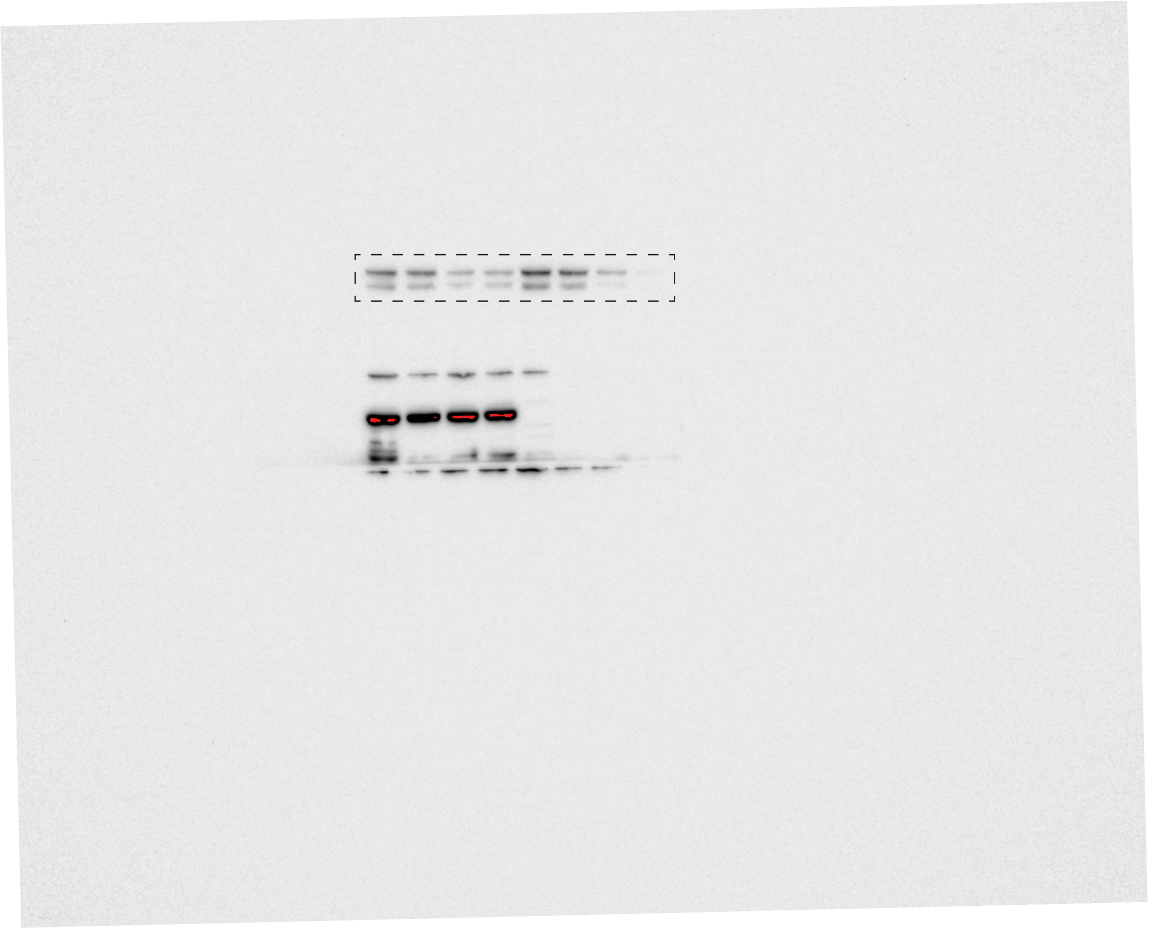

Extended Data 7g - PCNA-Ub

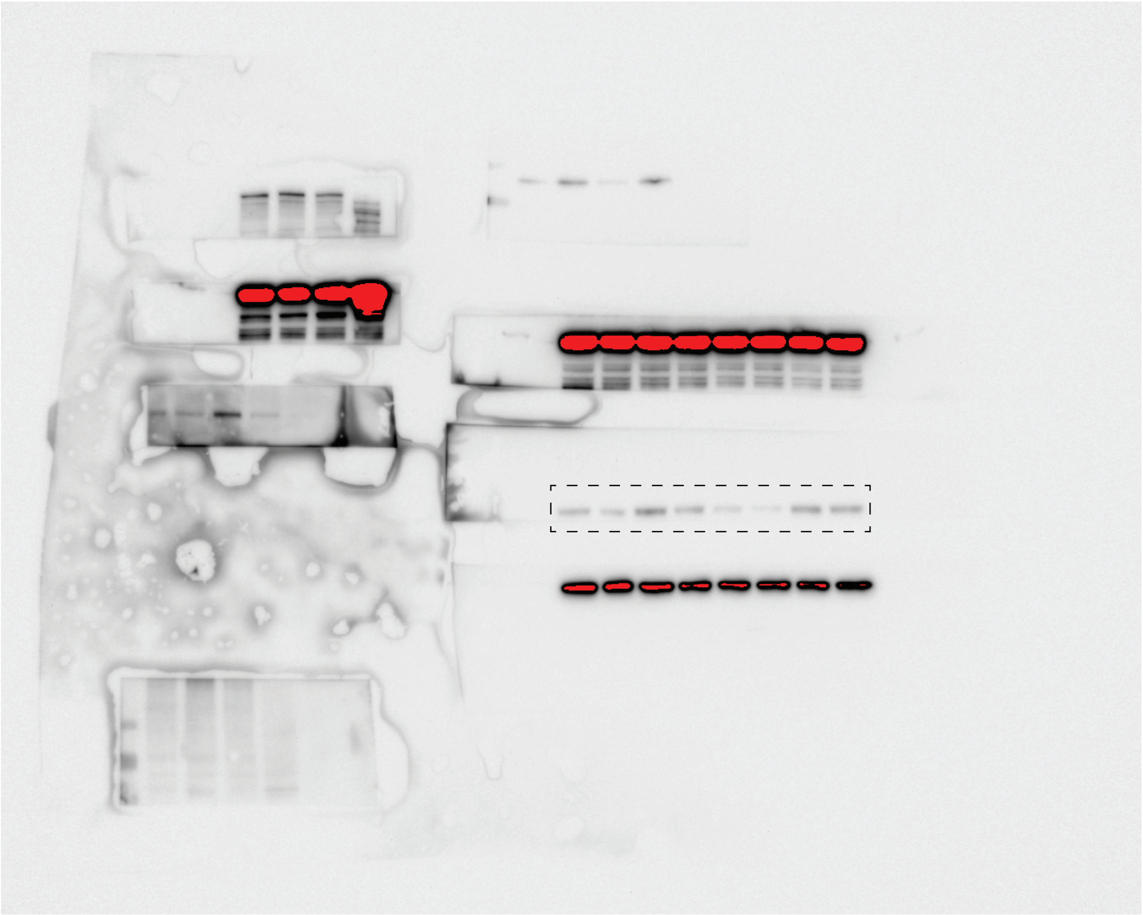

Extended Data 7g - PCNA

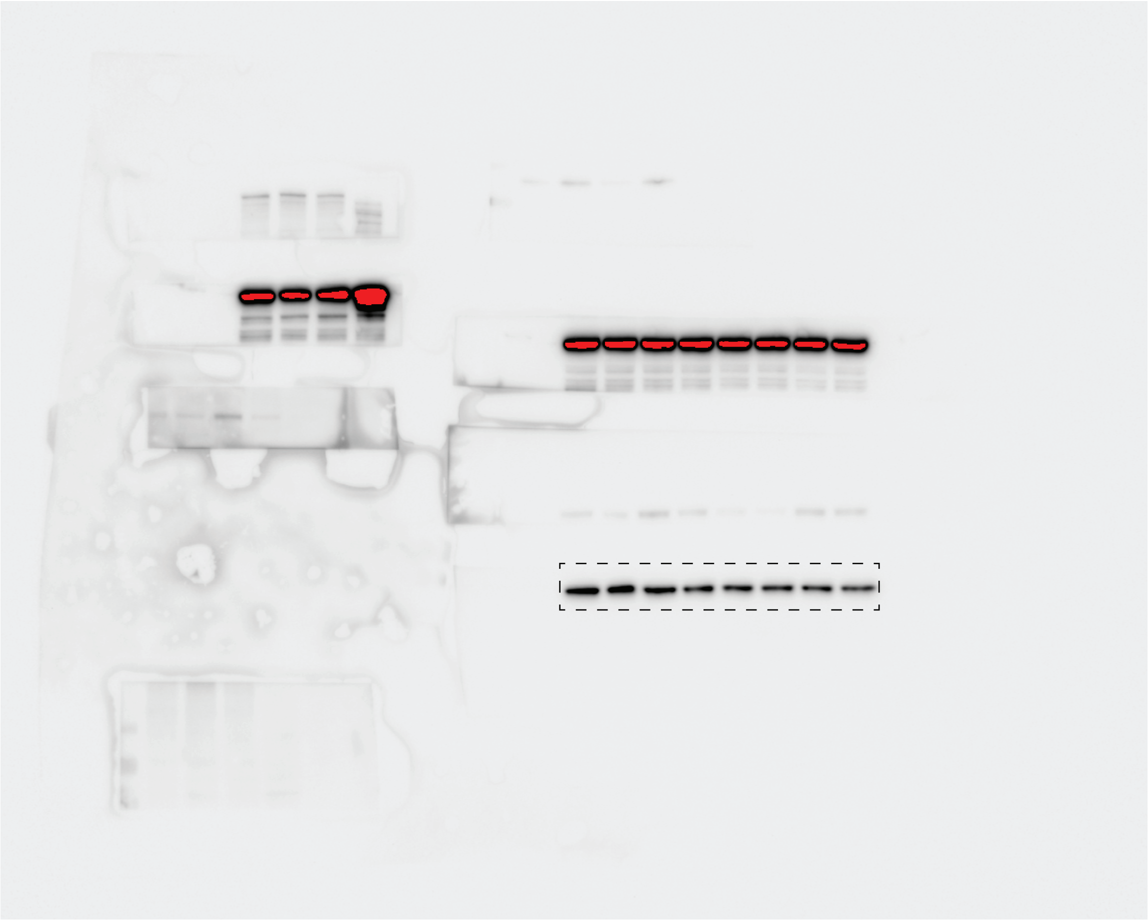

Extended Data 7g - FANCD2

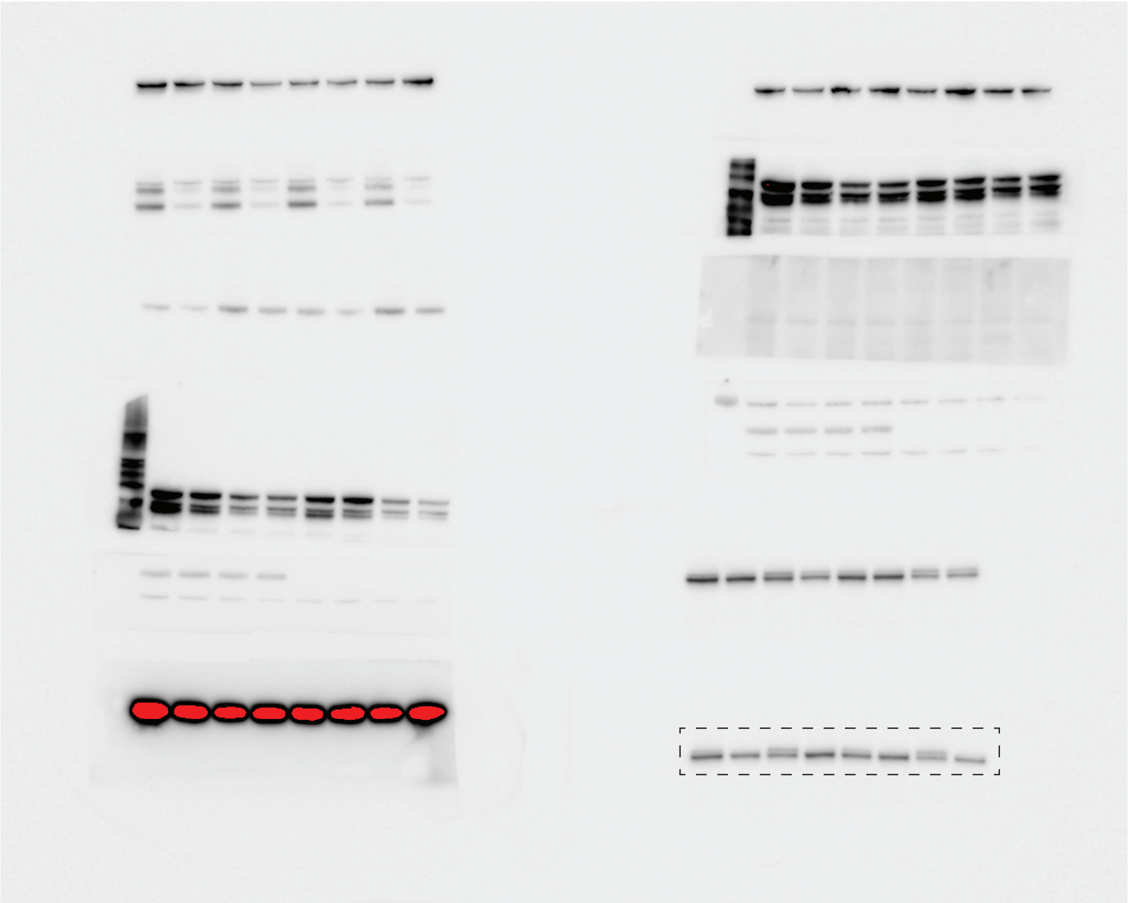

Extended Data 7g - SMC1

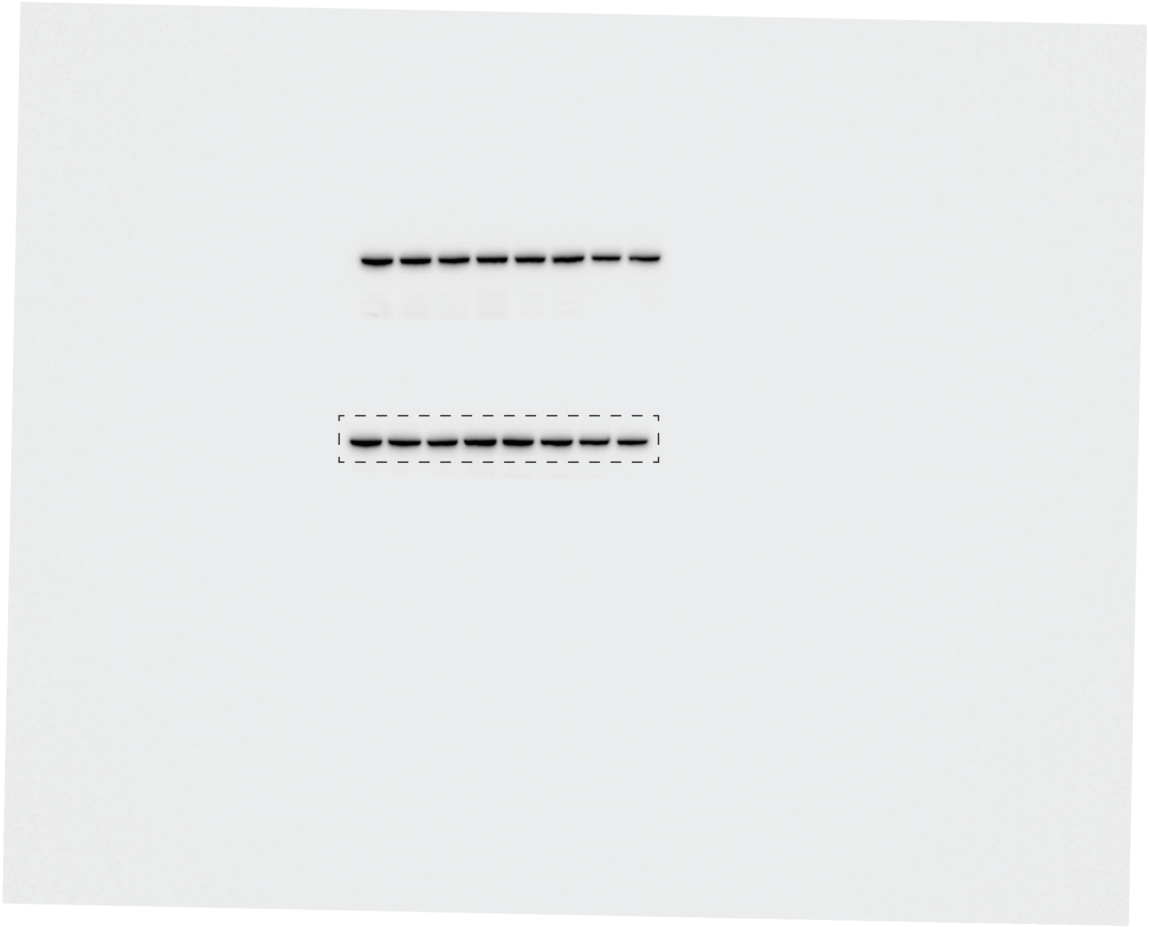

Extended Data 7g - ponceau

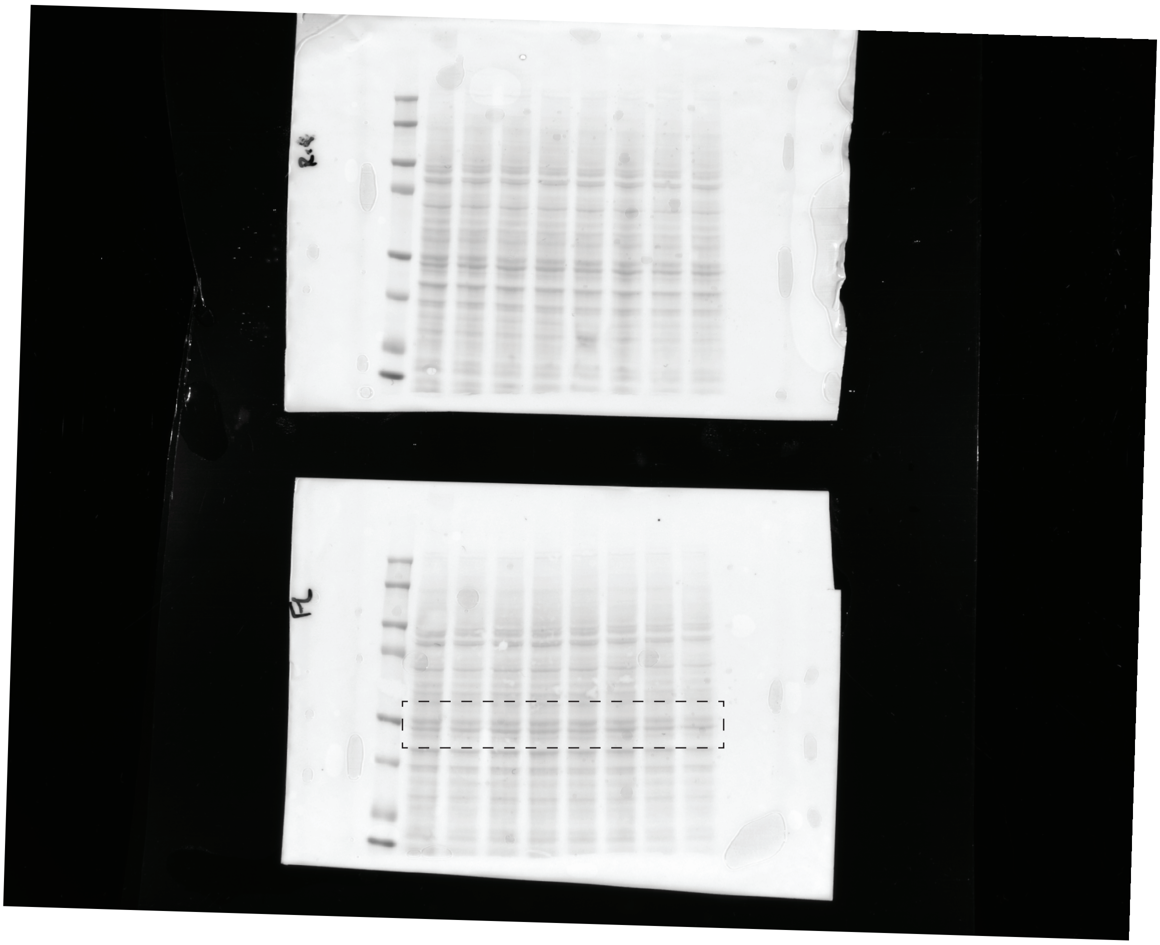

Supplement: Supplementary file 3 — Unprocessed western blots. [file 41556_2025_1852_MOESM3_ESM.pdf]
